# PACSIN2-mediated synaptic injury contributes to behavioral disorders caused by chronic stress

**DOI:** 10.1038/s41401-026-01790-0

**Published:** 2026-05-07

**Authors:** Chang-min Wang, Ye Li, Yu-hang Yi, Xiao Chen, Tian Lan, Hai-yan Lou, Wen-cheng Jian, Mei-jian Wang, Shu-yan Yu

**Affiliations:** 1 Department of Physiology, School of Basic Medical Sciences, Cheeloo College of Medicine, Shandong University, Jinan, 250012 China; 2 Shandong Key Laboratory of Mental Disorders and Intelligent Control, School of Basic Medical Sciences, Cheeloo College of Medicine, Shandong University, Jinan, 250012 China; 3 Department of Pharmacology, School of Basic Medical Sciences, Cheeloo College of Medicine, Shandong University, Jinan, 250001 China; 4 Department of Radiology, Qilu Hospital, Shandong University, Jinan, 250001 China; 5 Department of Endocrinology and Metabolism, The QiLu Hospital of Shandong University (Qingdao), Qingdao, 250012 China

**Keywords:** PACSIN2, AMPA receptor, synaptic dysfunction, chronic stress, depression

## Abstract

Major depressive disorder (MDD) is generally associated with synaptic damage in specific brain regions. However, the molecular mechanisms underlying the pathogenesis of MDD remain largely unknown. In the present study, we demonstrate that chronic stress—an established inducer of depression-like behaviors in animal models—upregulates the expression of protein kinase C and casein kinase substrate in neurons protein 2 (PACSIN2) in the hippocampal dCA1 region, a key regulator of the actin cytoskeleton and endocytic processes. The overexpression of PACSIN2 in CA1 hippocampal pyramidal neurons may increase the susceptibility to stress stimulation in rats through physical interactions with dynamin and cooperative modulation of the expression of postsynaptic membrane GluA1 AMPA receptors. Selective knockdown of PACSIN2 in the dCA1 hippocampal region of depressed rats significantly enhanced synaptic transmission, ultimately ameliorating depression-like behaviors. These findings provide direct evidence that abnormal function of hippocampal neurons resulting from perturbations in neuroplasticity may be involved in the pathogenesis of MDD. Moreover, PACSIN2 may serve as one of the underlying molecular controls through which chronic stress induces synaptic loss and dysfunction and the resulting behavioral disorders.

**Figure:**
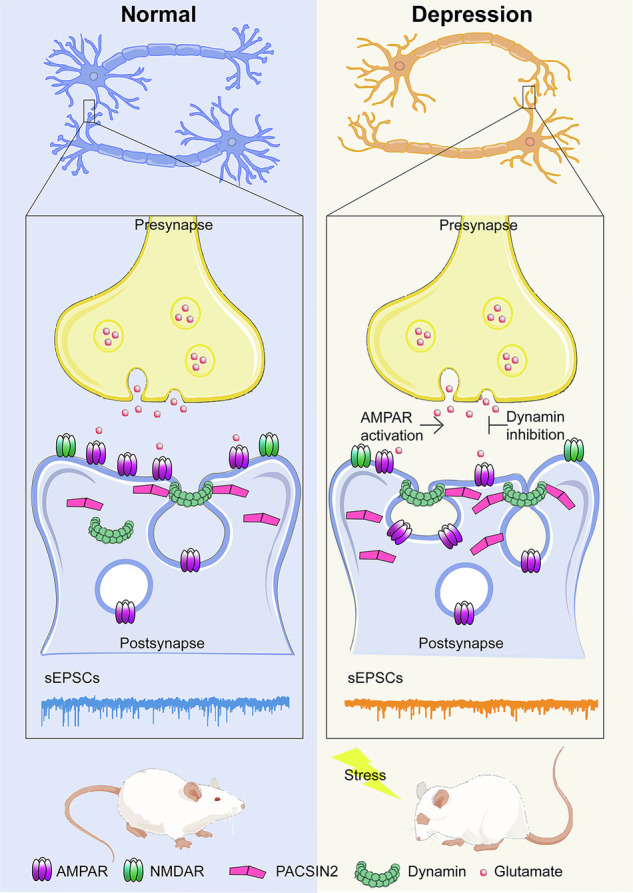

## Introduction

Major depressive disorder (MDD) is a prevalent, seriously disabling public health threat affecting as many as 12% of adults globally, with the World Health Organization predicting that it will become the second leading disease burden by 2030 [1]. The clinical symptoms of MDD include a negative mood, loss of interest, changes in appetite, and an increased likelihood of committing suicide [2]. Current antidepressants, such as serotonin/norepinephrine reuptake inhibitors (SSRIs/SNRIs), have several limitations, including delayed responses and ineffective responses in approximately 30% of patients (treatment resistant) and they do not reduce suicidal risk [3–6]. Therefore, it is necessary to clarify the underlying pathogenesis of depression and search for new therapeutic approaches.

At present, there are various hypotheses about the pathogenesis of depression, such as the genetic and epigenetic anomaly hypothesis [7, 8], the monoamine hypothesis [9], the inflammatory hypothesis [10], the neuroplasticity hypothesis and the mitochondrial theory [11]. Previous evidence has indicated that neuroplasticity is crucial for the occurrence of depression. Synaptic atrophy and a reduction in hippocampal neuronal function have been reported in a number of rodent models of chronic stress [12–14]. Recent studies have shown that the membrane capacitance, resting membrane potential and spontaneous activity of MDD iPS-neurons are reduced compared with those of cells derived from nondepressed controls [15]. In addition, several studies have suggested that a large proportion of patients with major depression do indeed have signs of hippocampal volume loss and reduced synaptic density in the hippocampus [16, 17]. Previous studies have shown that chronic administration of typical antidepressants can enhance synaptic plasticity at several levels, including increasing the birth of new neurons in the adult hippocampus and regulating synapse formation [18]. Clinical reports of rapid antidepressants, such as ketamine and esketamine, suggest a mechanism that results in rapid changes in synaptic function [19]. However, the role of synaptic injury in the pathogenesis of depression has not yet been fully elucidated.

Maintaining intrinsic neuronal excitability and normal synaptic transmission is crucial for brain development and function, environmental adaptability and the assessment and storage of complex information [20]. Synaptic function can be regulated at presynaptic sites via alterations in the release of neurotransmitter molecules or at postsynaptic sites via changes in the number, types, or properties of neurotransmitter receptors [21]. Usually, the main mechanisms controlling synaptic transmission involve changes in the number, composition, and biophysical properties of AMPA-type glutamate receptors (AMPARs) in the postsynaptic membrane [22]. AMPARs mediate the majority of excitatory neurotransmission in the brain and thus play a critical role in many aspects of brain function, including cognition, learning, and memory [23]. The number of synaptic AMPARs is tightly controlled by a dynamic process that involves the export of receptors from the endoplasmic reticulum (ER) and Golgi apparatus, exocytosis and endocytosis, and lateral diffusion of the receptors in the cell membrane [24]. Dysfunction or dysregulation of AMPARs can lead to various neurological and psychiatric disorders [25, 26]. For example, dysregulation of the endocytosis of synaptic AMPARs may contribute to cognitive impairments in Alzheimer’s disease [27]. While AMPARs are locally depleted from the cell surface through clathrin- and dynamin-dependent endocytosis or activity-dependent endocytosis [28], the underlying molecules involved in the regulation of AMPAR endocytosis, which subsequently leads to neuronal impairment, remain unclear.

Protein kinase C and casein kinase substrate in neurons protein 2 (PACSIN2), also known as synaptic dynamin-associated protein Ⅱ (syndapin Ⅱ), can remodel membranes and actin cytoskeletal processes to regulate cell-surface receptor endocytosis, endosomal trafficking, and intracellular trafficking and endocytosis [29, 30]. PACSIN2 contains a flexible N-terminal F-BAR domain, three central regions that include asparagine-proline-phenylalanine (NPF) motifs and a C-terminal Src-homology 3 (SH3) domain that binds to the endocytic machinery, such as the large GTPase dynamin and the actin-modifier neural Wiskott-Aldrich syndrome protein (N-WASP) [31, 32]. Previous studies have shown that a deficiency of PACSIN2 in Purkinje cells completely eliminates cerebellar long-term depression (LTD) in synaptic transmission, suggesting a unique function of PACSIN2 in mediating AMPAR trafficking within the cerebellum [33]. Given these findings, we investigated the possible mechanisms in a rat model of depression. Our results indicate that PACSIN2 may contribute to the dynamic trafficking of AMPA receptors and the dysfunction of synaptic transmission, which may be essential for stress-induced depression-like behaviors in rats.

## Materials and methods

### Animals

Male Wistar rats (160–180 g) were obtained from Jinan-Pengyue Experimental Animal Breeding Co., Ltd. All the experimental procedures were approved by the Ethics Committee of Shandong University (ECSBMSSDU-2022-2-65) and were in compliance with the international guidelines for animal research by the Council of International Medical Organizations. The rats were maintained in a temperature- (21 °C) and humidity-controlled (45%) environment with a 12 h light‒dark cycle (lights on from 07:00–19:00). The rats were given free access to chow and tap water for the duration of the experiment and housed in groups of no more than three rats per cage. The animals were acclimated to these laboratory conditions for one week prior to use in the experiments. The animals were randomly allocated to the experimental or control group.

### Chronic unpredicted mild stress (CUMS) model

Rats were subjected to a daily routine of CUMS to produce an animal model of depression as previously described with minor modifications [34]. The stressors consisted of 24-h water deprivation, 24-h food deprivation, 5-min cold swimming at 4 °C, 24-h wet bedding, 24-h cage tilt at 45° from the horizontal, 2-h white noise, overnight illumination, 2-min tail pinch, 3-h physical restraint and 5-min foot shock. Each stressor was randomly administered daily over a 5-week period, and no type of stressor was presented at the same time within two consecutive days to prevent the animals from anticipating the occurrence of the stimuli.

### Behavioral tests

All behavioral tests were conducted during the dark phase (19:00–23:00 h). The operators and recorders were blinded to the experimental group during the scoring.

### Sucrose preference test (SPT)

The preference of the rats for a 2% sucrose solution was tested using a two-bottle choice procedure. Each animal was housed individually during the four-day test period. On the first day, the rats were presented with two bottles of sucrose and, 24 h later, two bottles, one with sucrose and one with tap water. To prevent potential location preferences for drinking, the positions of the bottles were changed every 12 h. On the third day, the rats were deprived of food and water for 24 h and then presented with two bottles filled with either sucrose or water for 3 h. The amounts of sucrose solution and water consumed were recorded. A preference for the sucrose solution was defined as the percentage of sucrose solution consumed relative to the total fluid intake.

### Forced swimming test (FST)

The rats were placed in a cylinder of water (23–25 °C, 30 cm in diameter, 80 cm in height) for 15 min during the training phase. The depth of water was set to prevent the animals from touching the bottom with their hind limbs. Twenty-four hours after the initial training phase, the rats were placed in the cylinder for 5 min, and immobility times were recorded. Immobility was defined as no active movement except that needed to keep the animal from drowning.

### Open field test (OFT)

The rats were placed in the center of the open field test box (100 cm length × 100 cm width × 50 cm height), which was maintained under dim light, and were allowed to explore the arena for 5 min. All animal activity was recorded with a camera placed above the box. Locomotion and time spent in the center during the 5-min test period were measured with video tracking software (SMART 2.5; Panlab Harvard Apparatus, Spain). The box was wiped clean with a paper towel soaked in 75% ethanol and dried thoroughly after each test session. The total distance of horizontal locomotion and the time spent in the central area were analyzed to assess overall locomotion and anxiety-like behavior, respectively.

### Elevated plus maze (EPM) test

The apparatus for the EPM consisted of two opposing open arms and two closed arms (each arm: 50 cm length × 10 cm width) connected by a central zone (10 cm length × 10 cm width). The entire apparatus was elevated 50 cm above the floor. The rat was placed in the center zone facing one of the open arms and was allowed to freely explore the arena during a 5 min test session. The time spent in the open arm during the 5 min test and the number of entries into the open arms were measured (SMART 2.5; Panlab Harvard Apparatus, Spain). The side of the inner surface was cleaned with 75% ethanol after each animal.

### Stereotaxic surgery

The rats were anesthetized (sodium phenobarbital, 30 mg/kg, i.p.) and placed in a stereotaxic instrument (Stoelting, USA). All skull measurements were made relative to those of Bregma, and viral injections were performed using a microinjection needle with a 5-μl microsyringe (Gaoge) to deliver the virus at a flow rate of 0.08 μl/min using a microsyringe pump (Stoelting, USA). The stereotaxic coordinates (mm) for bilateral hippocampal CA1 region injection were anterior–posterior (AP) 3.24, medial–lateral (ML) 1.8 and dorsal–ventral (DV) 3.00. After injection, the pipette remained at the injection site for an additional 5 min and was then slowly retracted from the tissue. Following injection, the wound was sutured, antibiotics (bacitracin and neomycin) were applied to the surgical wound, and the animals were allowed to recover from anesthesia under a heat lamp. The following viruses were used: pAAV-hSyn-EGFP-MCS-3xFLAG-WPRE (1.03 × 10^13^ v.g./ml), pAAV-hSyn-EGFP-PACSIN2-3xFLAG-WPRE (2 × 10^13^ v.g./ml), pAAV-hSyn-EGFP-3xFLAG-miR30 shRNA (PACSIN2)-WPRE (target sequence: GCAGCTGAAGAAATTGCAAGA, 1.63 × 10^13^ v.g./ml), pAAV-hSyn-EGFP-3xFLAG-miR30 shRNA (NC)-WPRE (2.04 × 10^13^ v.g./ml), pAAV-CMV-GCaMP6s-P2A-nls-dTomato and pAAV-CaMKIIα-hM4D(Gi)-mCherry (7.10 × 10^11^ v.g./ml). All viruses used in this study were purchased from OBiO Technology and stored at −80 °C until use.

### Virus construction and injection

To verify that the EGFP viral vector does not affect the behavioral phenotypes of the WT or CUMS group rats, 2 µl of pAAV-hSyn-EGFP-MCS-3xFLAG-WPRE (1.03 × 10^13^ v.g. /ml) was injected into the bilateral hippocampus dCA1 region of WT group rats, and 2 µl of pAAV-hSyn-EGFP-3xFLAG-miR30 shRNA (NC)-WPRE (2.04 × 10^13^v.g./ml) was injected into the bilateral hippocampus dCA1 region of CUMS group rats. The behavioral experiments were performed 3 weeks after injection.

For chemogenetic inhibition of dCA1 glutamatergic neurons, 0.5 µl of pAAV-CaMKIIα-hM4D(Gi)-mCherry (7.10 × 10^11^ v.g./ml) or pAAV-CaMKIIα-mCherry was injected into the bilateral dCA1 region of the hippocampus. The experiments were performed 3 weeks after injection.

To downregulate PACSIN2 expression in the hippocampal dCA1 region of CUMS rats, the rats were randomly exposed to stressors for 5 weeks to induce depressive disorder. The depressed rats were subsequently screened through behavioral tests and injected with 2 µl of pAAV-hSyn-EGFP-3xFLAG-miR30 shRNA (PACSIN2)-WPRE (1.63 × 10^13^ v.g./ml) or pAAV-hSyn-EGFP-3xFLAG-miR30 shRNA (NC)-WPRE (2.04 × 10^13^ v.g./ml) into the bilateral dCA1 region of the hippocampus. The experiments were performed 3 weeks after injection.

To upregulate PACSIN2 expression in the hippocampal dCA1 of rats, 2 µl of pAAV-hSyn-EGFP-PACSIN2-3xFLAG-WPRE (2 × 10^13^ v.g. /ml) or pAAV-hSyn-EGFP-MCS-3xFLAG-WPRE (1.03 × 10^13^ v.g. /ml) was injected into the bilateral dCA1 region of the hippocampus. Behavior tests were performed 3 weeks after injection.

To determine whether overexpression of PACSIN2 could enhance responsiveness to stress stimulation, 2 µl of pAAV-hSyn-EGFP-PACSIN2-3xFLAG-WPRE (2 × 10^13^ v.g./ml) or pAAV-hSyn-EGFP-MCS-3xFLAG-WPRE (1.03 × 10^13^ v.g./ml) was injected into the bilateral dCA1 region of the hippocampus. After 3 weeks, the rats were subjected to subthreshold exposure to UMS (2 weeks). Behavior tests and other experiments were performed two weeks later.

To verify whether endocytosis is responsible for the induction of depression-like behaviors, 2 µl of pAAV-hSyn-EGFP-PACSIN2-3xFLAG-WPRE (2 × 10^13^ v.g./ml) or pAAV-hSyn-EGFP-MCS-3xFLAG-WPRE (1.03 × 10^13^ v.g. /ml) was injected into the bilateral dCA1 region of the hippocampus. After 1 week, dynasore (10 µM) was administered daily via an intracerebroventricular (i.c.v.) infusion into the rats for 4 weeks. Afterward, the rats were subjected to subthreshold exposure of UMS (2 weeks) after virus injection for three weeks. Finally, behavior tests were performed 5 weeks after virus injection.

To determine whether pharmacological activation of AMPARs could ameliorate stress-induced depression-like behaviors, rats were randomly exposed to stressors for 5 weeks to induce depressive disorder. The depressed rats were subsequently subjected to behavioral tests and treated with CX546 (20 mg/kg) or DMSO via intraperitoneal injection for seven days. The experiments were performed 1 week after injection.

To verify whether CX546 activated AMPARs, rats were randomly exposed to stressors for 5 weeks to induce depressive disorder. Afterward, the depressed rats were screened through behavioral tests and injected with 2 µl of pAAV-CMV-GCaMP6s-P2A-nls-dTomato into the bilateral dCA1 region of the hippocampus. Afterward, the rats were treated with CX546 (20 mg/kg) or DMSO via intraperitoneal injection for 7 days after virus injection for 3 weeks. The rats were sacrificed after the final injection.

### Intracerebroventricular injection

For intracerebroventricular injections, the rats were anesthetized (sodium phenobarbital, 30 mg/kg, i.p.) and placed in a stereotaxic apparatus. The skin on the top of the rat’s head was incised, and the area was disinfected with iodophor. The stereotaxic coordinates (mm) for the right lateral ventricle infusions were anterior–posterior (AP) 1.5, medial-lateral (ML) 1 and dorsal-ventral (DV) 3.5. A guide cannula was inserted into the right lateral ventricle and fixed with dental cement. Seven days later, a total volume of 5 μL of dynasore (10 μM) or 5 μL of normal saline (NS) was infused daily for 4 weeks through a PE tube connected to a microsyringe (Gaoge, Shanghai). To prevent backflow, the cannula remained in place for an additional 5 min and was then slowly withdrawn.

### Drug treatments

The AMPA receptor agonist CX546, the dynamin inhibitor dynasore and CNO were obtained from MedChemExpress Co., Ltd. Fluoxetine was purchased from Shanghai Aladdin Biochemical Technology Co., Ltd. CX546 (20 mg/kg) was administered via intraperitoneal injection for seven days after the CUMS procedure according to the protocols used in previous studies [35]. CNO (5 mg/kg) was injected intraperitoneally at 3 weeks after AAV2/9-CaMKIIα-hM4D(Gi)-mCherry transfection, and behaviors were tested 30 min after CNO injection. Dynasore (10 µM) was administered daily via an intracerebroventricular (i.c.v.) infusion into rats transfected with pAAV-hSyn-EGFP-PACSIN2-3xFLAG-WPRE for 4 weeks at a dose based on the results of a previous study [36]. Fluoxetine (10 mg/kg) was dissolved in saline and administered daily for 2 weeks via intraperitoneal (i.p.) injection 60 min prior to CUMS [37].

### Sparse labeling and morphological characterization analyses

To sparsely label pyramidal neurons in the dCA1 hippocampus, the pAAV-PTRE-tight-NLS-Cre-WPRE (1.28 × 10^13^ v.g./ml) and AAV-hSyn-DIO-tTA-P2A-mScarlet-WPRE (1.20 × 10^10^ v.g./ml) viruses were combined and injected into the bilateral dCA1 region of the hippocampus. The rats were euthanized 3 weeks after virus injection via anesthetization followed by perfusion with heparin sodium saline and 4% paraformaldehyde (PFA). Frozen coronal hippocampal sections were cut at a thickness of 40 μm with a freezing microtome (CM1860; Leica). The sections were incubated with DAPI (Beyotime Biotechnology C1002) for 5 min. Image acquisition was performed using confocal laser scanning microscopy (LSM880; Carl Zeiss, Germany) with a minimal z-stack distance of 0.5 μm. Imaris software (v.7.4.2, Bitplane) was used to reconstruct the neuronal dendritic arbors and dendritic spines. Spines whose mean head width was larger than the doubled mean width of the neck were classified as the mushroom type. Dendritic spine density was calculated as the number of dendritic spines per 10 μm of dendrite length.

### Western blotting

The rats were anesthetized with an intraperitoneal injection of sodium phenobarbital (30 mg/kg), and the hippocampal dCA1 region was carefully dissected for Western blot analysis 24 h after the behavioral tests. For total protein extraction, the tissues were homogenized in ice-cold RIPA buffer (Solarbio, R0020) supplemented with protease/phosphatase inhibitors (Solarbio, P1260) and PMSF (Beyotime, ST506-2). For membrane fraction extraction, the hippocampal dCA1 region or neurons were lysed using a Membrane and Cytosol Protein Extraction Kit (Thermo Fisher Scientific, 89842) according to the protocols provided. The protein concentrations in the lysates were subsequently measured with a BCA protein assay kit (CWBIO, CW0014s). Equal amounts of protein in the mixtures were separated by SDS‒PAGE and transferred to PVDF membranes for primary antibody incubation at 4 °C overnight. Antibodies against GAPDH (Proteintech, 10494-1-AP, 1:5000), Na^+^/K^+^-ATPase (Proteintech, 14418-1-AP, 1:10,000), GluA1 (Santa Cruz Biotechnology, SC555509, 1:500), PACSIN2 (Santa Cruz Biotechnology, sc-390136, 1:500), Dynamin (Santa Cruz Biotechnology, SC17807, 1:500), GluA2 (Abways Technology, CY7063, 1:1000), GluN2A (Abways Technology, CY6968, 1:1000) and GluN1 (Abways Technology, CY6798, 1:1000) were used. Peroxidase-conjugated goat anti-rabbit IgG (Zhongshan Golden Bridge Biotechnology, ZB-2301, 1:4000) and Peroxidase-conjugated goat anti-mouse (Zhongshan Golden Bridge Biotechnology, ZB-2305, 1:6000) antibodies were used as labeled secondary antibodies. Blots were detected using an enhanced chemiluminescence kit (Vazyme, E412-01). ImageJ (NIH) was used to quantify the band intensities.

### Coimmunoprecipitation (Co-IP)

Tissue from the hippocampal dCA1 region was washed in ice-cold PBS and lysed in Co-IP buffer (0.025 M Tris, 0.15 M NaCl; pH 7.2). The lysates were subsequently centrifuged at 12,000 rpm for 20 min at 4 °C. Mouse anti-PACSIN2 or anti-Dynamin was used to incubate the specific lysates at 4 °C overnight, while being continuously rotated. Protein A-agarose beads (Santa Cruz Biotechnology) prewashed in TBS (0.025 M Tris, 0.15 M NaCl; pH 7.2) were added for an additional 7 h at 4 °C while being continuously rotated. The beads were then washed three times in Co-IP buffer and three times in TBS, after which SDS-sample buffer was added to elute the proteins bound to the beads. The eluted proteins were then used for immunoblotting.

### Immunofluorescence staining

The rats were anesthetized with sodium pentobarbital and perfused transcardially with 300 ml of 0.9% NaCl containing heparin sodium salt, followed by 4% paraformaldehyde in PBS. The brains were isolated and fixed in PFA overnight, followed by dehydration (10%, 20% and 30% sucrose solution) at 4 °C. Coronal brain sections (35–40 µm) were cut using a frozen slicer (Shenzhen Ruiwode Life Technology Co., Ltd., FS800A). The brain sections and cells were then permeabilized and blocked in PBS containing 0.2% Triton X-100, 2.5% BSA, and 5% goat serum at room temperature for 1 h. The sections and cells were incubated with primary antibodies overnight at 4 °C. The antibodies used included mouse anti-PACSIN2 (Santa Cruz Biotechnology, sc-390136), mouse anti-Dynamin (Santa Cruz Biotechnology, SC17807), rabbit anti-Bassoon (Cell Signaling Technology, 6897S), rabbit anti-cFos (Cell Signaling Technology, 2250 T), rabbit anti-VGLUT1 (Bioworld Technology, BS75067), mouse anti-PSD95 (Santa Cruz Biotechnology, sc-32290), mouse anti-GluA1 (Santa Cruz Biotechnology, SC555509), rabbit anti-GluA1 (Affinity, AF6306), rabbit anti-MAP2 (Cell Signaling Technology, 4542S) and rabbit anti-PACSIN2 (Proteintech, 10518-2-AP). On the following day, the sections were incubated with Alexa Fluor 488-conjugated goat anti-mouse immunoglobulin G (IgG) (Proteintech, SA00013-1), Alexa Fluor 488-conjugated goat anti-rabbit immunoglobulin G (IgG) (Proteintech, SA00013-2), Alexa Fluor 568-conjugated donkey anti-rabbit IgG (Invitrogen, 2306809), Cy5TM-conjugated goat anti-mouse IgG (Invitrogen, 2387487) and DAPI (Beyotime, C1002) for 1 h at 37 °C. The methods for labeling surface and total GluA1 were performed as previously described with minor modifications [38–40]. For surface GluA1 staining, the transfected neurons were incubated with N-terminal GluR1 antibody (Santa Cruz Biotechnology, SC555509) for 45 min and then fixed with 4% PFA/4% sucrose for 15 min. The coverslips were blocked with 3% donkey serum and 3% BSA in PBS for 1 h and incubated with a fluorescent secondary antibody (Cy5TM-conjugated goat anti-mouse IgG, Invitrogen) for 1 h. The neurons were then permeabilized with 0.3% Triton X-100 in PBS for 10 min, blocked as above and incubated with a C-terminal GluR1 antibody (Affinity, AF6306) overnight at 4 °C, followed by incubation with a fluorescent secondary antibody and mounting to visualize total GluA1 staining. DAPI (Beyotime, C1002) was used for nuclear staining. Finally, images were captured with a confocal microscope (LSM880; Carl Zeiss, Germany) and a high-speed confocal platform (Dragonfly 200, Andor). On average, three to five images per animal from six animals were selected for analysis using ImageJ version 1.8.0 software.

According to “The rat brain in stereotaxic coordinates” (7th Edition), we defined the region of interest (ROI) as the pyramidal cell layer of the dCA1 area of the hippocampus. Next, we quantified c-Fos⁺ cells through c-Fos⁺/DAPI staining. Within the defined ROI, the number of c-Fos-positive cells and the total number of DAPI-stained cell nuclei were counted separately using image analysis software (ImageJ). The ratio was calculated as follows: The proportion of c-Fos⁺ cells = (number of c-Fos⁺ cells within the ROI/number of DAPI⁺ cells within the ROI) × 100%. Taking the c-Fos signal in the target brain region of control group rat as the reference, the mean value of the signal intensity was calculated as the base threshold, after which the c-Fos signal in the CUMS group was calculated.

### Primary neuronal cell culture and lentivirus transfection

Hippocampal neurons derived from P1 neonatal rats were first disinfected with 75% alcohol to avoid bacterial contamination. The isolated brains were immersed in 4 °C precooled buffer (2% penicillin and streptomycin in a 1:1 DMEM:F12 ratio). Meninges and blood vessels were carefully removed, and the hippocampal tissue was then placed in a new centrifuge tube containing 0.25% trypsin for 8 min at room temperature, with the solutions being mildly shaken every 1 min during digestion. An equal volume of culture solution (10% FBS, 1% penicillin and streptomycin at a 1:1 DMEM:F12 ratio; Gibco) was added to the mixture to stop the digestion. The solution was pelleted by centrifugation at 1000 rpm for 20 min. The supernatant was removed, and the cells were resuspended in a centrifuge tube with the culture solution, followed by plating on six-well plates precoated with poly-*D*-lysine (Solarbio). The six-well plates contained 1 × 10^6^ cells per well. Approximately 5 h after seeding, the culture solution was removed and replaced with neurobasal medium supplemented with 1% penicillin and streptomycin, 2% B27 and 0.5 mM glutamate. The cells were maintained at 37 °C with 5% CO_2,_ and the neurobasal medium was replaced every three days. Cytosine arabinoside (5 μM; Solarbio) was added to prevent the proliferation of nonneuronal cells on the third day. pcSLenti-EGFP-CMV-PACSIN2 was used to overexpress PACSIN2. Primary neurons at 8 days in vitro (DIV) were transiently transfected with lentiviruses and control viruses. The medium was changed 10 h after transfection. After 14 days of culture, the neurons were used for subsequent experiments.

### Hippocampal section preparation and electrophysiological recordings

Electrophysiological recordings were performed as previously described with minor modifications [41]. All electrophysiological recordings of the rats were conducted during the period (18:00–23:00). The rats were decapitated, and their brains were rapidly removed. The brain tissue was sliced into 300 μm coronal sections using a Leica VT1200S vibratome (Leica) in an ice-cold cutting solution (119 mM choline chloride, 26 mM NaHCO_3_, 7 mM MgSO_4_, 30 mM glucose, 1 mM NaH_2_PO_4_, 1 mM CaCl_2_, 3 mM sodium pyruvate, 2.5 mM KCl, 1.3 mM sodium l-ascorbate, and 1 mM kynurenic acid) and perfused with an oxygenated gas (95% O_2_, 5% CO_2_). The sections were incubated for 60 min in an oxygenated recovery solution (25 mM glucose, 85 mM NaCl, 4 mM MgCl_2_, 2.5 mM KCl, 1.25 mM NaH_2_PO_4_, 0.5 mM CaCl_2_, 24 mM NaHCO_3_ and 50 mM sucrose) and allowed to equilibrate to room temperature for whole-cell patch-clamp recordings using pCLAMP 10.6 software and the MultiClamp 700B amplifier. Whole-cell recordings were performed on hippocampal CA1 pyramidal neurons using glass pipettes (3 to 5 megohms) filled with a liquid solution (pH 7.35) containing (in mM) 130 CsMeSO_4_, 10 CsCl, 4 NaCl, 1 MgCl_2_, 5 Mg-ATP (adenosine 5′-triphosphate), 5 EGTA, 10 HEPES, 0.5 Na_3_-GTP (guanosine 5′-triphosphate), 10 phosphocreatine, and 4 QX-314. Data were filtered at 2 kHz and sampled at 10 kHz using Digidata 1440 A. For AMPAR-mediated miniature EPSC (mEPSC) recording, the neurons were voltage clamped at −70 mV, and a patch pipette was filled with an intracellular solution (in mM: 115 CsMeSO_3_, 2.8 NaCl, 20 HEPES, 0.4 EGTA, 4 ATP-Mg, 0.5 GTP-Na, 10 phosphocreatine-Na_2_, 5 TEA-Cl, and 5 QX-314 Br, pH 7.25). mEPSCs were isolated in the presence of 0.5 µM TTX, 50 µM picrotoxin, and 50 µM APV. mEPSCs were recorded at −70 mV with 100 μM picrotoxin and 0.5 μM tetrodotoxin in the ACSF. For current-clamp recordings, pipettes were filled with a potassium gluconate-based solution (in mM: 120 potassium gluconate, 20 KCl, 4 NaCl, 0.1 EGTA, 10 HEPES, 4 Na_2_-ATP, 0.3 Na_2_-GTP, 10 Na_2_-phosphocreatinine, pH 7.3, 290–310 mosmol/L ), with a final resistance of 3–5 MΩ. Evoked potentials were recorded after current stimulation was applied in steps of 20 pA, ranging from −100 pA to 100 or 300 pA. To test the efficacy of hM4D(Gi), whole-cell current-clamp recordings were performed from neurons expressing hM4D(Gi). After administration of injection currents (steps at 20 pA, ranging from −100 to 100 pA), evoked spikes were recorded before and after perfusion with CNO (5 μM). Data were analyzed with the use of the Mini Analysis Program and pCLAMP software.

### Label-free quantitative proteomics analysis

Label-free quantitative proteomics analysis was performed on rat dCA1 hippocampal samples. Proteins were isolated from tissues and then cleaved and quantified with fluorescent peptides. On the basis of the quantitative results, 1 μg samples were analyzed by liquid chromatography‒tandem mass spectrometry. Enzymolysis products were separated using capillary high-performance liquid chromatography, followed by mass spectrometry analysis using a Q Exactive HF-X mass spectrometer (Thermo Fisher Scientific). The database was downloaded from the UniProt database. The Kyoto Encyclopedia of Genes and Genomes (KEGG) database was used to annotate protein pathways. The KEGG database description of proteins was annotated by the KEGG online service tool KAAS and mapped onto the KEGG pathway database by the KEGG online service tool KEGG mapper.

### Molecular docking

To investigate the possibility of interactions between PACSIN2 and dynamin from rats, the protein structures of PACSIN2 and dynamin were obtained from the UniProt database (https://www.uniprot.org/) with the UniProt IDs of Q9QY17 and P39052, respectively, and then protein‒protein molecular docking was performed with the GRAMM-X web server (http://vakser.bioinformatics.ku.edu/resources/gramm/grammx). Of the outputs of the 10 models, the first was used as the final model. PyMOL and ligplot^+^ were used for visualization. ΔiG indicates the solvation free energy gain upon formation of the interface, in kcal/M.

### Statistical analysis

The replication and sample sizes for all the experiments are detailed in the figure legends. All the data were analyzed using GraphPad Prism software (version 8.0) and are presented as the mean ± SEM. All statistical analyses were two-tailed. The Kolmogorov–Smirnov test and Shapiro–Wilk normality test were used to determine whether parametric or nonparametric analyses should be performed. All one-factor experiments passed the normality test, and therefore, the data were analyzed with parametric tests (Student’s *t* tests, repeated measures one-way analysis of variance (ANOVA)) followed by Tukey’s multiple comparison tests. For data that failed to demonstrate a normal distribution, nonparametric tests were used. Normality tests were not performed for one-way ANOVA with missing values, sample sizes ≤4 or two-way ANOVA. Two-way ANOVAs for parametric or mixed-effects analysis (mixed-effects model (REML) with fixed effects (type III)) for nonparametric tests were used for two-factor experiments, followed by Sidak’s multiple comparison tests for two groups or Tukey’s multiple comparison tests for three groups. Investigators were partially blinded to the groups when performing the experiments and data analysis. A *P* value < 0.05 was considered to indicate statistical significance. NS: not significant, *P* > 0.05; *P  <  0.05; **P  <  0.01; ***P  <  0.001; ****P  <  0.0001. For details regarding the specific statistical analysis performed, see the figure legends and source data associated with each figure.

## Results

### CUMS produces depressive-like behaviors and decreases the intrinsic excitability of the hippocampal neurons of rats

**Fig. 1:**
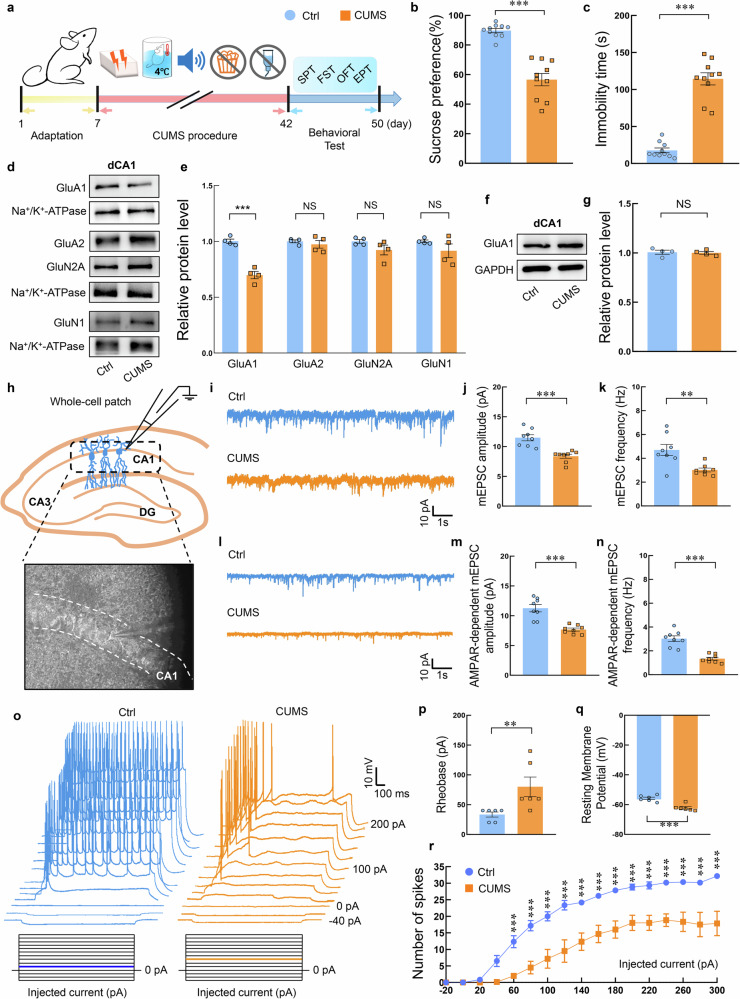
CUMS produces depressive-like behaviors and decreases the intrinsic excitability of hippocampal neurons of rats. **a** Experimental paradigm for CUMS model and determining behavioral responses of rats. **b** The measurement of sucrose preference of Ctrl and CUMS rats. *n*  =  10 rats per group. ***P < 0.001 (Two-tailed unpaired *t*-test). **c** Immobility time in FST of Ctrl and CUMS rats. *n*  =  10 rats per group. **d**, **e** The expression levels of indicated glutamate receptor proteins in the membrane fractions from dCA1region of hippocampus. *n*  =  4 rats per group. NS: not significant *P* > 0.05, ***P < 0.001 (Two-tailed unpaired *t*-test). **f**, **g** The expression levels of GluA1 subunits in the total lysates from dCA1 region of hippocampus. *n*  =  4 rats per group. NS: not significant *P* > 0.05 (Two-tailed unpaired *t*-test). **h** Whole-cell patch-clamp recording from CA1 hippocampal pyramidal neurons in brain slices of rats. **i** Representative mEPSCs traces in CA1 hippocampal pyramidal neurons. Summary data for mEPSCs amplitude (**j**) and frequency (**k**) in CA1 hippocampal pyramidal neurons of Ctrl and CUMS rats. *n*  =  8 neurons from three rats per group. **P < 0.01 (Two-tailed unpaired *t*-test). **l** Representative traces for AMPAR-mediated mEPSCs recorded at CA1 hippocampal pyramidal neurons. Summary data for AMPAR-mediated mEPSCs amplitude (**m**) and frequency (**n**) in CA1 hippocampal pyramidal neurons of Ctrl and CUMS rats. *n*  =  8 neurons from four rats per group. **P < 0.01, ***P < 0.001 (Two-tailed unpaired *t*-test). **o** Example traces of membrane potential responses to step current injections in CA1 hippocampal pyramidal neurons. Scale, 10 mV; 500 ms. **p** Quantification of the rheobase (pA) in CA1 hippocampal pyramidal neuron of Ctrl and CUMS rats. *n*  =  6 neurons from three rats per group. *P < 0.05 (Two-tailed unpaired t-test (nonparametric tests), Mann-Whitney U test). **q** Quantification of the RMP (mV) in CA1 hippocampal pyramidal neuron of Ctrl and CUMS rats. *n*  =  6 neurons from three rats per group. ***P < 0.001 (Two-tailed unpaired *t*-test (nonparametric tests), Mann-Whitney U test). **r** Quantification of AP numbers. *n*  =  6 neurons respectively, from three animals in each group. ***P < 0.001 (Two-way repeated measures ANOVA with Sidak’s multiple comparisons). Data are represented as the mean ± SEM. Ctrl control, CUMS chronic unpredictable mild stress.

We established an animal model of depression with the chronic unpredictable mild stress (CUMS) protocol (Fig. 1a). Anhedonia and behavioral despair are two key endpoints used to evaluate depressive-like states in animal models. After 5 weeks of CUMS exposure, the rats consumed significantly less sucrose solution in the sucrose preference test (SPT) (Fig. 1b) and had increased immobility times in the forced swim test (FST) (Fig. 1c). In the open field test (OFT), which can assess locomotor activity and explore behavior, CUMS rats traveled similar total distances but spent fewer times in the center area of the open field (Supplementary Fig. 1a–c). Finally, CUMS-induced rats spent less time and made fewer entries into the open arms of the maze in the elevated plus-maze (EPM) test, indicating increased anxiety-like behavior (Supplementary Fig. 1d–f). Collectively, these behavioral results provide robust evidence that this CUMS protocol induces depression-like and anxiety-like behaviors in rats.

We then examined the synaptic spine density within hippocampal CA1 pyramidal neurons using sparse labeling methods (Supplementary Fig. 1g). The total spine density and mushroom-shaped spines were significantly lower in the pyramidal cells of CUMS rats than in those of control rats (Supplementary Fig. 1h–j). We then investigated whether this CUMS treatment resulted in changes in the expression of synaptic receptors on dendritic spines. We used antibodies against the presynaptic protein Bassoon to visualize presynaptic compartments. Antibodies against GluA1 were used to label AMPA receptors (AMPARs). Compared with those in the control group, the signal intensities of GluA1 and Bassoon in the dCA1 hippocampus were decreased in CUMS rats (Supplementary Fig. 1k–m). These results suggest that CUMS exposure results in maladaptation of the synaptic structure, an effect that may subsequently contributes to the depression-like behaviors observed in these rats.

To further investigate the potential role of glutamate receptors in the hippocampus, we isolated total and membrane-bound protein fractions from the dCA1 area of the hippocampus. We found that the levels of GluA1 receptors, but not those of GluA2, GluN1 or GluN2A receptors, were significantly decreased in the membrane fractions of CUMS rats (Fig. 1d, e). However, the total levels of GluA1 protein within the dCA1 area of the hippocampus did not significantly differ between CUMS and control rats (Fig. 1f, g). To determine whether these structural synaptic deficits were accompanied by functional changes in CA1 pyramidal neurons, we first detected a significant reduction in c-Fos-positive cells in CUMS rats (Supplementary Fig. 2a–c). Furthermore, whole-cell patch-clamp recordings of brain sections revealed that CUMS rats presented decreased frequencies and amplitudes of spontaneous excitatory postsynaptic current (sEPSC) (Supplementary Fig. 2d–f) and miniature excitatory postsynaptic current (mEPSC) (Fig. 1h–k). To further evaluate AMPAR-mediated synaptic transmission, we recorded AMPAR miniature excitatory synaptic currents (mEPSCs). Both the amplitude and the frequency of AMPAR-dependent mEPSCs decreased in CUMS rats (Fig. 1l–n). Moreover, the current‒voltage relationship, as observed within representative CA1 hippocampal pyramidal neurons, revealed that the firing threshold was significantly greater in the CUMS group than in the control group (Fig. 1o, p). CUMS significantly depolarized the resting membrane potential (RMP) (Fig. 1q) and decreased the AP threshold (Supplementary Fig. 2g) of hippocampal CA1 pyramidal neurons in rats. In addition, the frequencies of evoked action potentials (APs), as obtained at different steps of current, decreased in the neurons of CUMS rats (Fig. 1r). Neurons from CUMS rats displayed significantly lower input resistance and membrane capacitance and longer half-width than those from controls did (Supplementary Fig. 2h–j). These results demonstrated that CUMS exposure decreased the surface expression of GluA1 receptors and thus may have impaired the synaptic transmission function of hippocampal CA1 pyramidal neurons.

To investigate whether the hypoactivity of pyramidal neurons contributes to the induction of depressive-like behaviors, we directly suppressed the activity of CA1 pyramidal neurons using chemogenetics (Supplementary Fig. 3a, b). Behaviorally, compared with preadministration, intraperitoneal (i.p.) administration of CNO to hM4Di-expressing rats significantly induced depressive-like and anxiety-like behaviors, as revealed by increased immobility times in the FST (Supplementary Fig. 3c), reduced sucrose preference in the SPT (Supplementary Fig. 3d), decreased exploration of the center area in the OFT (Supplementary Fig. 3e, f) and reduced time spent and number of entries into the open arms of the EPM (Supplementary Fig. 3g, h). Section recordings (Supplementary Fig. 3i) indicated that clozapine-N-oxide (CNO; 10 μM) significantly increased the thresholds needed to evoke action potentials (Supplementary Fig. 3j, l), reduced input resistance (Supplementary Fig. 3m), and decreased the number of spikes, as assessed under step injections of current in hM4Di-expressing hippocampal CA1 pyramidal neurons (Supplementary Fig. 3n–o). However, intraperitoneal (i.p.) administration of CNO to mCherry-expressing rats did not induce depressive-like behaviors or reduce the activity of excitatory pyramidal neurons. Therefore, these data indicate that acutely suppressing the activity of excitatory pyramidal neurons within CA1 pyramidal neurons may induce depressive phenotypes in rats.

### CUMS increases PACSIN2 expression in the CA1 hippocampal region

**Fig. 2:**
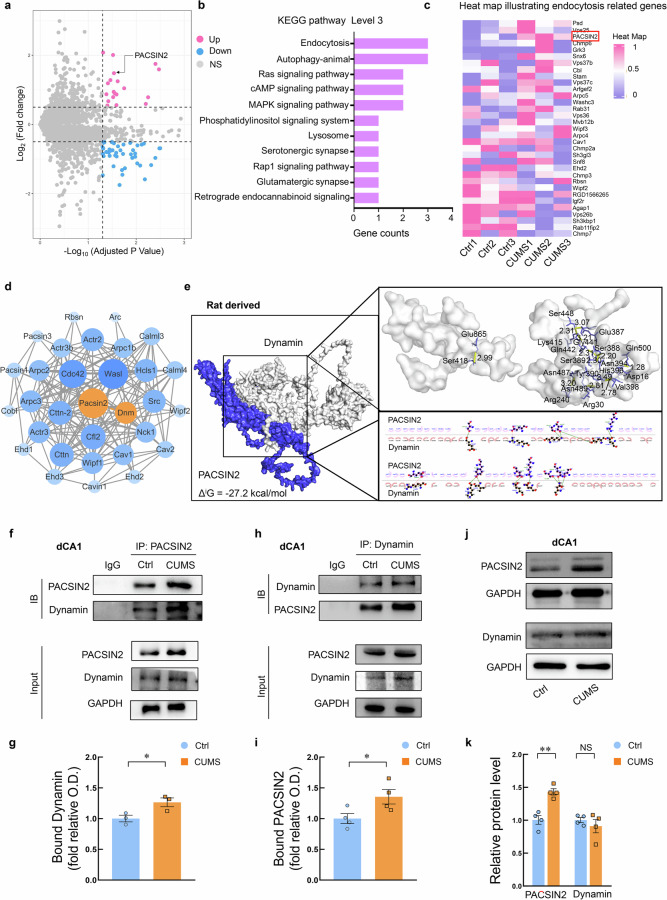
CUMS increases PACSIN2 protein expression in CA1 hippocampal regions. **a** Volcano plots indicating differentially expressed proteins. FC, fold change. **b** Histogram of Kyoto Encyclopedia of Genes and Genomes (KEGG) pathways of the differentially expressed proteins at level 3. **c** Heatmap diagram of all proteins in proteomic analysis illustrating endocytosis related genes. PACSIN2: log*Fc* = 1.48, *P* = 0.028981. **d** The protein-protein interaction (PPI) network of PACSIN2 and dynamin (STRING 11.5, PPI Networks and Enrichment Analysis. https://string-db.org/). **e** The possible interaction mode of PACSIN2 and dynamin derived from rats (Δ^i^*G *= -27.2 kcal/mol), Δ^i^*G* indicates the solvation free energy gain upon formation of the interface, in kcal/mol. PyMOL and ligplot^+^ were used for visualization. **f**, **h** Representative co-immunoprecipitation (co-IP) of PACSIN2, dynamin, and GAPDH of the dCA1 region of hippocampus in rats. **g**, **i** Co-IP analysis of PACSIN2 and dynamin in rats. dynamin: *n*  =  3 rats per group; PACSIN2: *n*  =  4 rats per group. *P < 0.05 (Two-tailed unpaired *t*-test). **j**, **k** Representative immunoblots and quantitative analyses of PACSIN2 and dynamin of the dCA1 region of hippocampus in the Ctrl and CUMS rats. PACSIN2: *n*  =  4 rats per group; dynamin: *n*  =  4 rats per group. NS: not significant *P* > 0.05, ***P < 0.001 (Two-tailed unpaired *t*-test). Data are represented as the mean ± SEM. Ctrl control, CUMS chronic unpredictable mild stress rats.

The above findings indicated that CUMS impairs both synaptic structure and transmission function; thus, we next explored potential targets that may be involved in CUMS-induced synaptic injury and depression-like behaviors. Proteomic analysis of dCA1 hippocampal tissue revealed the genes whose expression differed between the CUMS group and the control group (Fig. 2a, Supplementary Fig. 4a). The results from the enrichment analysis of Kyoto Encyclopedia of Genes and Genomes (KEGG) pathways indicated that the functional enrichment of differentially expressed genes (DEGs) was mostly related to endocytosis, glutamatergic synapses, lysosomes and autophagy (Fig. 2b, Supplementary Fig. 4b, c).

Extensive data from previous studies indicate that the core mechanisms regulating synaptic transmission involve alterations in the number, subunit composition, and biophysical properties of AMPA-type glutamate receptors (AMPARs) at the postsynaptic membrane [22]. It is known that the trafficking of AMPARs from the synaptic plasma membrane into intracellular compartments occurs predominantly through endocytosis, and we found that the levels of GluA1 receptors in the membrane fraction but not the total levels of GluA1 protein in the dCA1 area of the hippocampus were reduced. Thus, we hypothesize that dysregulation of AMPAR dynamic neuronal trafficking, especially the process of endocytosis, may be critical for the pathogenesis of depressive behaviors. These findings prompted us to investigate the molecules that may be involved in the endocytosis of GluA1 receptors. A heatmap analysis of the proteins associated with endocytosis between the control and CUMS groups revealed that protein kinase C and casein kinase substrate in neurons protein 2 (PACSIN2) were significantly increased in the dCA1 hippocampal regions of CUMS rats (Fig. 2c). Previous studies have demonstrated that PACSIN2 (a member of the PACSIN/syndapin family of membrane trafficking adaptors) interacts with AMPAR subunits and modulates their endocytosis, exocytosis, and synaptic surface expression [33]. Therefore, we investigated whether PACSIN2 is involved in the endocytosis of AMPARs, thereby regulating synaptic transmission and contributing to the pathogenesis of depressive-like behaviors. The results revealed that the expression of PACSIN2 was significantly increased in the dCA1 region of the hippocampus of CUMS rats (Supplementary Fig. 5a, b). As dynamin, a neuron-specific guanosine triphosphatase (GTPase), may play a key role in regulating processes related to endocytosis, we next investigated whether dynamin is a potential target molecule of PACSIN2. The results of our protein–protein interaction (PPI) network analysis indicated that a direct interaction between dynamin and PACSIN2 may exist (Fig. 2d). Moreover, molecular docking results suggested the possibility of an interaction between PACSIN2 and dynamin derived from rats. The results showed that PACSIN2 may structurally interact with dynamin (Fig. 2e). The left figure shows the interaction mode of PACSIN2 and dynamin, and the two figures on the right show the interactions across their interface. The amino acid residues Ser418, Glu369, Gly441, Ser448, Arg240, Glu440, Tyr390, Asp16, Arg30, Gln442, Ser389 and Ser388 of PACSIN2 form hydrogen bonds with the Glu865, Arg440, Lys415, Glu387, Asn487, Arg386, His396, Val398, Gln500, Asn489 and Asn394 residues of dynamin, respectively, and hydrophobic interactions may occur between other residues, as shown in the figure. Coimmunoprecipitation verified that PACSIN2 interacted with dynamin and revealed that the degree of interaction between PACSIN2 and dynamin increased in CUMS rats (Fig. 2f–i). No significant differences in the protein levels of dynamin were detected between control and CUMS rats, as determined by Western blotting (Fig. 2j, k). Finally, the results revealed that the classical antidepressant fluoxetine [42] significantly ameliorated depression-like behaviors, which were accompanied by a downregulation of PACSIN2 expression in CUMS rats (Supplementary Fig. 5c–4h). Interestingly, the results demonstrated that CUMS exposure did not alter PACSIN2 expression in either the mPFC (Supplementary Fig. 6a, b) or the BLA (Supplementary Fig. 6c, d). Together, these results indicate that the elevated levels of PACSIN2 in the dCA1 region of the hippocampus induced by CUMS may be a critical risk factor for depression.

### Overexpression of PACSIN2 induces GluA1 internalization and promotes susceptibility to stress in rats

**Fig. 3:**
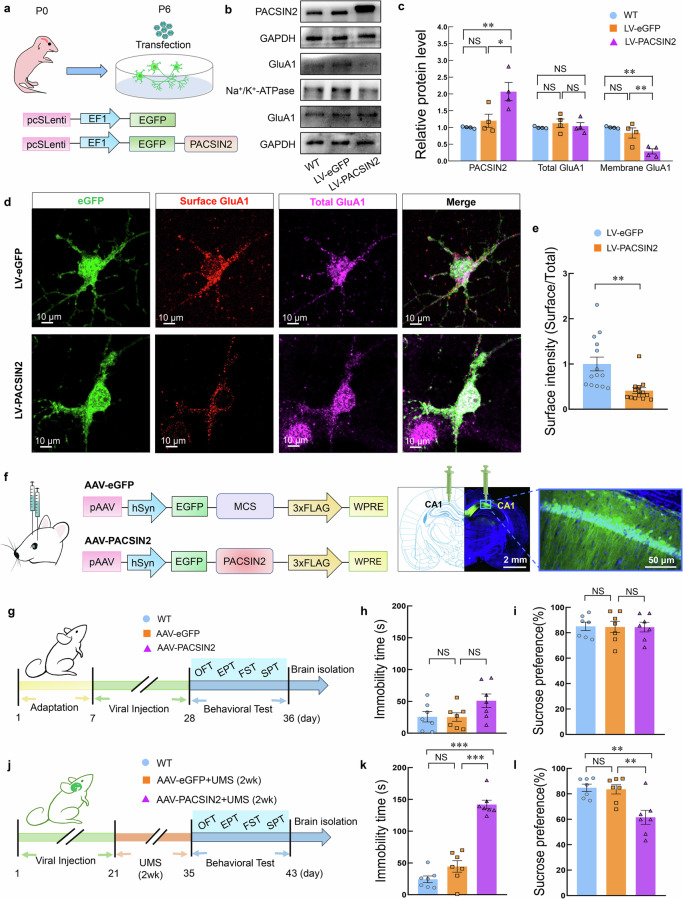
Overexpression of PACSIN2 induces GluA1 internalization and promotes susceptibility to stress in rats. **a** Representative diagrams of primary neurons transfected with lentiviruses. **b**, **c** Representative immunoblots and quantitative analyses of PACSIN2, total GluA1, membrane GluA1 in the primary cultured neurons after PACSIN2 overexpression. PACSIN2: *n* = 4 for each group; total GluA1: *n* = 4 for each group; membrane GluA1: *n* = 4 for each group. NS: not significant *P* > 0.05, *P < 0.05, **P < 0.01(One-way ANOVA with Tukey’s multiple comparison tests). **d** Example confocal images of primary neurons in LV-eGFP and LV-PACSIN2 groups. Scale bars, 2 μm. **e** Bar graphs show relative surface GluA1 intensity normalized against only eGFP transfection control. *n*  =  14 per group. **P < 0.01 (Two-tailed unpaired *t*-test (nonparametric tests), Mann-Whitney U test). **f** Schematics of AAV vectors and bilateral injection sites in the hippocampus. Scale bar, 2 mm (left) and 50 μm (right). **g** Experimental design studies. **h** Immobility time of rats in FST. *n*  =  7 rats each group. NS: not significant *P* > 0.05 (One-way ANOVA with Tukey’s multiple comparisons test). **i** The measurement of sucrose preference of rats. *n*  =  7 rats each group. NS: not significant *P* > 0.05 (One-way ANOVA with Tukey’s multiple comparisons test). **j** Schematic diagram of experimental procedure. **k** Immobility time of rats in FST. *n*  =  7 rats each group. NS: not significant *P* > 0.05, ***P < 0.001 (One-way ANOVA with Tukey’s multiple comparisons test). **l** The measurement of sucrose preference of rats. *n*  =  7 rats each group. NS: not significant *P* > 0.05, **P < 0.01 (One-way ANOVA with Tukey’s multiple comparisons test). Data are represented as the mean ± SEM.

To test the potential function of PACSIN2 in synaptic AMPARs, primary cultured neurons were transfected with lentiviruses to overexpress PACSIN2 (Fig. 3a–c and Supplementary Fig. 7a). The results revealed that the overexpression of PACSIN2 decreased surface GluA1 levels in primary cultured neurons, whereas total GluA1 protein levels were not significantly affected (Fig. 3b–e). Moreover, this overexpression of PACSIN2 also increased the colocalization of PACSIN2 and dynamin in neurons (Supplementary Fig. 7b, c). Interestingly, we found that overexpression of PACSIN2 decreased the density of vesicular glutamate transporter 1 (VGLUT1) and postsynaptic density protein-95 (PSD-95) colocalization in neurons, indicating that upregulation of PACSIN2 expression decreased the number of functional excitatory synapses (Supplementary Fig. 7d, e). Taken together, these results demonstrate that PACSIN2 may promote GluA1 internalization via its colocalization with dynamin in postsynaptic neurons.

To verify that the EGFP viral vector does not affect the behavioral phenotypes of the rats, we first injected the constructed EGFP virus into the bilateral hippocampal CA1 region of the WT or CUMS group rats. Then, behavioral experiments were performed 3 weeks after injection (Supplementary Fig. 8a, j). The results revealed that the EGFP virus did not significantly affect depression-like behaviors (Supplementary Fig. 8b, c; Supplementary Fig. 8k, l) or anxiety-like behaviors (Supplementary Fig. 8d–i; Supplementary Fig. 8m–r).

**Fig. 4:**
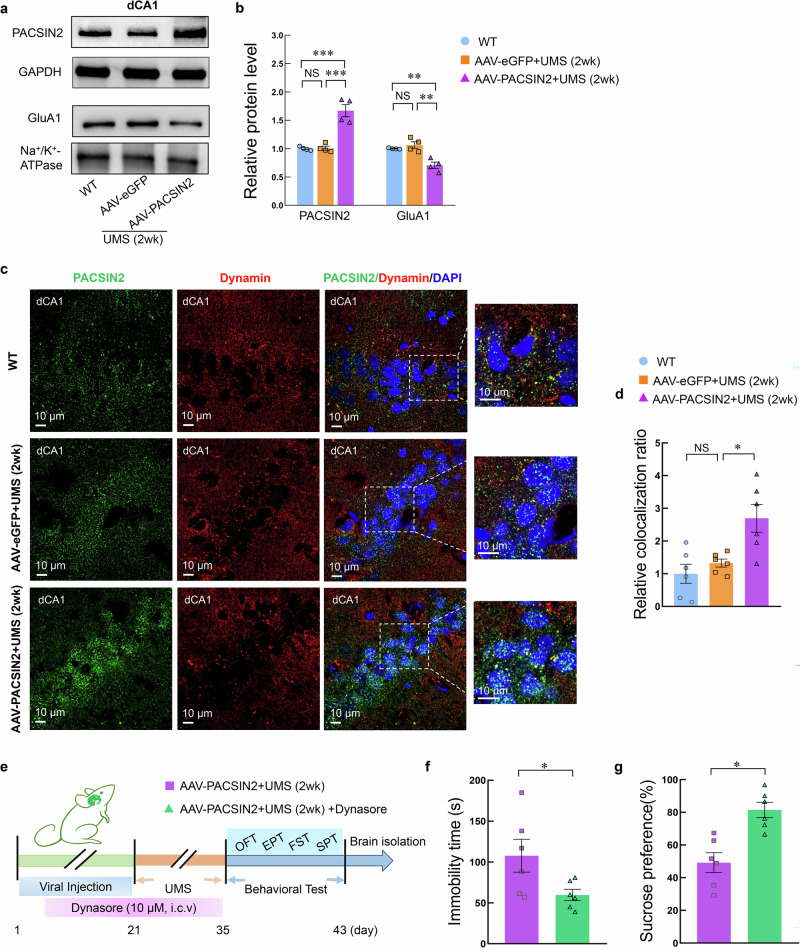
Overexpression of PACSIN2 reduces the surface GluA1 and increases colocalization of PACSIN2 and dynamin in rats. **a**, **b** Representative immunoblots and quantitative analyses of PACSIN2 and membrane GluA1 expression level in the dCA1 region of hippocampus. PACSIN2: *n*  =  4 rats per group. NS: not significant *P* > 0.05, ***P < 0.001 (One-way ANOVA with Tukey’s multiple comparisons test). Membrane GluA1: *n*  =  4 rats per group. NS: not significant *P* > 0.05, ***P < 0.001 (One-way ANOVA (nonparametric tests), Mann-Whitney U test). **c** Representative images staining of PACSIN (green), dynamin (red) and DAPI (blue) in the dCA1region of hippocampus. PACSIN2^+^/dynamin^+^ colocalizing is magnified (right). Scale bars = 10 μm. **d** Quantification of colocalization ratio between PACSIN2 and dynamin in the dCA1 region of hippocampus. *n*  = 6 slices from three rats per group. NS: not significant *P* > 0.05, *P < 0.05 (One-way ANOVA with Tukey’s multiple comparison tests). **e** Schematic diagram of experimental procedure. **f** Immobility time of rats in FST. *n*  =  6 rats each group. *P < 0.05 (Two-tailed unpaired t-test (nonparametric tests), Mann-Whitney U test). **g** The measurement of sucrose preference of rats. *n*  =  6 rats each group. *P < 0.05 (Two-tailed unpaired *t*-test). Data are represented as the mean ± SEM.

**Fig. 5:**
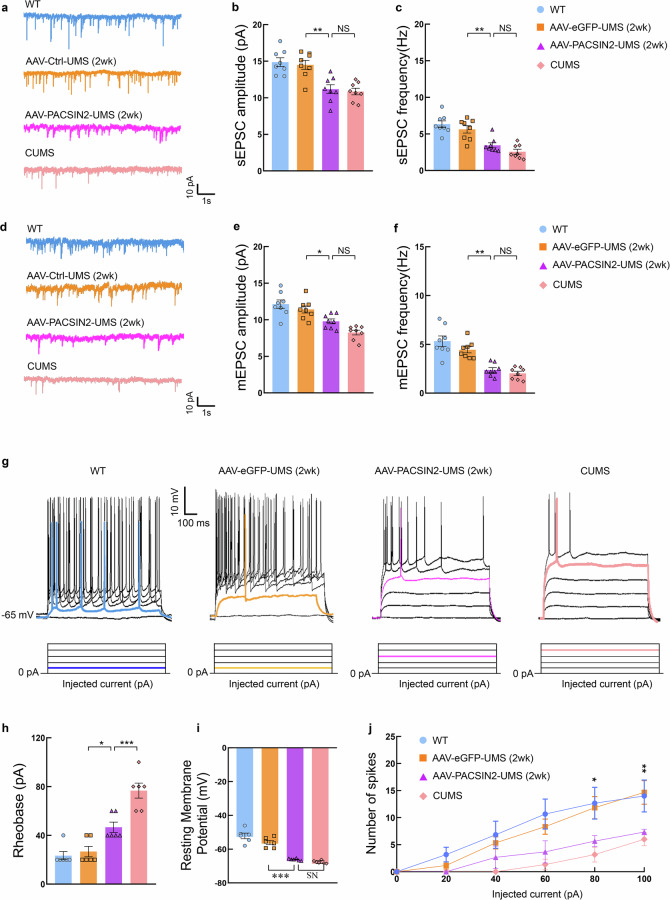
Overexpression of PACSIN2 with UMS (2wk) in CA1 hippocampal alters the synaptic function of pyramidal neurons in the CA1 hippocampal. **a** Representative sEPSCs traces in CA1 hippocampal pyramidal neurons. **b**, **c** Summary data for sEPSCs amplitude and frequency in CA1 hippocampal pyramidal neuron. *n*  =  8 neurons from four rats per group. NS: not significant *P* > 0.05, **P < 0.01 (One-way ANOVA with Tukey’s multiple comparisons test). **d** Representative mEPSCs traces in CA1 hippocampal pyramidal neurons. **e**, **f** Summary data for mEPSCs amplitude and frequency in CA1 hippocampal pyramidal neuron. *n*  =  8 neurons from four rats per group. NS: not significant *P* > 0.05, **P < 0.01 (One-way ANOVA with Tukey’s multiple comparisons test). **g** Example traces of membrane potential responses to step current injections in CA1 hippocampal pyramidal neurons. Scale, 10 mV; 100 ms. **h** Quantification of the rheobase (pA) in CA1 hippocampal pyramidal neurons. *n*  = 6 neurons from three rats per group. NS: not significant *P* > 0.05, **P < 0.01 (One-way ANOVA with Tukey’s multiple comparisons test). **i** Quantification of the RMP (mV) in CA1 hippocampal pyramidal neurons. *n*  = 6 neurons from three rats per group. ***P < 0.001 (One-way ANOVA with Tukey’s multiple comparisons test). **j** Quantification of AP numbers of CA1 hippocampal pyramidal neurons. *n*  =  6 neurons from three rats respectively. **P < 0.01, ***P < 0.001 (Two-way repeated measures ANOVA with Tukey’s multiple comparisons test). Data are represented as the mean ± SEM.

We then further investigated whether increased PACSIN2 within the dCA1 region of the hippocampus may be involved in the pathogenesis of depression (Fig. 3f). Unexpectedly, PACSIN2 overexpression did not induce depression-like behaviors (Fig. 3g–i, Supplementary Fig. 9a–f). Interestingly, only subthreshold exposure to UMS (2 weeks) was sufficient to cause significant depression-like phenotypes in these AAV-PACSIN2 rats (Fig. 3j–l, Supplementary Fig. 10a–f). These results indicate that elevated PACSIN2 levels in the dCA1 region of the hippocampus promote susceptibility to chronic stress in rats. The results revealed that the surface GluA1 levels in the membrane fraction significantly decreased after PACSIN2 overexpression with subthreshold UMS exposure (Fig. 4a, b) and the colocalization of PACSIN2 with dynamin increased in the hippocampal dCA1 region (Fig. 4c, d). Similarly, the results from electrophysiological recordings revealed that after overexpression of PACSIN2, subthreshold UMS exposure significantly decreased the frequency and amplitude of sEPSCs (Fig. 5a–c) and mEPSCs (Fig. 5d–f) and the intrinsic excitability (Fig. 5g–j) of hippocampal pyramidal neurons. Finally, we used dynasore, a specific inhibitor of dynamin, to further verify whether preventing endocytosis is responsible for the induction of depression-like behaviors. We found that inhibition of dynamin expression significantly decreased the increased susceptibility resulting from PACSIN2 overexpression combined with 2 weeks of UMS exposure (Fig. 4e–g and Supplementary Fig. 10g–l).

### PACSIN2 knockdown in the CA1 hippocampus rescues behavioral disorders in CUMS rats

**Fig. 6:**
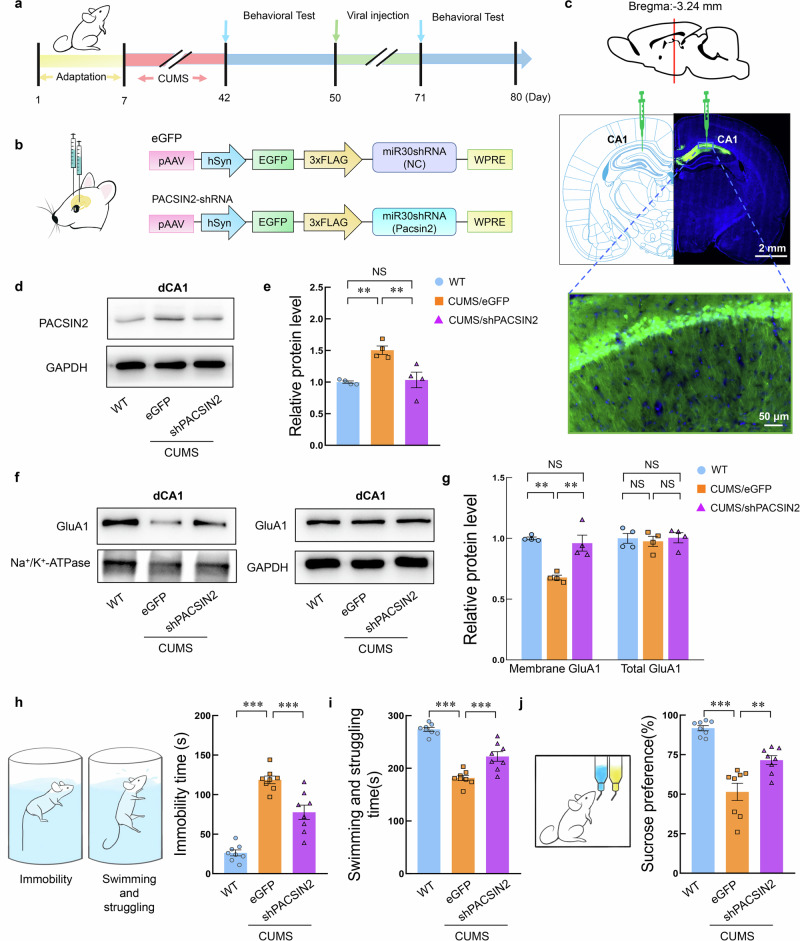
Knockdown of PACSIN2 in CA1 hippocampus rescues behavioral deficits in CUMS rats. **a** Experimental design of studies. **b**, **c** Schematics of AAV vectors and bilateral injection sites in the hippocampus. Scale bar, 2 mm. (top) and 50 μm (bottom). **d-e** Representative immunoblots and quantitative analyses of PACSIN2. *n*  =  4 rats per group. ***P < 0.001 (One-way ANOVA with Tukey’s multiple comparison tests). **f**, **g** Representative immunoblots and quantitative analyses of membrane GluA1 and total GluA1 expression levels in CA1 region of hippocampus. *n*  =  4 rats per group. NS: not significant, *P* > 0.05, **P < 0.01 (One-way ANOVA with Tukey’s multiple comparison tests). **h** Immobility time of rats in FST. WT: *n*  =  8 rats; CUMS/eGFP: *n* = 8 rats; CUMS/shPACSIN2 groups: *n* = 8 rats. ***P < 0.001 (One-way ANOVA with Tukey’s multiple comparison tests). **i** Swimming and struggling time in FST. WT: *n*  =  8 rats; CUMS/eGFP: *n*  =  8 rats; CUMS/shPACSIN2 groups: *n* = 8 rats. **P < 0.01, ***P < 0.001 (One-way ANOVA with Tukey’s multiple comparison tests). **j** The measurement of sucrose preference of rats. WT: *n*  =  8 rats; CUMS/eGFP: *n*  =  8 rats; CUMS/shPACSIN2 groups: *n* = 8 rats. NS: not significant *P* > 0.05, **P < 0.01, ***P < 0.001 (One-way ANOVA with Tukey’s multiple comparison tests). Data are represented as the mean ± SEM.

Given that CUMS exposure upregulated PACSIN2 expression, we next explored whether knockdown of PACSIN2 would alleviate depressive symptoms in this CUMS rat model. Therefore, the pAAV-hSyn-EGFP-shRNA PACSIN2 virus was injected bilaterally into the dCA1 region of the hippocampus of CUMS rats to achieve local knockdown of PACSIN2 in hippocampal neurons (Fig. 6a–e). Coimmunoprecipitation verified that compared with CUMS/eGFP, PACSIN2 knockdown in the CA1 region of the hippocampus in CUMS rats decreased the degree of interaction between PACSIN2 and dynamin (Supplementary Fig. 11a–d). The colocalization of PACSIN2 with dynamin was also markedly reduced in these PACSIN2 knockdown CUMS rats (Supplementary Fig. 12a, b). With the knockdown of PACSIN2 in CUMS rats, surface GluA1 levels in the membrane fraction significantly increased, with no differences observed in the total amount of GluA1 in the dCA1 region of the hippocampus (Fig. 6f, g). Moreover, PACSIN2 knockdown significantly ameliorated depression-like behaviors in CUMS rats (Fig. 6h–j). However, PACSIN2 knockdown did not significantly affect the duration spent in the center zone of the OFT (Supplementary Fig. 12c–e) or the number of entries into the EPM (Supplementary Fig. 12f–h). These results indicate that specific PACSIN2 downregulation could alleviate stress-induced depression-like behaviors, possibly by reducing the PACSIN2-dynamin interaction, which in turn decreases GluA1 endocytosis.

### PACSIN2 knockdown in the CA1 region of the hippocampus reverses synaptic plasticity impairments

**Fig. 7:**
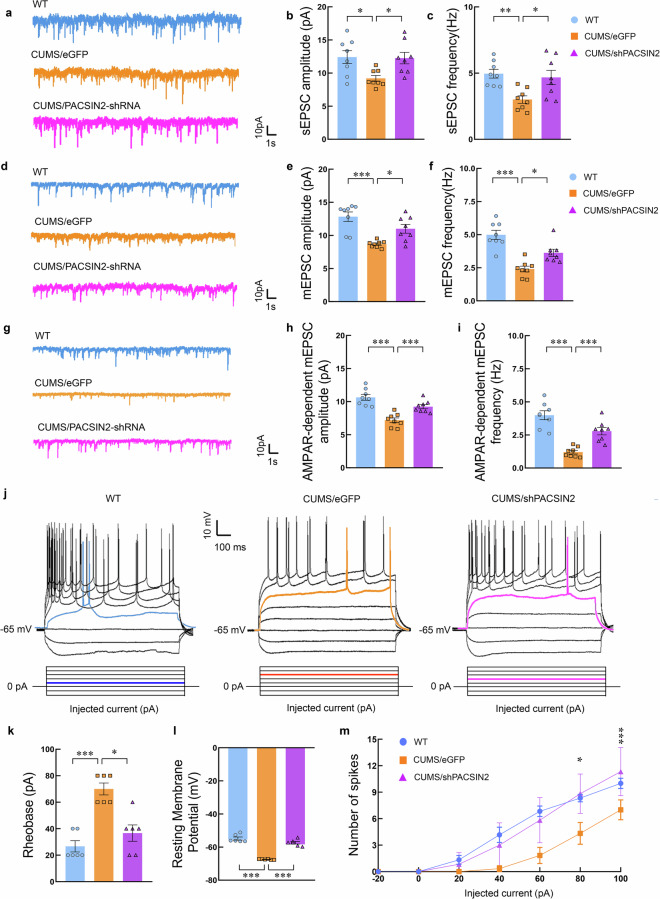
PACSIN2 knockdown in the CA1 hippocampus restores synaptic transmission. **a** Representative sEPSCs traces in CA1 hippocampal pyramidal neurons of rats. **b**, **c** Summary data for sEPSCs amplitude and frequency in pyramidal neurons. *n*  =  8 neurons from four rats for each group. *P < 0.05, **P < 0.01 (One-way ANOVA with Tukey’s multiple comparison tests). **d** Representative mEPSCs traces in CA1 hippocampal pyramidal neurons of rats. **e**, **f** Summary data for mEPSCs amplitude and frequency in pyramidal neurons. *n*  =  8 neurons from four rats for each group. *P < 0.05, **P < 0.01 (One-way ANOVA with Tukey’s multiple comparison tests). **g** Representative traces for AMPAR-mediated mEPSCs recorded at CA1 hippocampal pyramidal neurons. Summary data for AMPAR-mediated mEPSCs amplitude (**h**) and frequency (**i**) in CA1 hippocampal pyramidal neurons of rats. *n*  =  8 neurons from four rats per group. ***P < 0.001 (One-way ANOVA with Tukey’s multiple comparisons test). **j** Example traces of membrane potential responses to step current injections in CA1 hippocampal pyramidal neurons. Scale, 10 mV; 100 ms. **k** Quantification of the rheobase (pA) in CA1 hippocampal pyramidal neurons. *n*  = 6 neurons from three rats per group. NS: not significant *P* > 0.05, **P < 0.01 (One-way ANOVA with Tukey’s multiple comparisons test). **l** Quantification of the RMP (mV) in CA1 hippocampal pyramidal neurons. *n*  = 6 neurons from three rats per group. ***P < 0.001 (One-way ANOVA with Tukey’s multiple comparisons test). **m** Quantification of AP numbers of CA1 hippocampal pyramidal neurons. *n*  =  6 neurons, respectively, from three animals in each group. *P < 0.05, ***P < 0.001 (Two-way repeated measures ANOVA with Tukey’s multiple comparisons test). Data are represented as the mean ± SEM.

Whole-cell patch-clamp recordings of pyramidal cells revealed that knockdown of PACSIN2 in CUMS rats resulted in significant increases in the frequency and amplitude of sEPSCs (Fig. 7a–c) and mEPSCs (Fig. 7d–f). In addition, both the amplitude and the frequency of AMPAR-dependent mEPSCs increased (Fig. 7g–i). Spike thresholds and RMP were significantly lower (Fig. 7j–l), and the number of evoked APs increased (Fig. 7m) in brain sections from the PACSIN2 downregulation group than in those from the eGFP group after the CUMS procedure when identical injection currents were tested. Taken together, these data indicate that knockdown of PACSIN2 in the dCA1 hippocampal region increases the excitability of CA1 pyramidal neurons and synaptic transmission in CUMS rats.

### Pharmacological activation of AMPARs ameliorates CUMS-induced depression-like behaviors

**Fig. 8:**
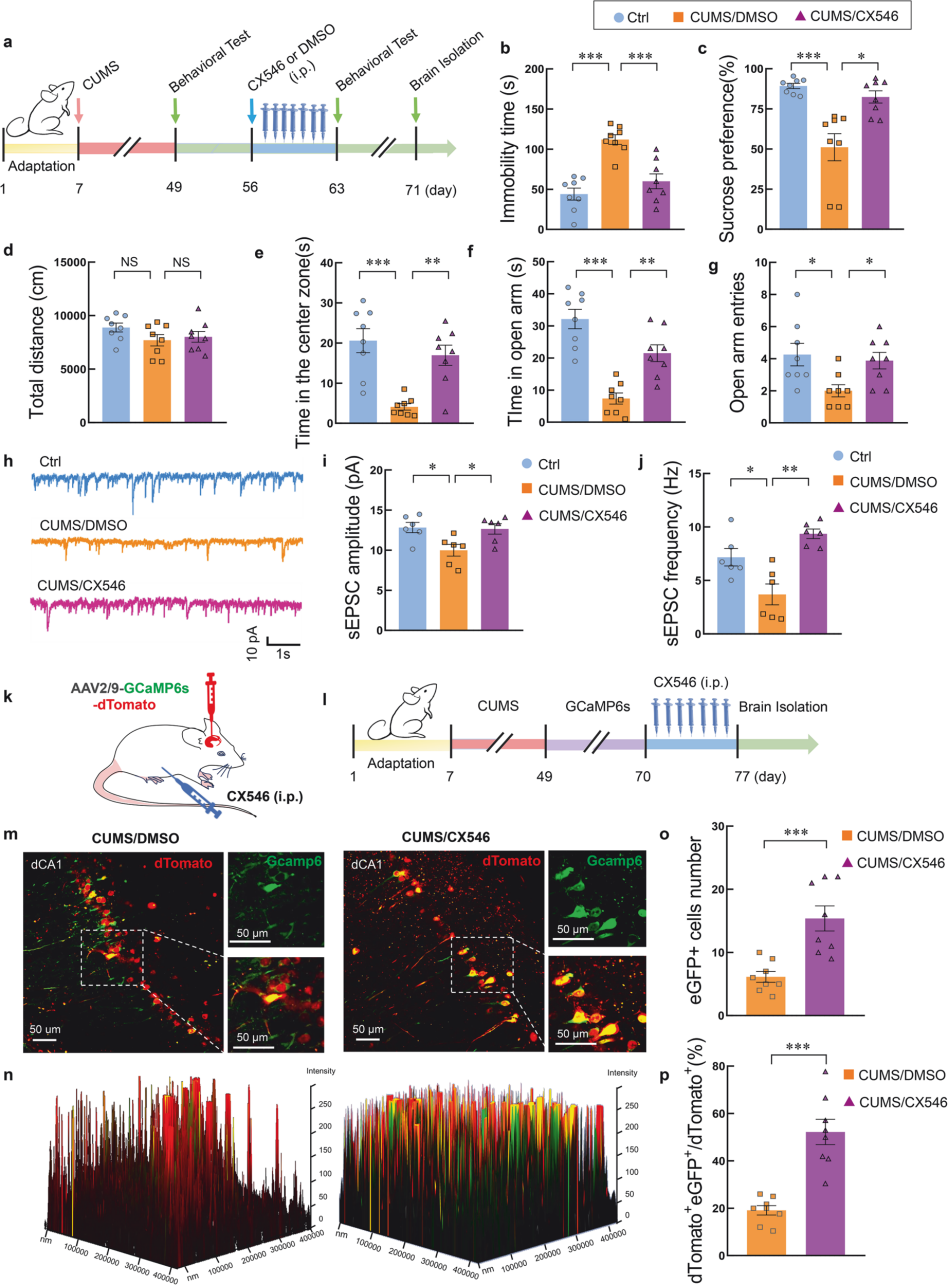
Pharmacological activation of AMPARs ameliorates stress-induced depression-like behaviors. **a** Experimental design for studies. **b** Immobility time of rats in FST. *n*  =  8 rats per group. ***P < 0.001 (One-way ANOVA with Tukey’s multiple comparison tests). **c** The measurement of sucrose preference of rats. Ctrl: *n*  =  8 rats; CUMS/DMSO: *n*  =  8 rats; CUMS/CX546 groups: *n* = 8 rats. **P < 0.01 (One-way ANOVA with Tukey’s multiple comparison tests). **d**, **e** The total distance and the center time of rats in OFT. *n*  =  8 rats per group. NS: not significant *P* > 0.05, **P < 0.01 (One-way ANOVA with Tukey’s multiple comparison tests). **f**, **g** The open arm time and entries of rats in EPM. *n*  =  8 rats per group. NS: not significant, *P* > 0.05, ***P < 0.001 (One-way ANOVA (nonparametric tests). **h** Representative sEPSCs traces in CA1 hippocampal pyramidal neurons (Scale, 10 pA, 1 s). **i**, **j** Summary data for sEPSCs amplitude and frequency in CA1 hippocampal pyramidal neurons. Ctrl: *n*  =  6 neurons from three rats; CUMS/DMSO: *n*  =  6 neurons from three rats for each group; CUMS/CX546: *n*  = 6 neurons from three rats for each group). *P* > 0.05, **P < 0.01 (One-way ANOVA with Tukey’s multiple comparison tests). **k**, **l** Schematic of viral injection and experimental design. **m** Representative original and magnified images showing Ca^2+^ levels indicated by AAV-dTomato-Gcamp6-EGFP virus in rats. Scale bars, 50 μm. **n** Representative images of 2.5 D rebuilt the picture through ZEN blue software. **o** Bar graphs show quantification of the number of eGFP^+^ cells in the hippocampus of rats. CUMS/DMSO: *n* = 8 slices from three rats; CUMS/CX546: *n* = 8 slices from three rats. ***P < 0.001 (Two-tailed unpaired *t*-test). **p** Bar graphs show the percent of dTomato^+^/eGFP^+^ cells in the hippocampus. CUMS/DMSO: *n* = 8 slices from three rats; CUMS/CX546: *n* = 8 slices from three rats. ***P < 0.001 (Two-tailed unpaired *t*-test). Data are represented as the mean ± SEM.

Given that defects in AMPAR insertion in postsynaptic membranes represent a major basis for the hypoactivity of neurons in CUMS rats, we were interested in determining whether enhancing AMPAR activity could alleviate the depression phenotypes of CUMS rats. Notably, AMPAkines have been investigated as possible treatments for depression [43], schizophrenia [44], Alzheimer’s disease [45] and Huntington’s disease [46, 47]. Accordingly, we investigated whether CX546, an established AMPAkine, could reverse the behavioral disorders induced by CUMS exposure. CX546 (20 mg/kg) treatment significantly reversed the depression-like behaviors resulting from CUMS (Fig. 8b, c). While CX546 treatment failed to alter the amount of locomotion in the OFT (Fig. 8d), it did significantly reverse the anxiety-like behavioral responses (center time) associated with this test (Fig. 8e). CX546 treatment also increased the amount of time spent in and number of entries into the open arms of the EPM test (Fig. 8f, g). The results of electrophysiological recordings revealed that the decreased amplitudes and frequencies (Fig. 8h–j) of sEPSCs resulting from CUMS exposure were reversed by CX546 injection. To visualize the changes in the excitability of pyramidal neurons within the dCA1 region after CX546 treatment, CUMS rats received bilateral injections of AAV2/9-GCaMP6s-dTomato virus into the dCA1 region of the hippocampus (Fig. 8k, l). The fluorescence images obtained were assessed with the use of a 2.5D image rebuilt by ZEN blue software. The results revealed that the number of eGFP^+^ cells and dTomato^+^/eGFP^+^ cells significantly increased in CUMS rats treated with CX546 (Fig. 8m–p). These results corroborate the notion that CX546 treatment significantly ameliorated CUMS-induced depression-like and anxiety-like behaviors by increasing synaptic transmission.

## Discussion

In the present study, we demonstrated that chronic stress upregulates PACSIN2 levels in the dCA1 region of the hippocampus. Such an effect promotes the endocytosis of the GluA1 subunits of AMPA receptors and impairs the structure and function of synapses, eventually leading to depression-like behaviors. In response to overexpression of PACSIN2 in the dCA1 hippocampal region, normal rats are more susceptible to chronic stress, whereas knockdown of PACSIN2 rescues behavioral disorders resulting from CUMS exposure. Mechanistically, we showed that the increased binding of PACSIN2 to dynamin reduces the surface expression of GluA1 AMPA receptors in membrane fractions and subsequently decreases synaptic transmission. When dynasore, an antagonist of dynamin, was administered, PACSIN2-mediated depression-like behaviors were abolished. Furthermore, CX546, an AMPA receptor agonist, improved synaptic transmission and ameliorated chronic stress-induced depression-like behaviors in rats. Collectively, these findings document a pivotal role for PACSIN2 in the pathogenesis of depression and raise the possibility of developing antidepressants that can selectively target PACSIN2.

Emerging evidence suggests that MDD is a consequence of contributions from both genetic differences and environmental factors, such as chronic stress [48–50]. Depression, which is induced by chronic stress, appears to result in impairments in synaptic structure and function [51, 52]. Notably, the hippocampus, particularly the dCA1 region, has recently been implicated in emotional and cognitive regulation [53–55]. Dendritic spines are the postsynaptic components of most excitatory synapses in the mammalian brain [56, 57]. We found that upon exposure to chronic stress, the expression of presynaptic Bassoon and GluA1 subunits decreased in the hippocampal area. Moreover, CUMS decreased the frequency and amplitude of sEPSCs and mEPSCs and the frequency of APs and increased the minimal threshold of injected currents needed to induce APs. Accordingly, these data indicate that CUMS exposure led to maladaptation in the structure and function of synapses. However, the underlying mechanisms of these pathophysiological processes related to depression are not fully understood.

A proper excitatory/inhibitory (E/I) balance is critical for normal adult brain function [58, 59]. Therefore, we further investigated the relationship between changes in the excitability of hippocampal neurons and depression-like behaviors. The results showed that inhibition of hippocampal CA1 pyramidal neurons can induce depression-like behavior in animals. In short, these data suggest that the hypoactivity of excitatory pyramidal neurons contributes to the depressive-like behaviors observed in CUMS rats.

PACSIN2 (also termed syndapin 2) is a member of the F-BAR domain protein subfamily, which includes three paralogs and consists of an F-BAR and an SH3 domain [60, 61]. PACSIN2 is a well-known regulator of the actin cytoskeleton and endocytosis and plays a role in intracellular vesicle-mediated transport [31, 62]. Furthermore, findings from epidemiological and genomic studies have indicated that PACSIN2 plays a role in the organization of the cytoskeletal architecture, which is believed to be important for the development of neurodegenerative disorders [63–67]. In addition, DEG analysis and WGCNA were conducted to identify the genes related to PTSD and MDD, and the PACSIN2 gene was identified [66]. Therefore, we hypothesized that PACSIN2 may be involved in the occurrence of chronic stress-induced depression-like behaviors by regulating the endocytosis of neuronal postsynaptic receptors. Here, proteomic analysis revealed that the protein expression of PACSIN2 was significantly increased in the dCA1 region of the hippocampus of CUMS rats, which was consistent with previous findings obtained in a mouse model of depression [65]. Furthermore, there may be a direct interaction between PACSIN2 and dynamin. Dynamin, the founding member of a family of dynamin-like proteins (DLPs), has been implicated in membrane remodeling and plays a critical role in endocytic membrane fission events [68, 69]. In this study, we revealed that chronic stress did not affect the expression of dynamin. Interestingly, the results of our IP analysis further indicated that CUMS increased the interaction between PACSIN2 and dynamin, which may thus promote the endocytosis and internalization of membrane receptors. In the nervous system, different subtypes of dynamin are expressed at the pre- and postsynaptic levels, and these subtypes participate in several processes involved in modulating synaptic transmission [70, 71]. Notably, dynamins regulate the surface availability of neurotransmitter receptors by tuning receptor turnover at the postsynaptic level [72, 73]. As synaptic plasticity responses mostly rely on the density, type and properties of postsynaptic receptors, the abundance of AMPARs at the synapse is crucial for synaptic efficiency and communication between neurons [74]. Therefore, AMPAR trafficking into or out of the synaptic membrane is a major regulatory mechanism underlying modifications in the efficacy of excitatory synaptic transmission [75]. Accumulating evidence has indicated that dysregulation of AMPAR dynamic neuronal trafficking may be critical for the treatment of neuropsychiatric and neurodegenerative diseases [26, 76]. In this study, we revealed that CUMS decreased surface GluA1 subunit levels in the dCA1 region of the hippocampus, whereas total GluA1 levels did not significantly change. The electrophysiological data also revealed that CUMS significantly reduced the amplitude and frequency of AMPAR-dependent mEPSCs. More importantly, we found that overexpression of PACSIN2 increased the susceptibility to stress in animals, and these effects were accompanied by decreases in surface GluA1 subunits in the excitatory synapses of primary cultured neurons. Therefore, the above evidence suggests that PACSIN2 is a key molecular pathological feature that closely regulates synaptic AMPAR endocytosis via its binding to dynamin.

In an attempt to assess whether increased levels of PACSIN2 in the hippocampal dCA1 region can induce depression phenotypes, we found that PACSIN2 overexpression did not directly induce depression-like behaviors in rats. However, in rats overexpressing PACSIN2, depression-like behaviors could be induced by subthreshold exposure to UMS, an effect that was blocked by the administration of a dynamin antagonist. Simultaneously, PACSIN2 upregulation in hippocampal pyramidal neurons resulted in decreased frequencies and amplitudes of mEPSCs and decreased firing rates, suggesting that the excitatory synaptic transmission and intrinsic excitability of the neurons clearly decreased. Notably, dynasore, a highly specific antagonist of the GTPase activity of dynamin [77], significantly reversed PACSIN2-induced depression-like behaviors. Dynasore has also been shown to rescue pilocarpine-induced seizures by inhibiting dynamin activity [36]. Accordingly, these findings reveal that PACSIN2 may increase individual susceptibility to external stress.

Previous studies have indicated that PACSIN2 comprises a Fes-CIP4 homology Bin-Amphiphysin-Rvs161/167 (F-BAR) domain in the N-terminal region, an SH3 domain in the C-terminal region and a variable central region that contains NPF motifs. The SH3 domain and NPF motifs mediate the interaction of PACSIN2 with a repertoire of proteins involved in endocytosis, regulation of the cytoskeleton, signaling and receptor trafficking, such as Neuronal Wiskot–Aldrich Syndrome Protein (n-wasp) and Ras-related C3 botulinum toxin substrate 1 (Rac1) [29]. Thus, we hypothesize that upregulated PACSIN2 expression may enhance its binding not only to dynamin but also to other potential interacting proteins that may also facilitate the endocytosis process, but this hypothesis needs to be further verified. Coimmunoprecipitation (Co-IP) experiments were performed using high-affinity dynamin-specific antibodies, and immunoblot results confirmed the specificity of the PACSIN2-dynamin interaction detected in our study. Our results showed that CUMS enhanced PACSIN2-dynamin binding, whereas specific PACSIN2 knockdown in the hippocampal neurons of CUMS rats reduced this interaction. These findings indicate that other molecules may not influence the PACSIN2-dynamin binding interaction. In addition, our findings indicate that chronic stress may upregulate the expression of PACSIN2 in the hippocampal CA1 region and increase its binding to dynamin, thereby promoting the endocytosis of GluA1 and reducing the expression of GluA1 on the surface of the postsynaptic membrane, further leading to damage to AMPAR-mediated synaptic transmission, which is consistent with previous reports that a 20%–30% change in AMPA receptor trafficking is sufficient to impair synaptic plasticity [78]. This change impairs the integration and transmission function of hippocampal neuronal network signals, ultimately leading to the occurrence of depressive-like behaviors. As depression involves multiple etiological mechanisms along with changes in many other genes or proteins [8, 79], PACSIN2 may be just one of many neuronal regulators contributing to the chronic induction of impairments in neurosynaptic plasticity. Therefore, the modest PACSIN2-dynamin interaction does not act in isolation but rather synergizes with other molecules.

Given that PACSIN2 can promote the endocytosis of AMPARs and that CUMS exposure can increase the expression of PACSIN2, we considered the possibility that the downregulation of PACSIN2 could serve as an effective strategy to ameliorate depression-like behaviors. In fact, we found that PACSIN2 knockdown promoted AMPAR insertion, synaptic restoration and amelioration of depressive-like behaviors. Mechanistically, specific PACSIN2 downregulation in hippocampal pyramidal neurons led to a reduction in the PACSIN2-dynamin interaction, which may have in turn decreased GluA1 endocytosis; this decrease in endocytosis consequently increased GluA1 levels in the plasma membrane fraction and ultimately rescued impaired excitatory synaptic transmission and intrinsic neuronal excitability in these neurons. These marked antidepressant-like effects were confirmed on the basis of the results of pharmacological manipulations. Specifically, following direct activation of AMPA receptors by CX546, the impairments in excitatory synaptic transmission in the hippocampal dCA1 region and depression-like phenotypes in the CUMS-induced rat model significantly improved. However, the amount of time and number of entries into the open arms of the EPM did not change in these rats, indicating that PACSIN2 knockdown appears to be effective mainly in the recovery of depression-like symptoms but not anxiety symptoms. One possibility is that the hippocampus is not the only brain region that governs anxiety regulation, and its functions rely on functional synergy with the amygdala, prefrontal cortex, and other regions. When PACSIN2 is knocked down only in the dCA1 hippocampal region, it may fail to reverse abnormal activity in other brain regions. These results suggest that the downregulation of PACSIN2 may serve as a potential protective strategy in the treatment of neuronal impairments in CUMS rats. These conclusions are in agreement with the results of a previous study demonstrating that PACSIN2 is involved in inducing long-term depression by mediating AMPAR trafficking in Purkinje cells of the cerebellum [33].

Multiple studies have reported proteomic analyses of hippocampal and other brain tissues (prefrontal cortex) in CUMS animals. Fan et al. identified 21 hippocampal DEPs in CUMS mice, and KEGG analysis linked complement and coagulation cascades to depression pathogenesis [80]. In addition, Chen et al. highlighted actin cytoskeleton regulation, MAPK signaling, and pathogenic *Escherichia coli* infection as relevant to MDD through the use of hippocampal DEPs [81]. Sun et al. associated most DEPs with synaptogenesis, mitochondrial dysfunction, and phosphoinositide metabolism [82]. Liao et al. reported that prefrontal cortex DEPs were enriched in signaling, secretion, and synaptic dysfunctions in CMS-susceptible rats [83]. Most previous studies analyzed the whole hippocampus or other brain regions (prefrontal cortex), whereas our study specifically targeted the CA1 subregion and identified CA1-enriched DEPs related to endocytosis, glutamatergic synapses, lysosomes, and autophagy. Our study contributes to the literature by providing CA1-specific proteomic insights, which help to elucidate the brain region-specific molecular mechanisms involved in CUMS-induced depression.

The data presented above suggest that excitatory synaptic dysfunction in the hippocampus is a key phenotypic change resulting from chronic stress. The findings that the AMPAR agonist AMPAkine CX546 improves the behavior disorders observed in CUMS rats provide robust support for this conclusion. Moreover, similar results were obtained in a mouse model of autism spectrum disorder, in which the intraperitoneal administration of CX546 to these mice rescued impaired AMPAR function and ameliorated their social deficits [84, 85]. Here, we revealed that CX546 treatment significantly increased synaptic transmission in the brain slices of CA1 pyramidal neurons from CUMS rats. With the use of a calcium ion fluorescent probe, we observed that CX546 improved the excitability of CA1 pyramidal neurons. When these findings are combined, it is reasonable to conclude that enhancing AMPAR activity can rescue chronic stress-induced depression behaviors, likely through its ability to correct aberrant excitatory synaptic transmission.

As depression involves multiple etiological mechanisms along with changes in many other genes or proteins, PACSIN2 may be just one of many neuronal regulators contributing to the chronic induction of impairments in synaptic plasticity. The fact that the knockdown of PACSIN2 did not fully reverse the CUMS-induced depression-like phenotypes supports the possibility that a complexity of neural mechanisms underlie the pathogenesis of depression. Therefore, future research should explore how chronic stress alters the expression of other regulators and how these factors mediate stress-induced behavioral disorders.

However, several limitations of the current study should be noted. First, we did not provide direct evidence that PACSIN2 regulates AMPA receptor internalization; instead, the process of endocytosis was demonstrated by comparing the surface and total GluA1 of the neurons indirectly, which requires further investigation. Second, long-term i.c.v. of dynasore is nonselective, and it is necessary to further reinforce its specificity through genetic approaches. Third, because the depression model construction process is long (~5 weeks), considering the possible effects of the estrous cycle and estrogen on animal behaviors, in the present study, we focused only on male animals. Previous studies have reported that estrogen influences neurotransmitter systems and mood regulatory systems in women by interacting with life stress [86]. Therefore, the potential molecular mechanism underlying the effects of sex hormones on depression should be further investigated in future studies. Finally, some experiments involved a small number of animals, which may affect the objective analysis of the final results and should receive attention in the future.

In summary, our present results demonstrate that chronic stress-induced depression-like behaviors are associated with increased PACSIN2 levels in the dCA1 region of the hippocampus. Such effects can then promote impairments in synaptic transmission and reductions in neuronal excitability. These findings provide a new and important foundation for the development of interventions to improve the function of synapses as a potential strategy for the treatment of depression.

## Supplementary information

Supplementary Materials

Supplementary Table 1

Supplementary Figures
