# Supplementary material for: PACSIN2-mediated synaptic injury contributes to behavioral disorders caused by chronic stress
Source: Acta Pharmacol Sin. 2026 May 7;47(9):2358–76. doi: 10.1038/s41401-026-01790-0 (PMC13487163; doi:10.1038/s41401-026-01790-0)

## Figure 1 a

GluA1

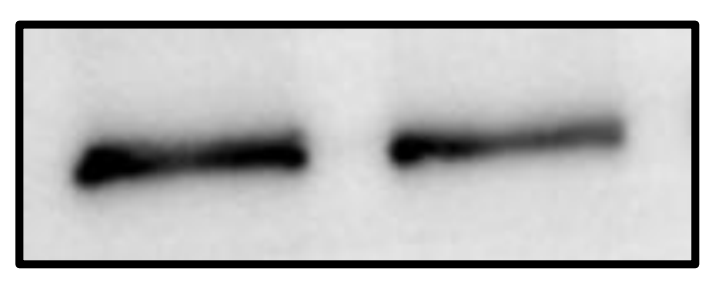

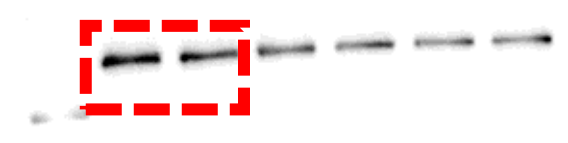

106 kDa

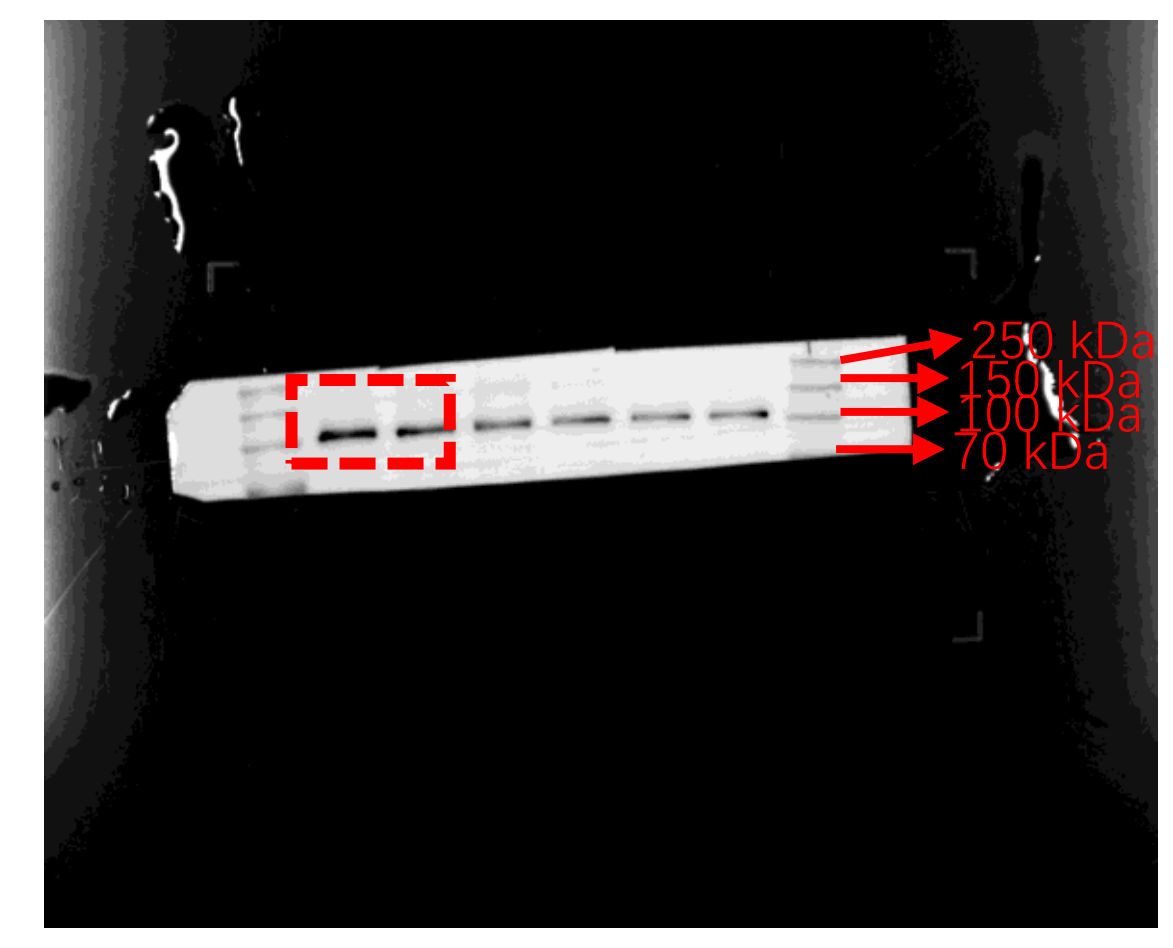

Na⁺/K⁺-ATPase

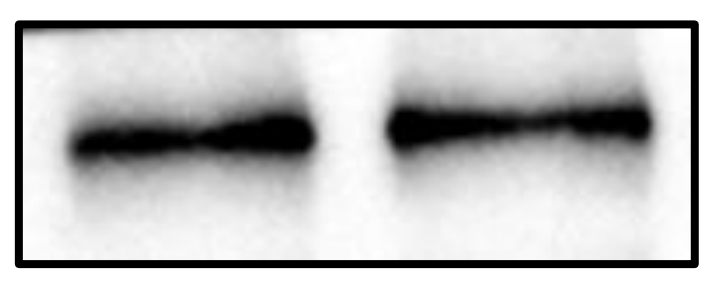

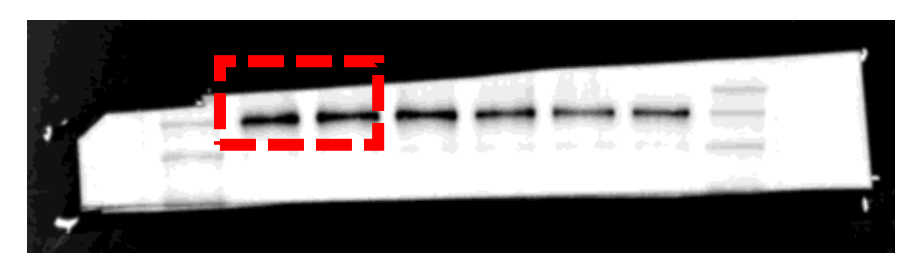

110 kDa

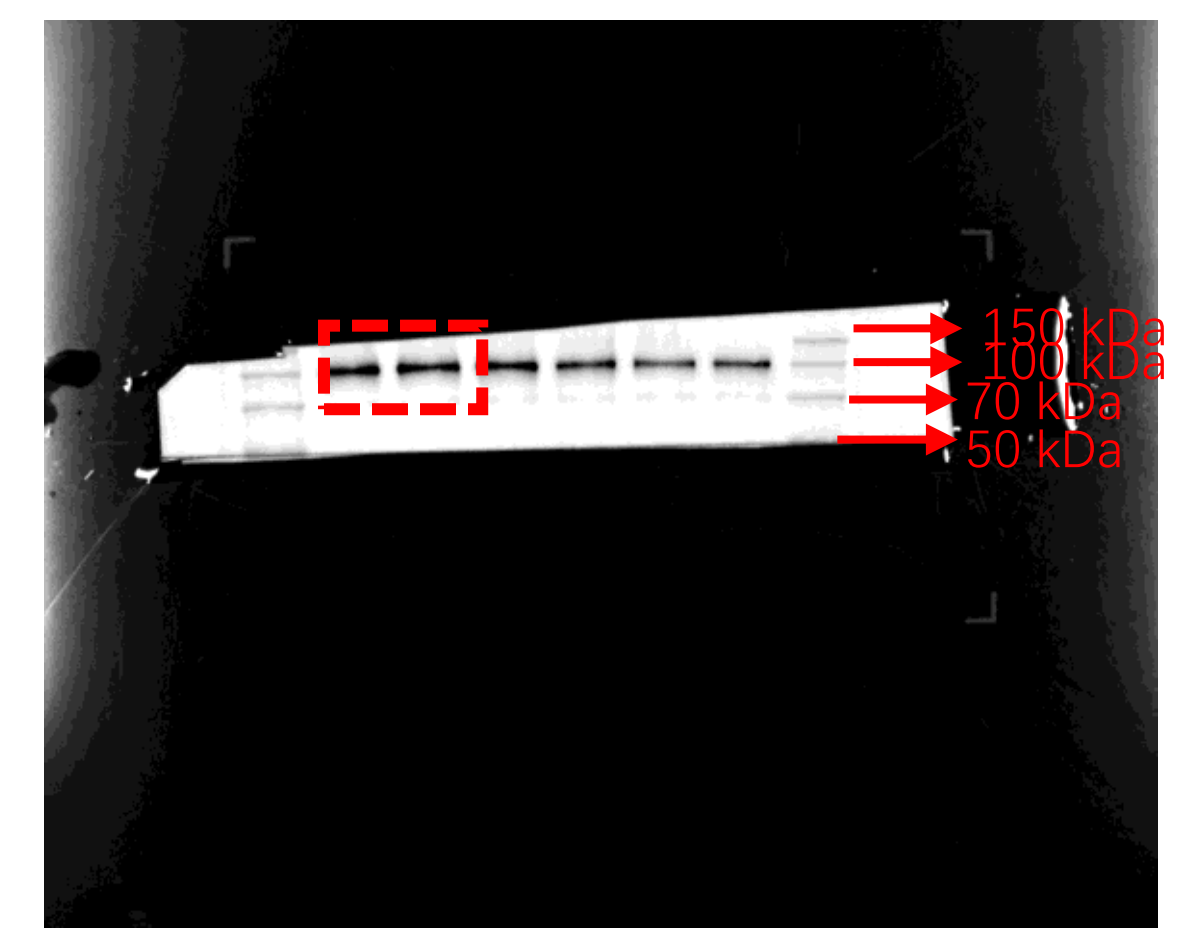

GluA2

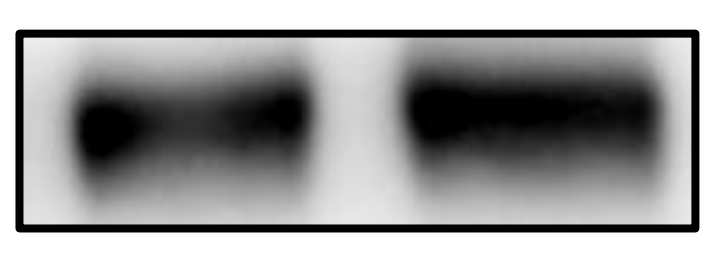

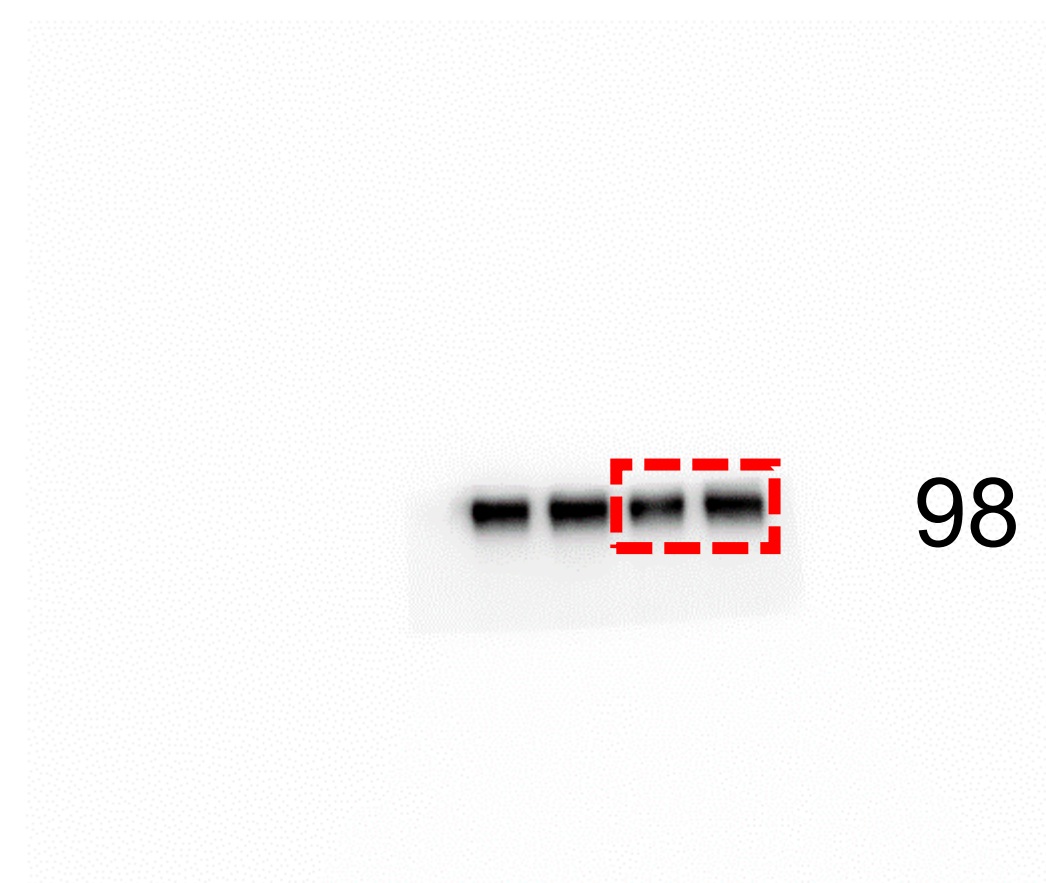

98 kDa

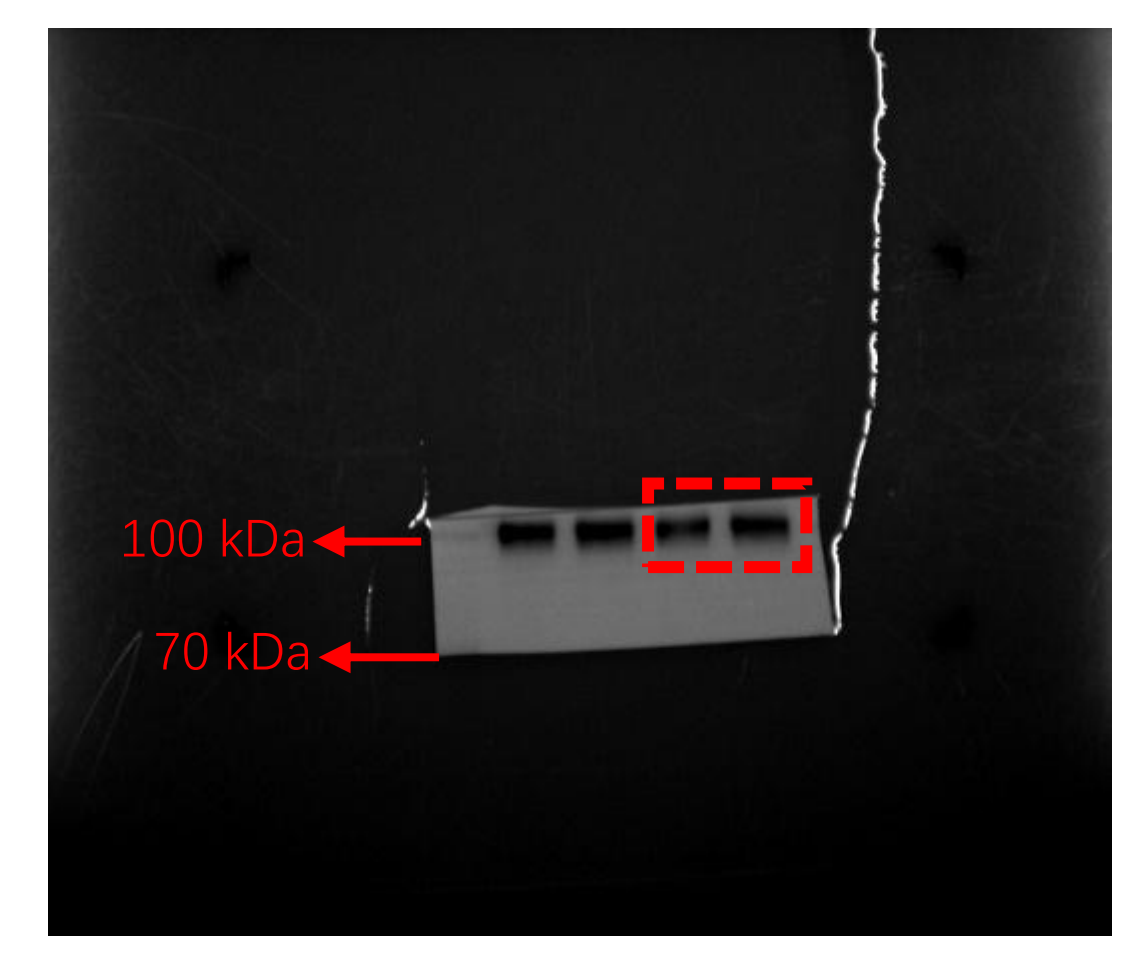


GluN2A

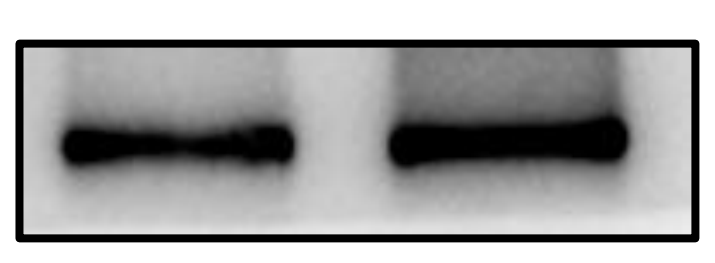

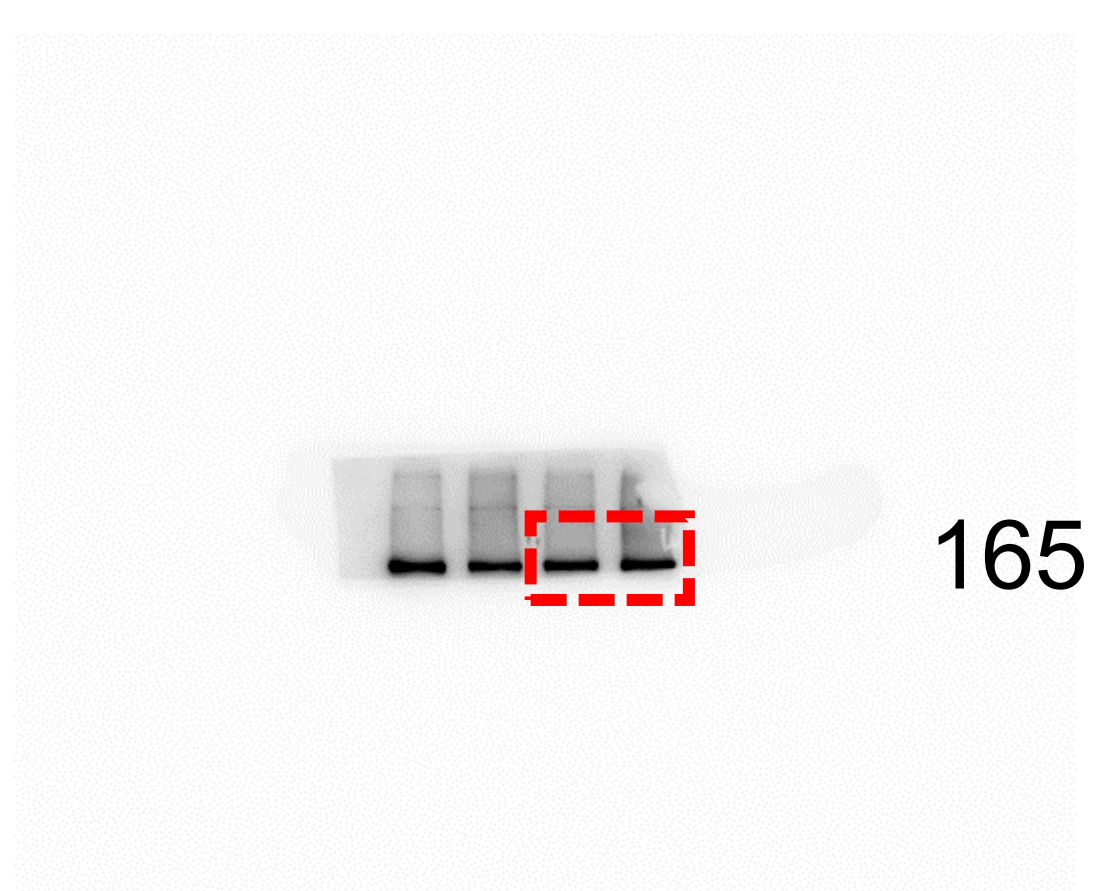

165 kDa

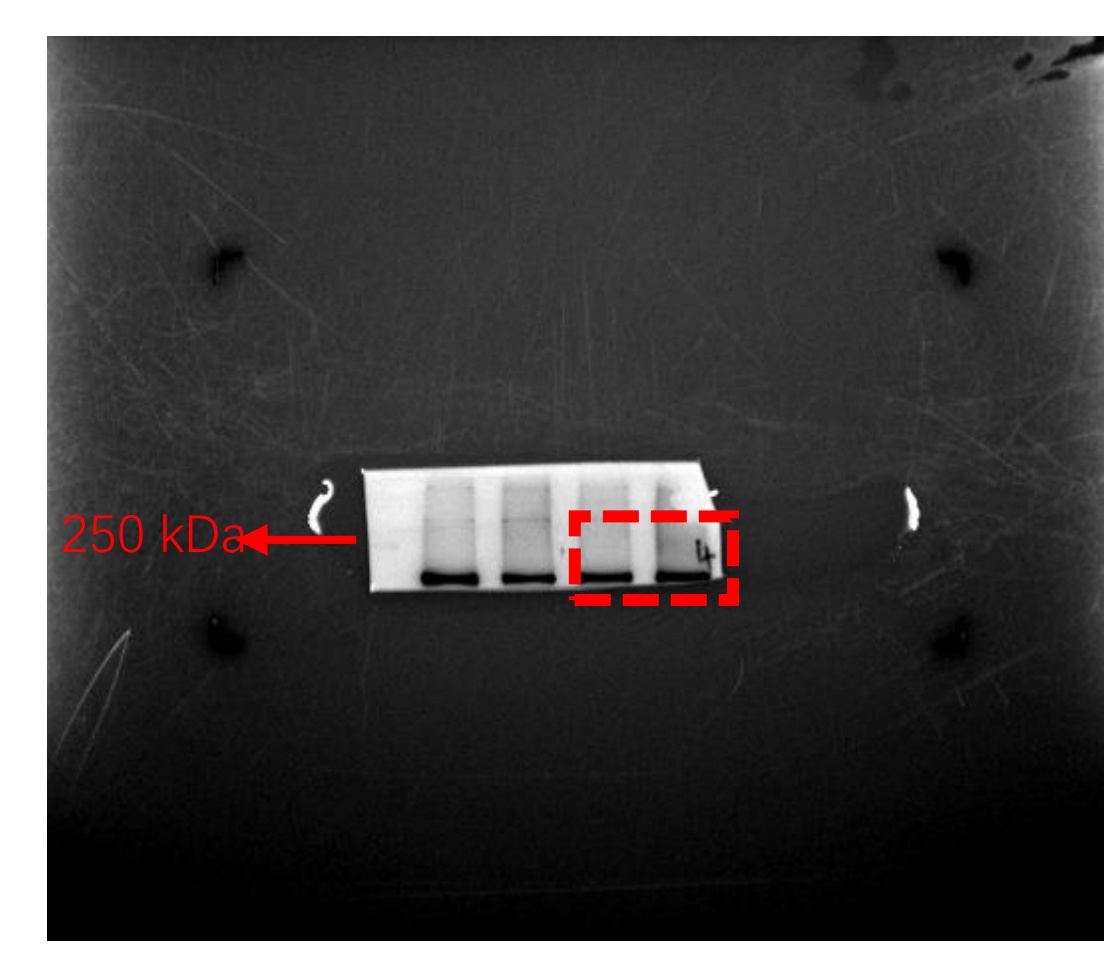

Na⁺/K⁺-ATPase

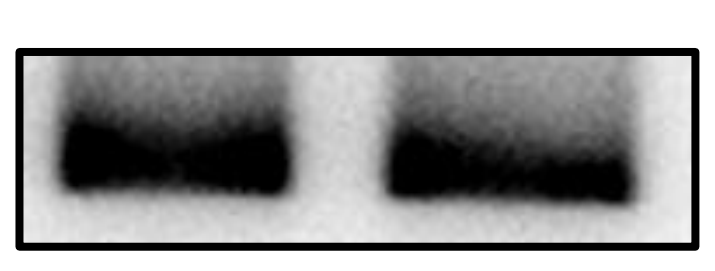

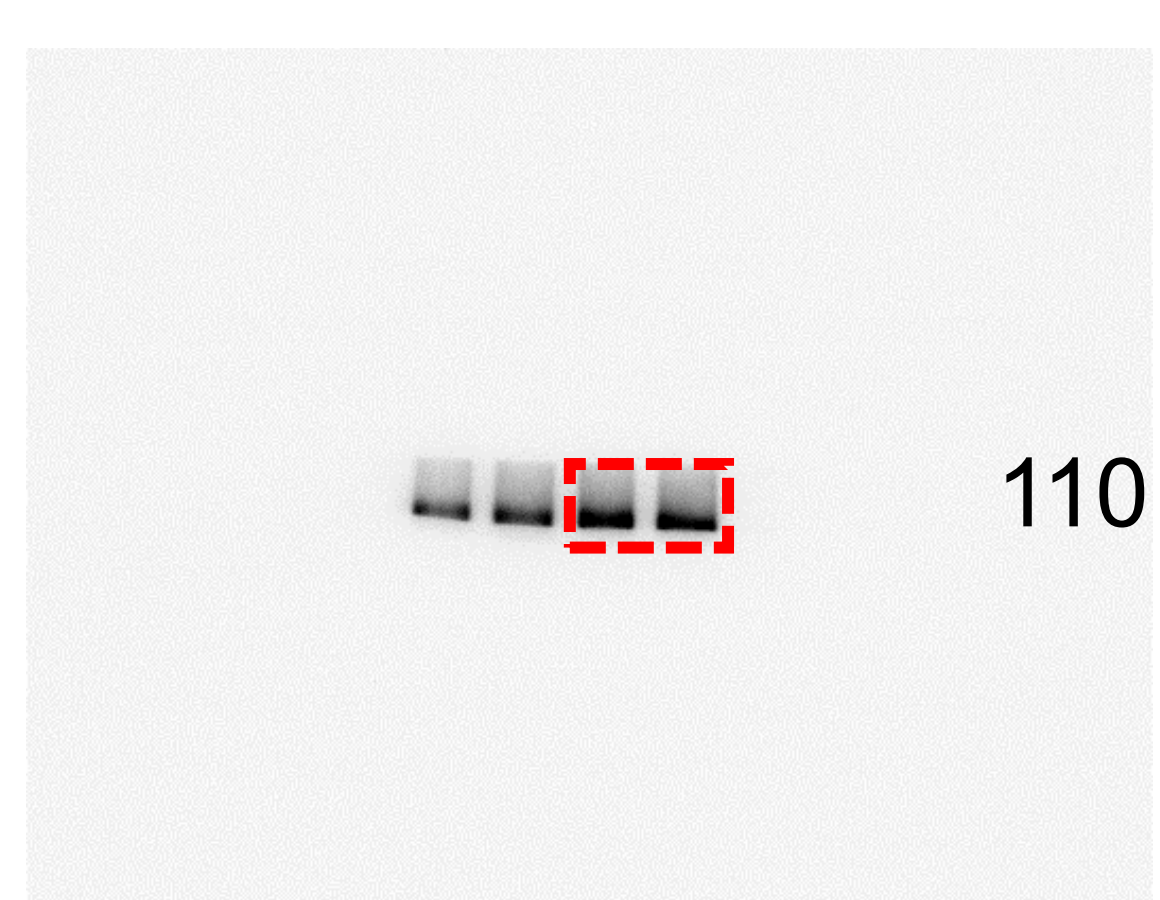

110 kDa

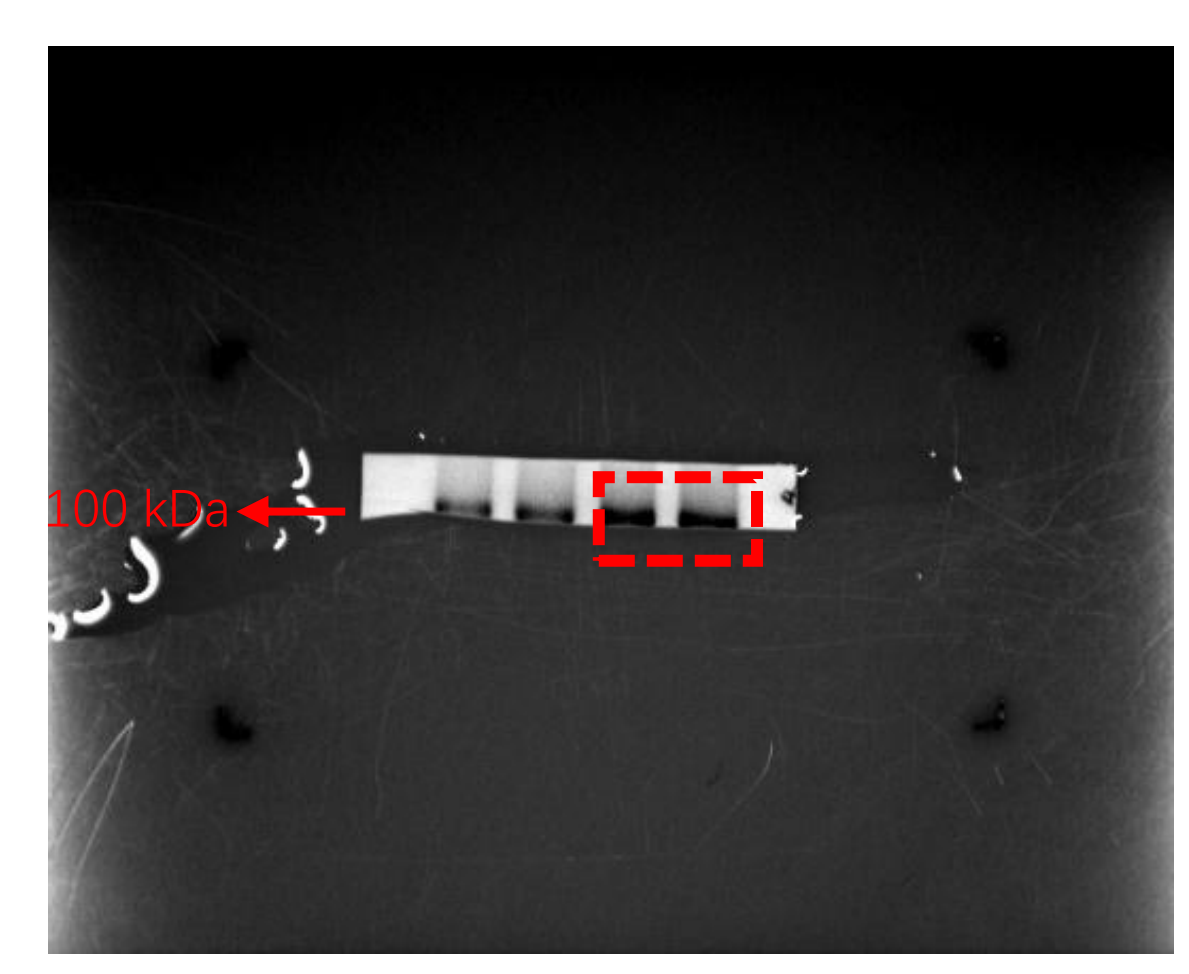

GluN1

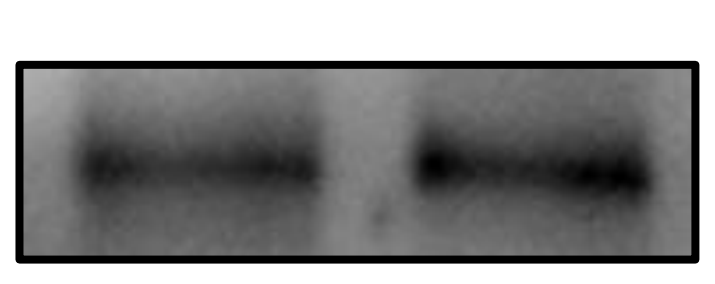

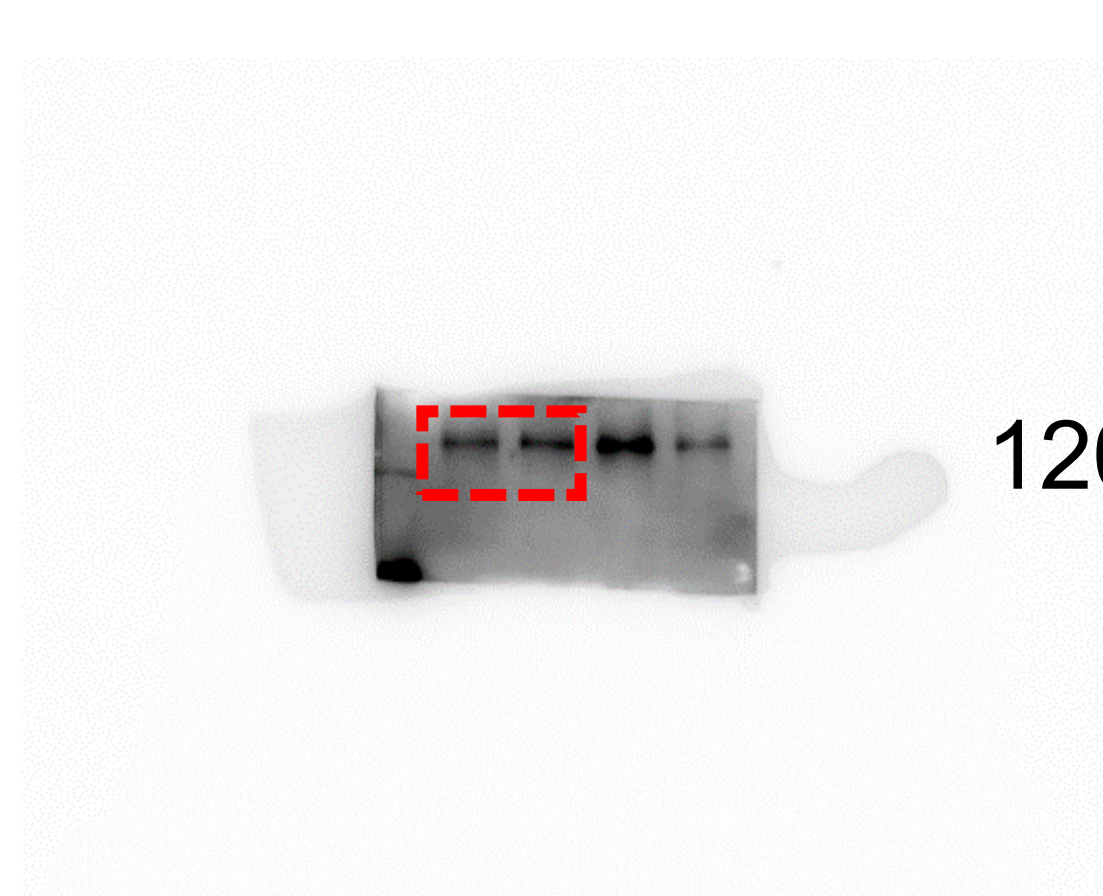

120 kDa

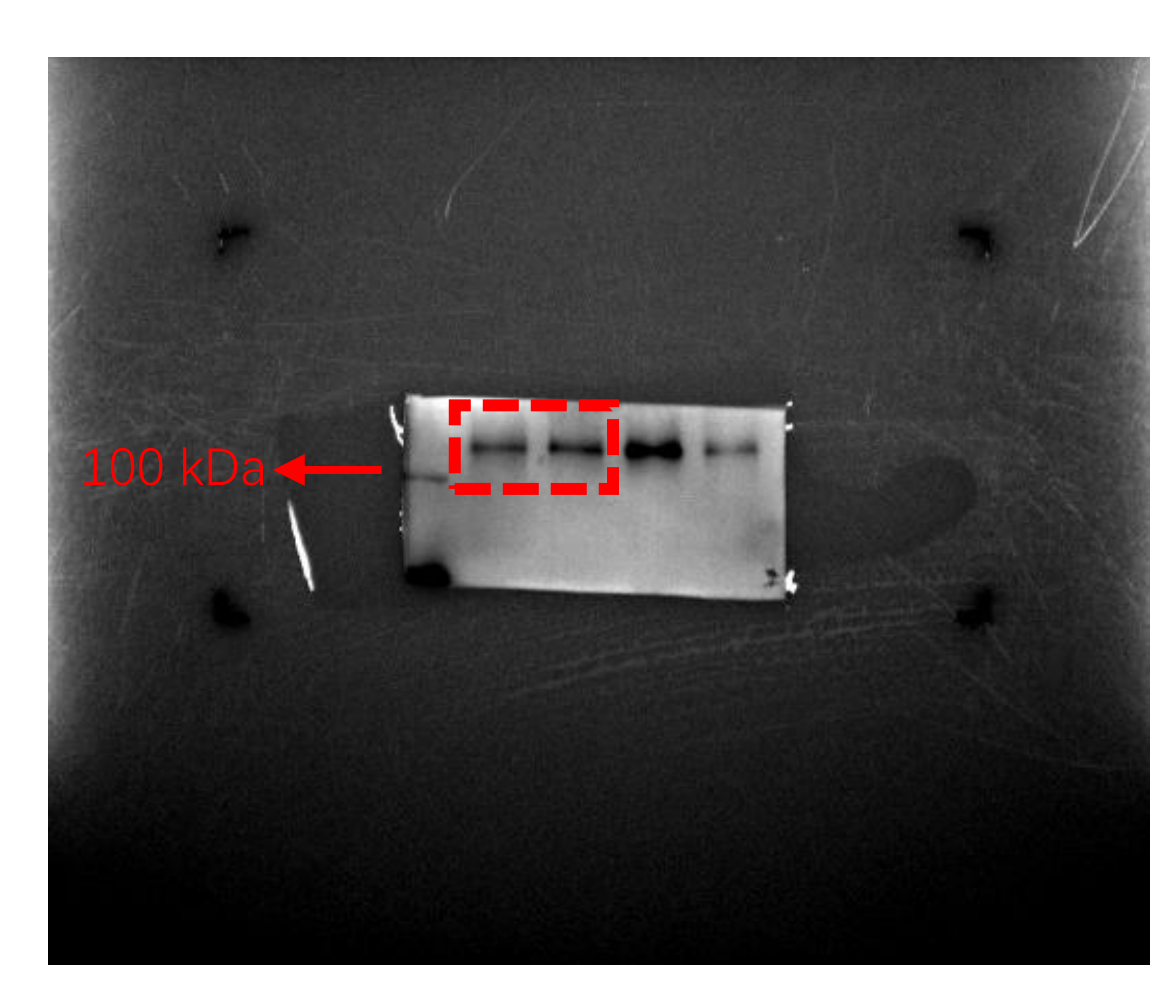

Na⁺/K⁺-ATPase

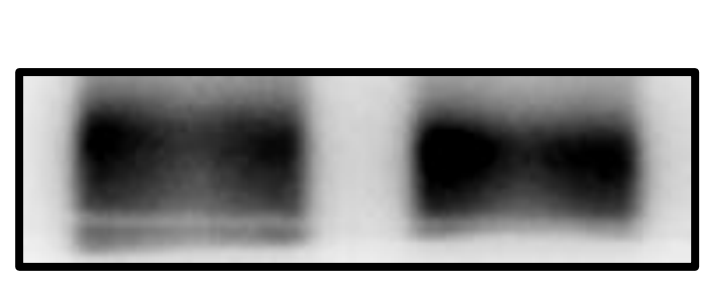

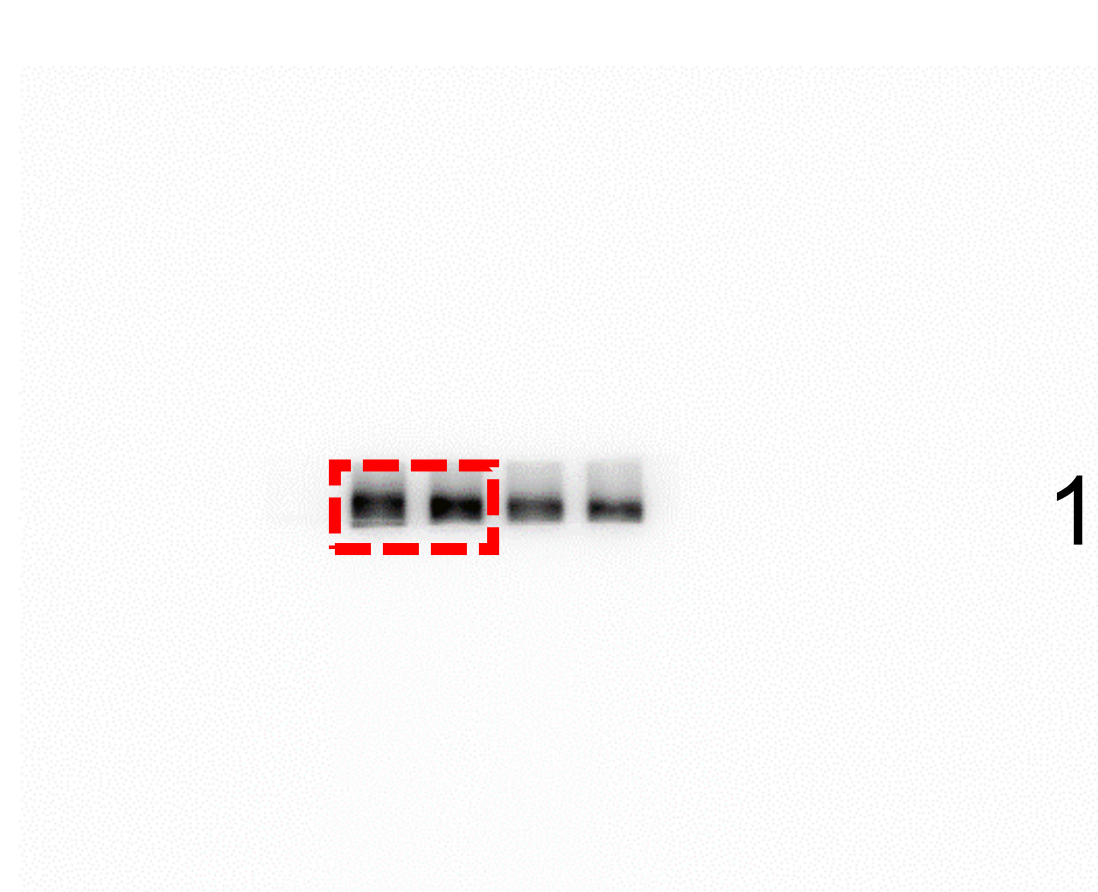

110 kDa

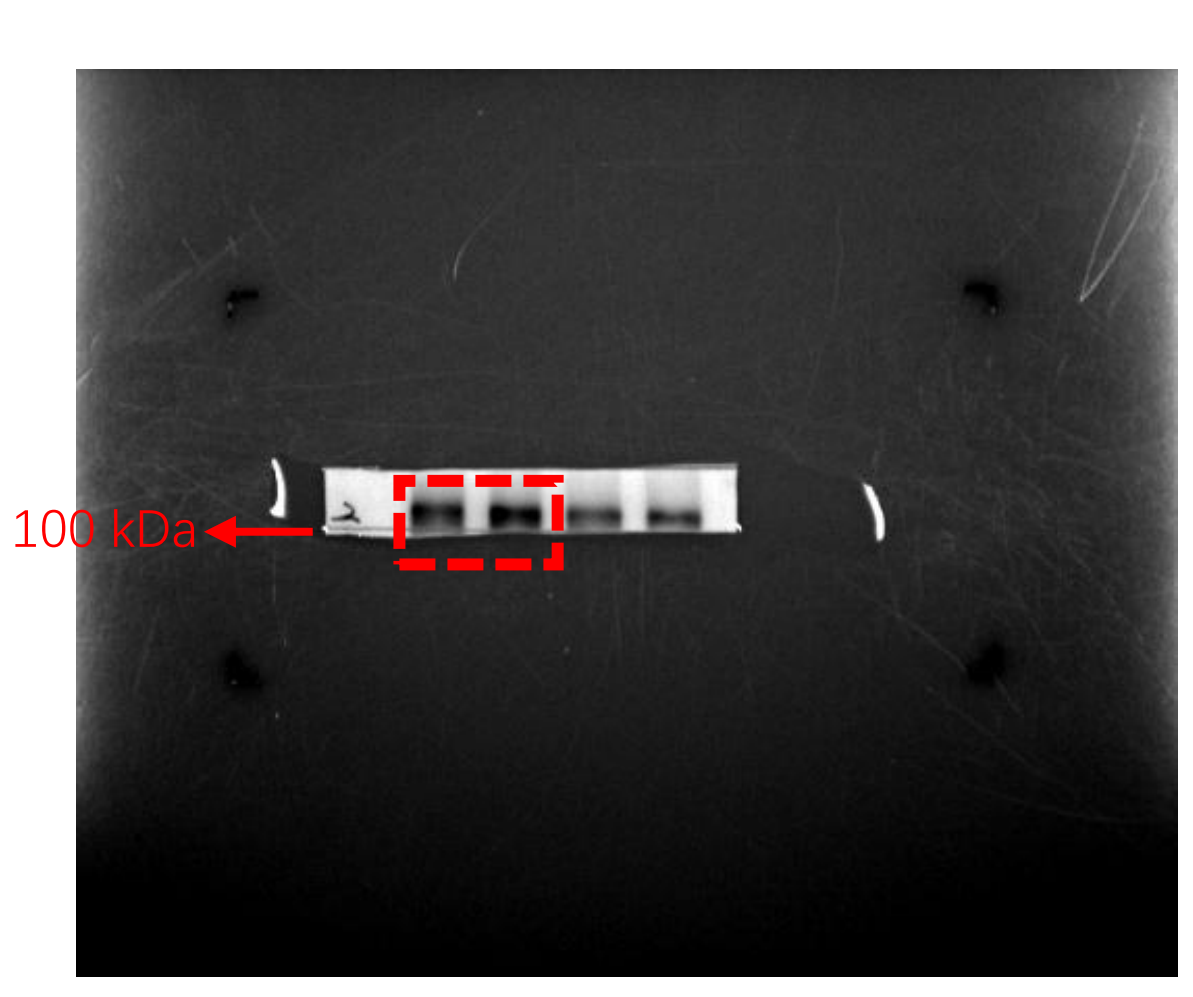

## Figure 1 c

GluA1

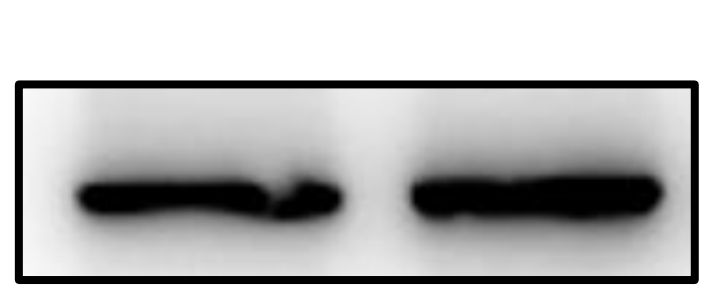

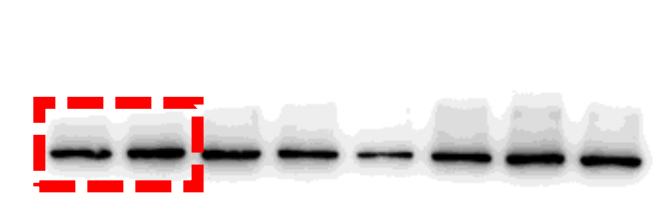

106 kDa

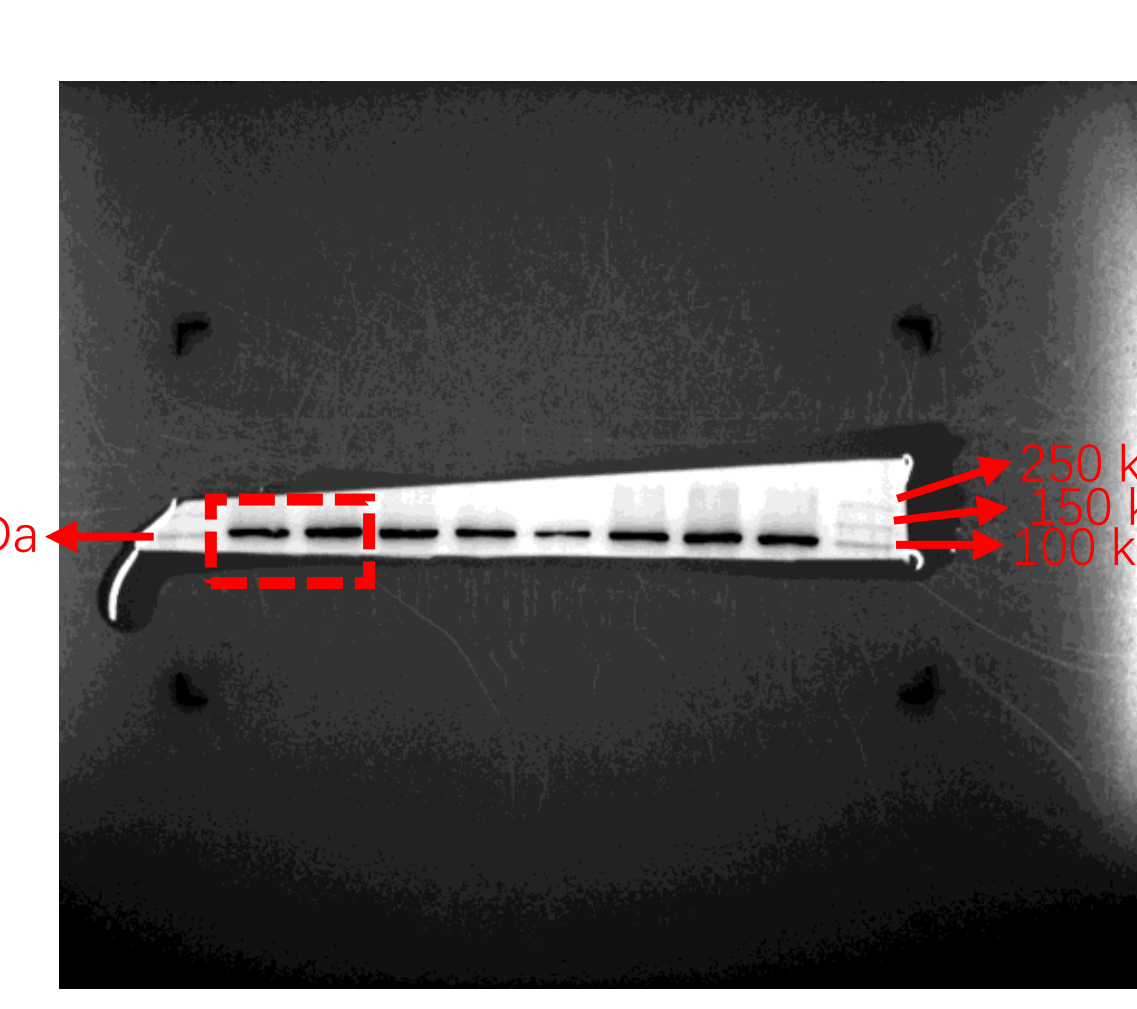


GAPDH

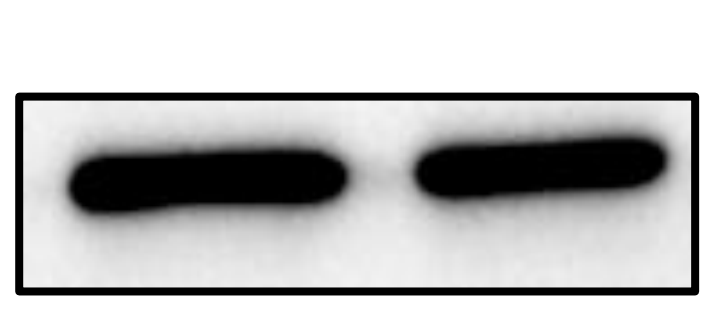

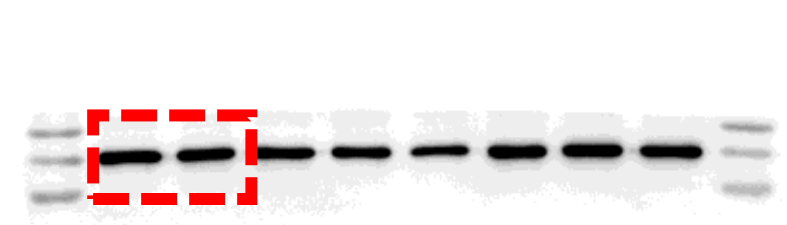

36 kDa

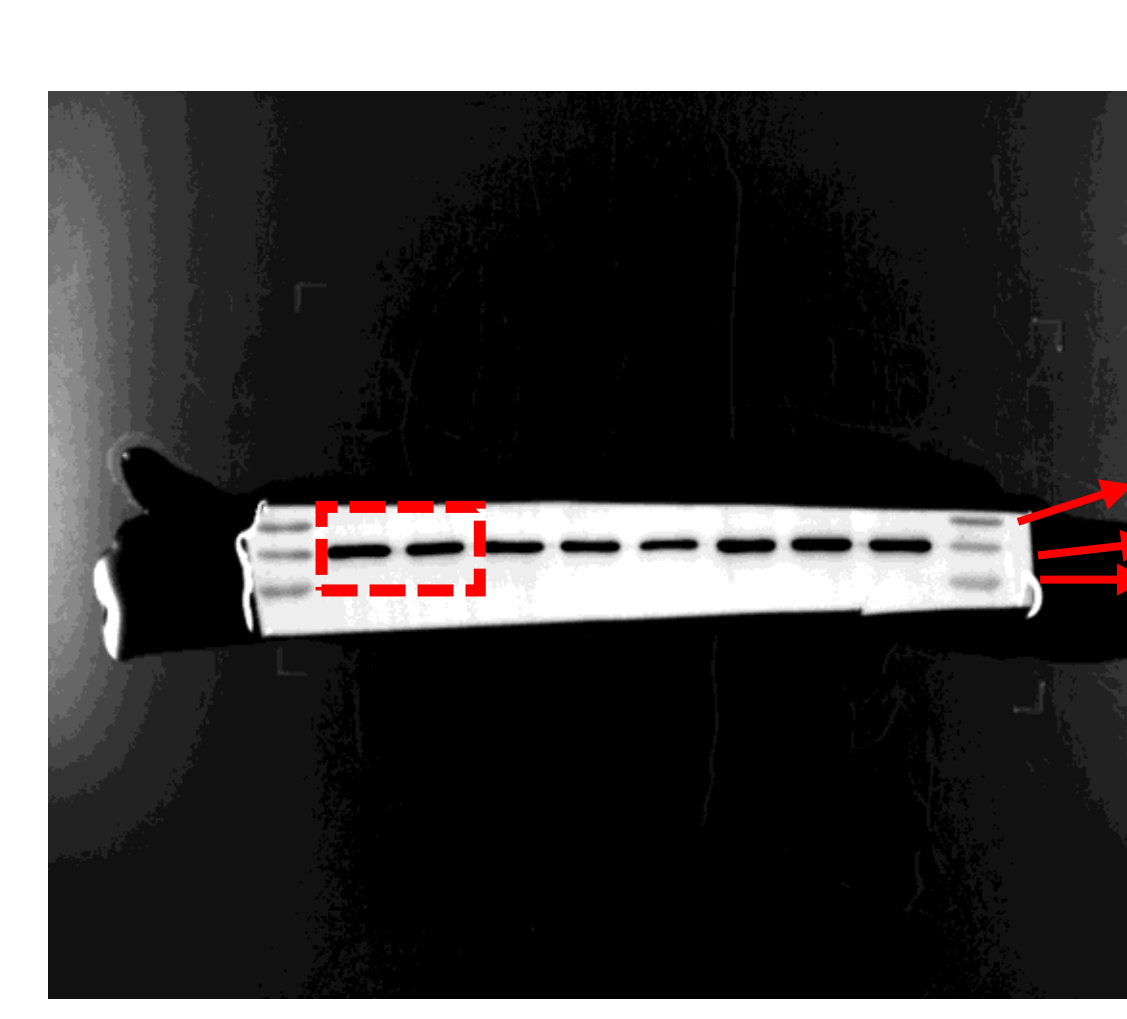

Figure 2f

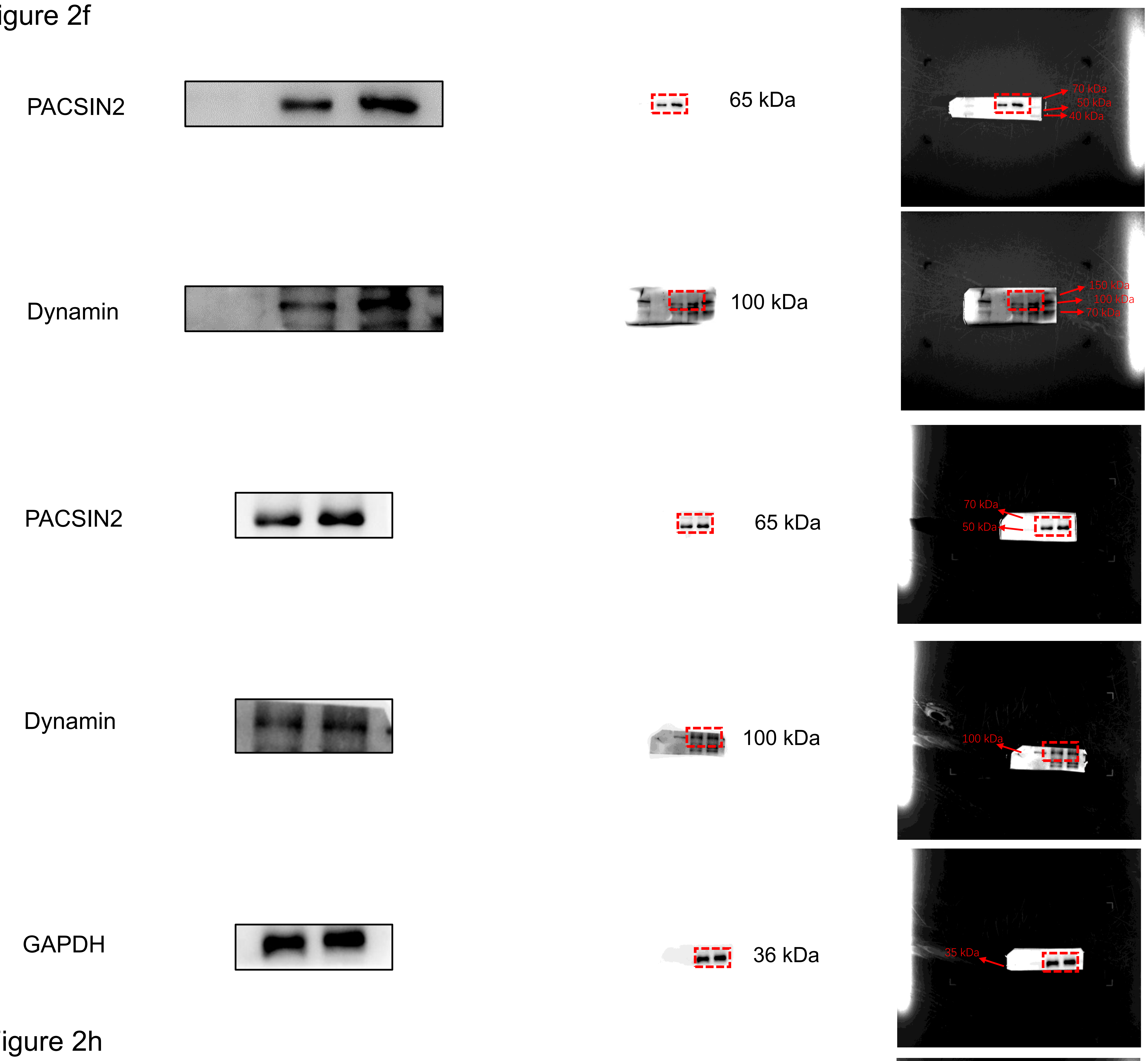


Figure 2h

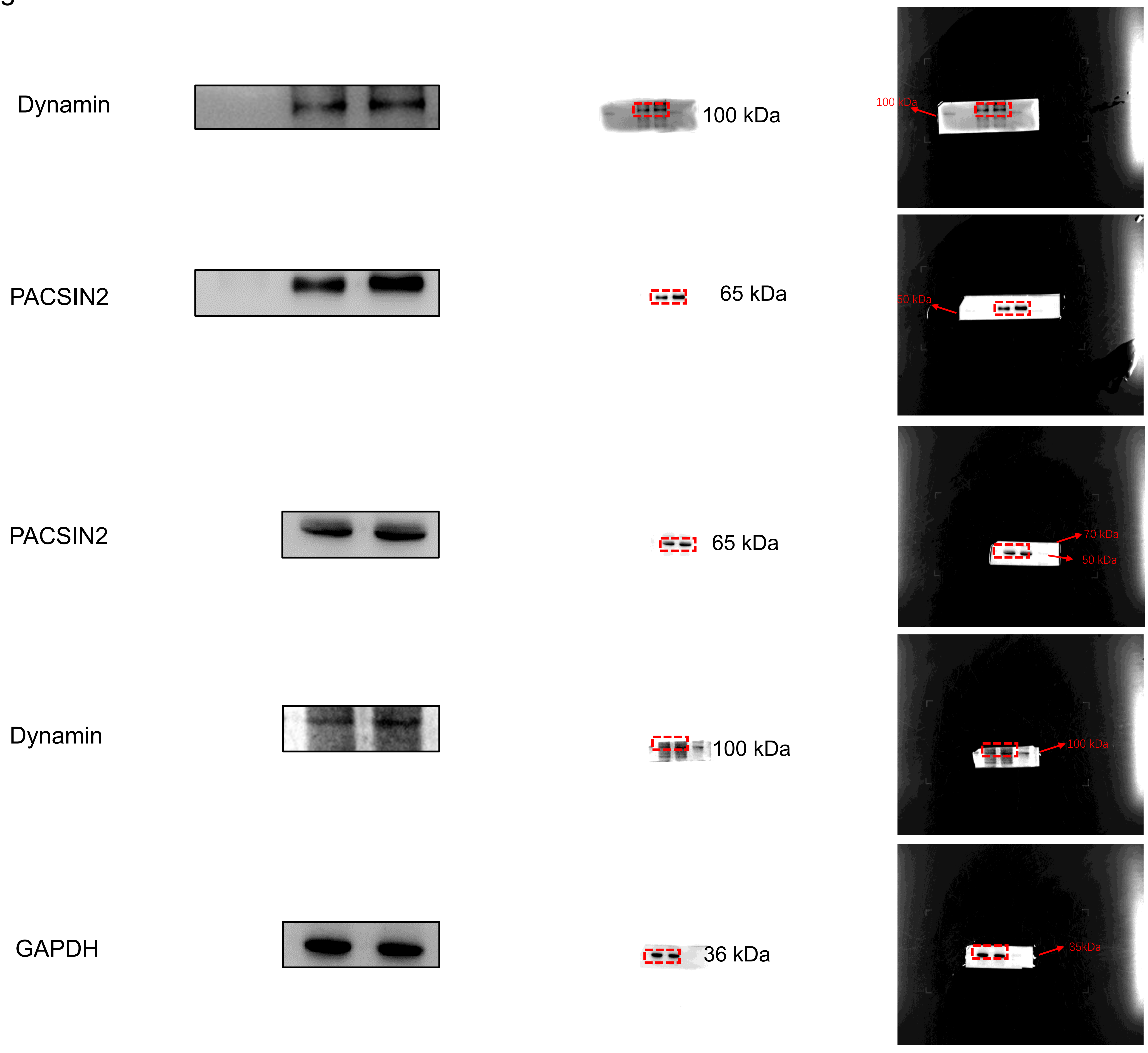

Figure 2j

PACSIN2

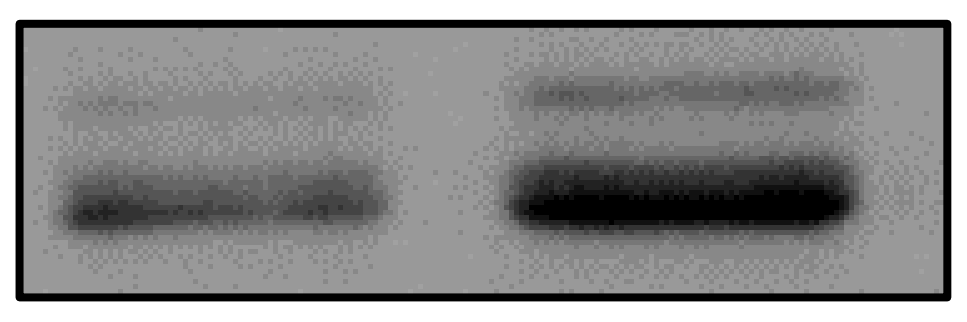

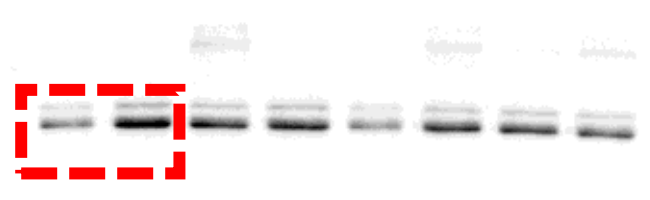

65 kDa

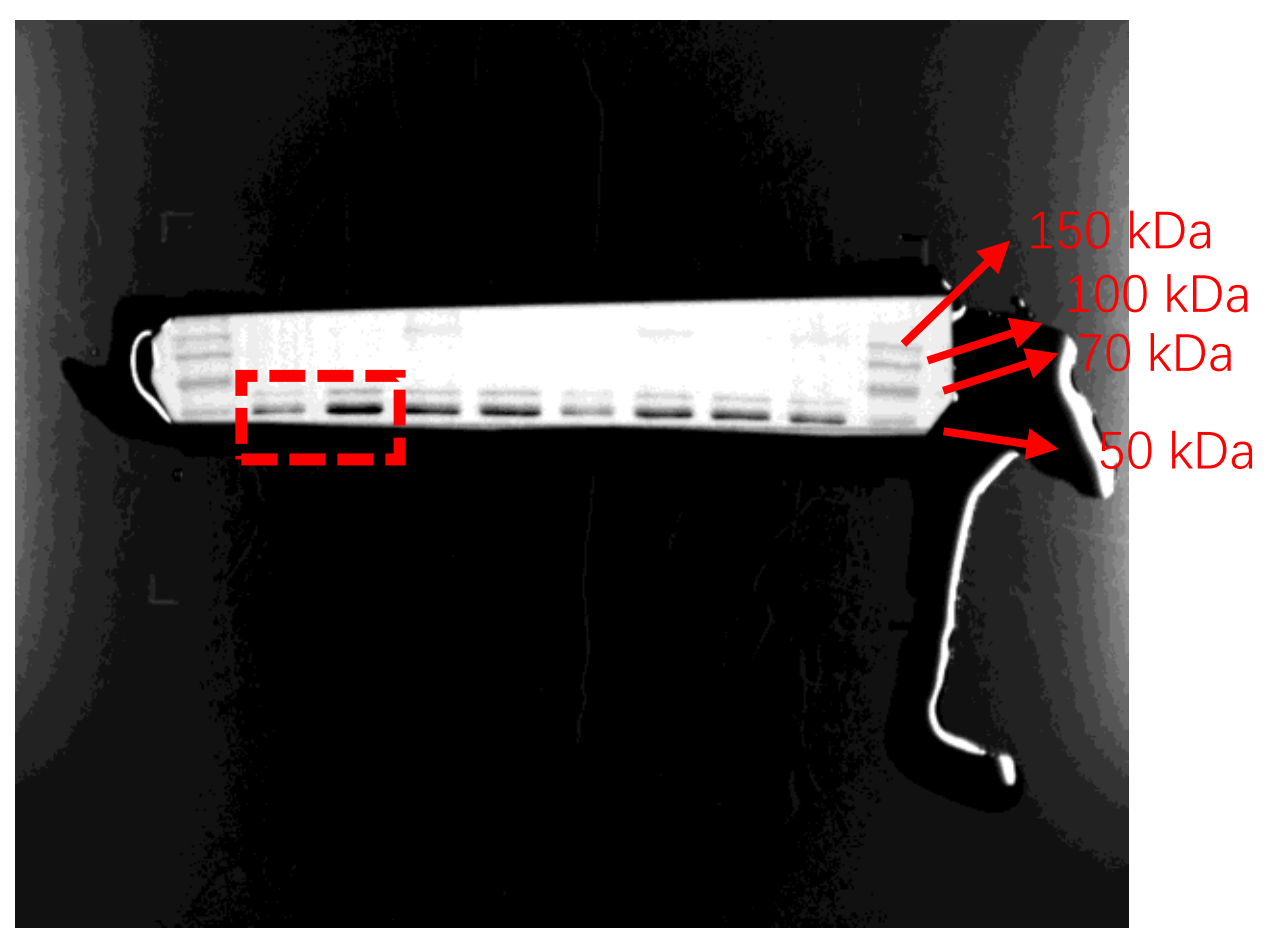

GAPDH

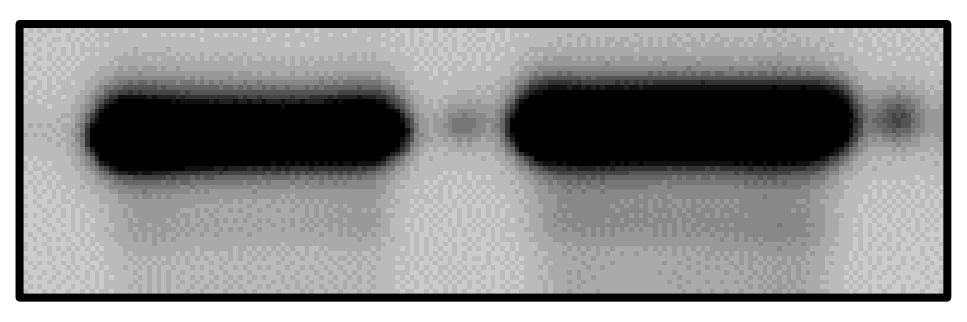

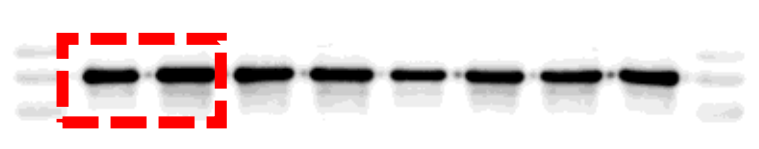

36 kDa

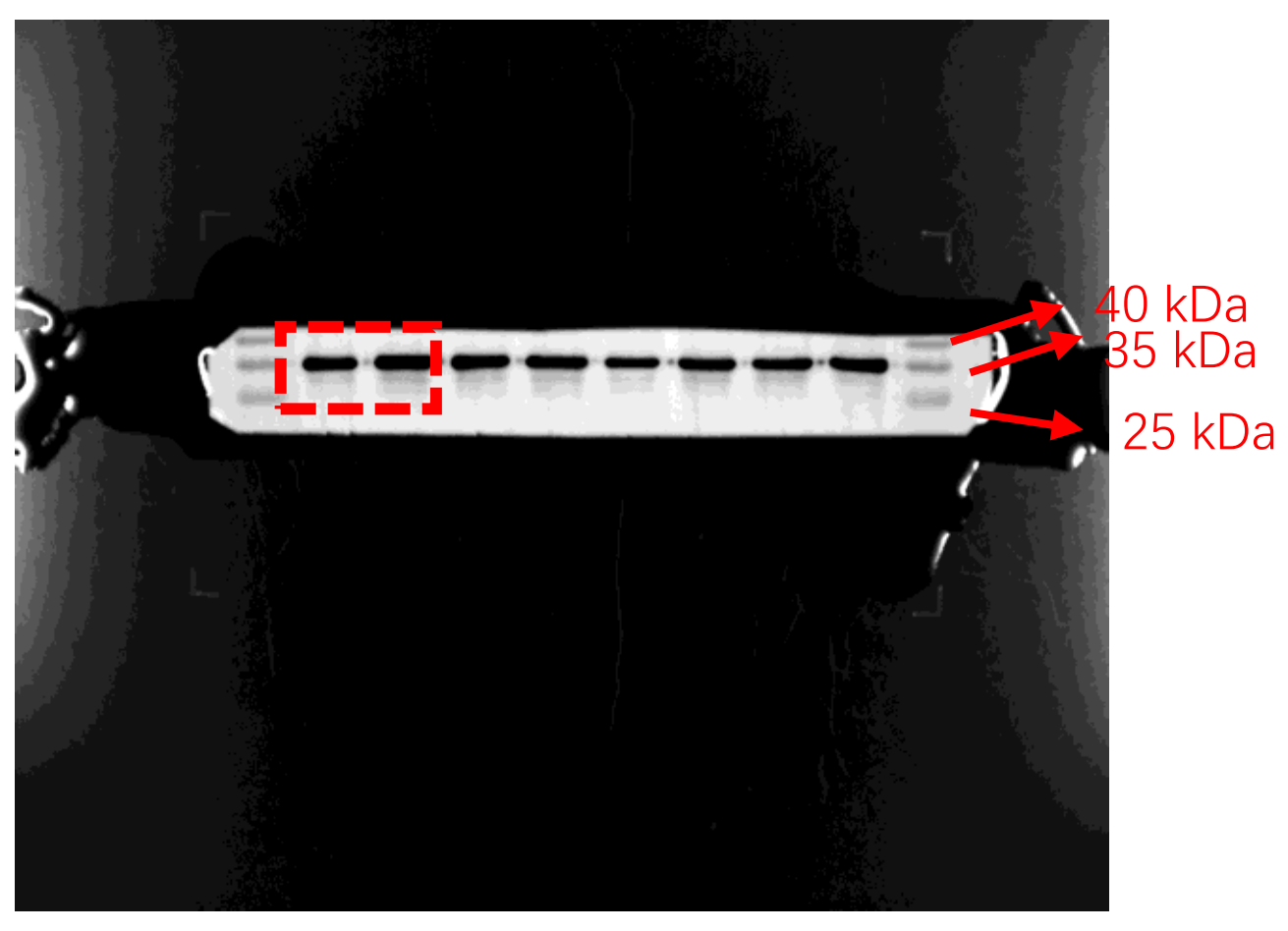


Dynamin

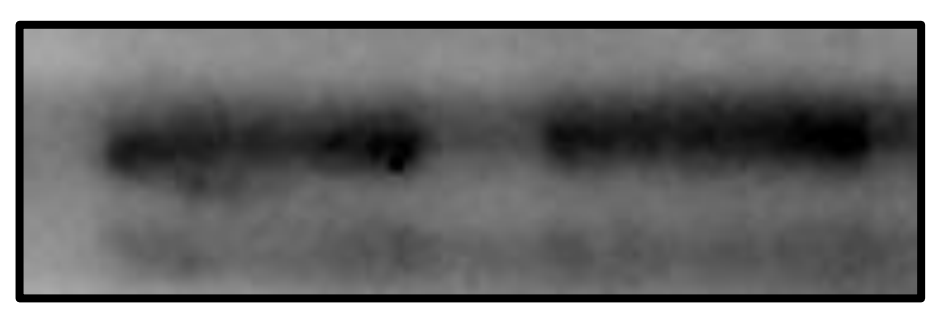

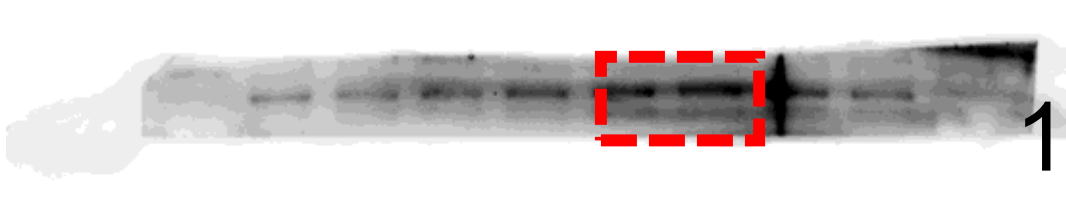

100 kDa

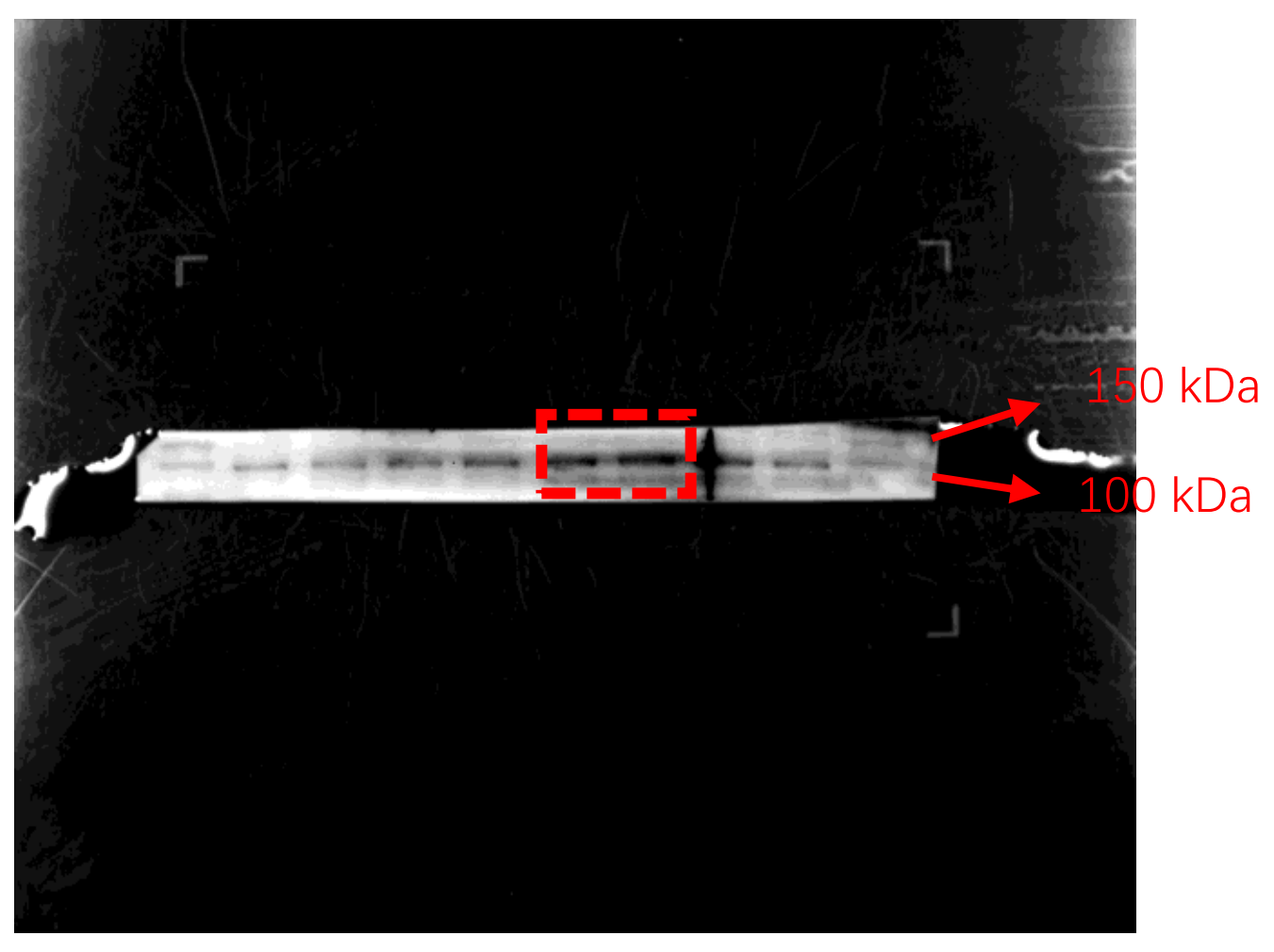

GAPDH

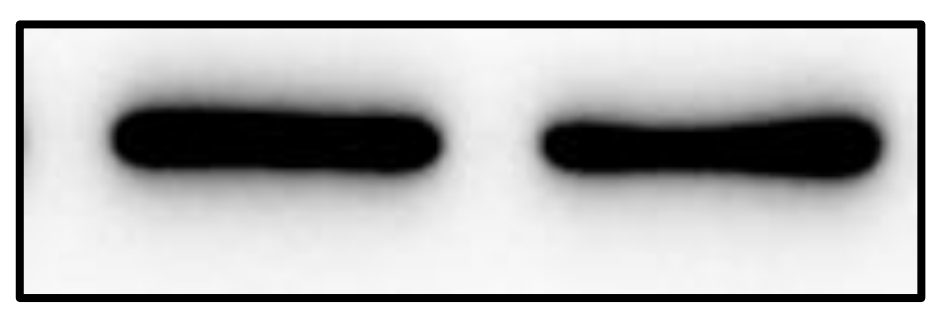

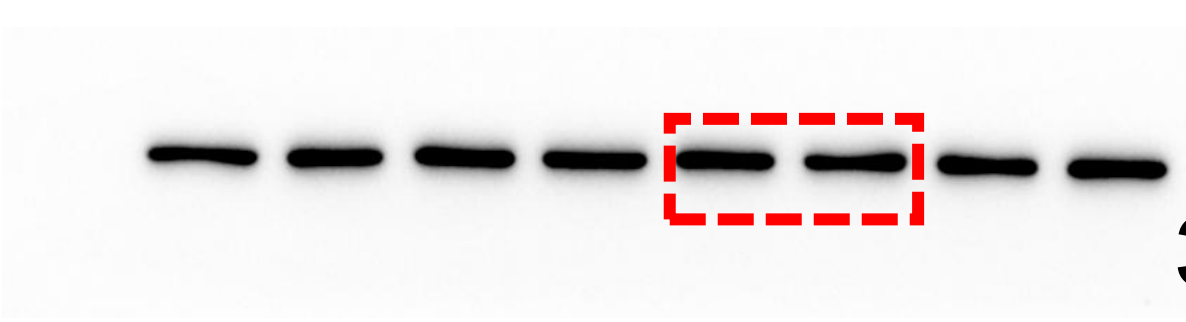

36 kDa

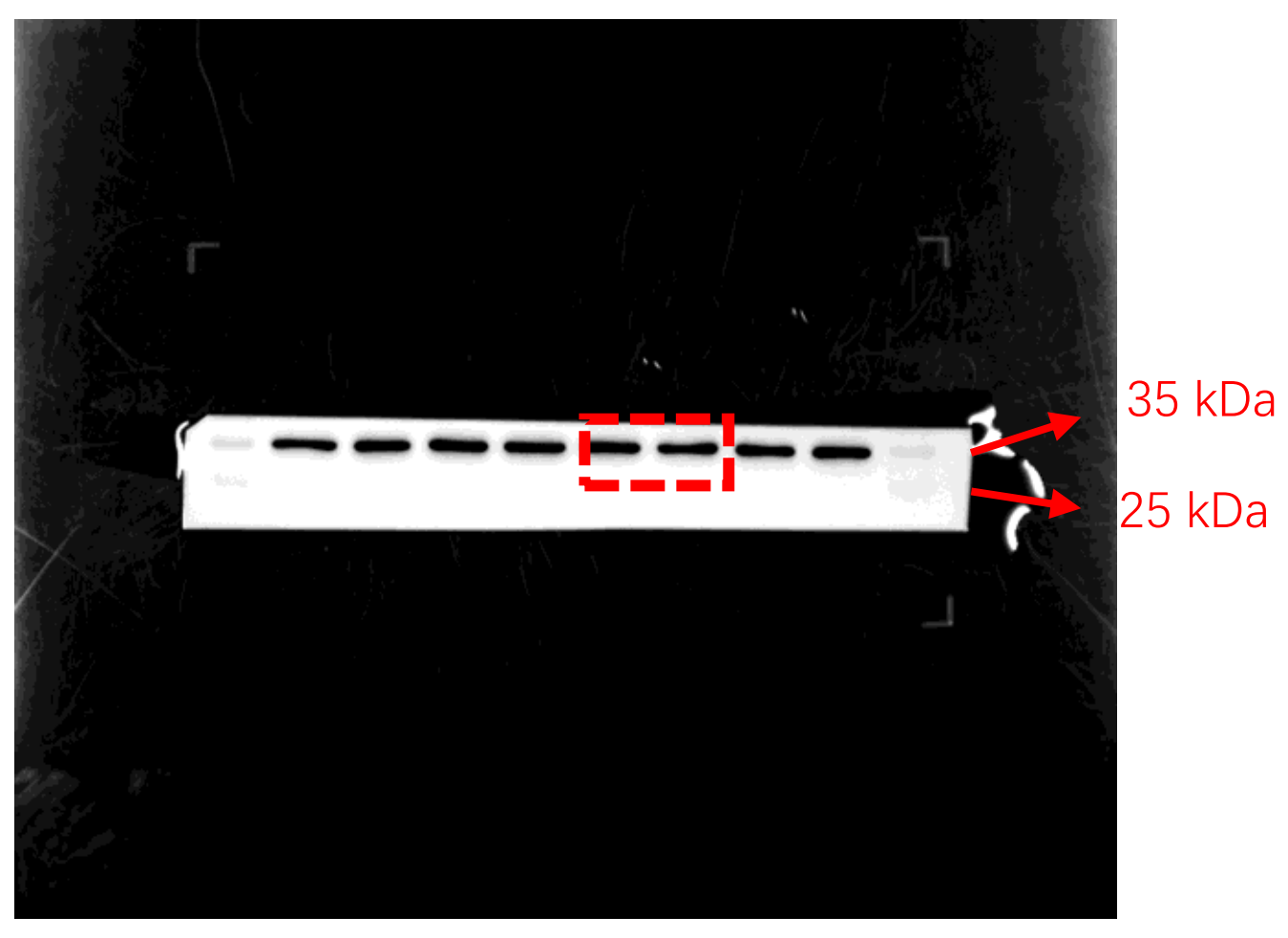

Figure 3 b

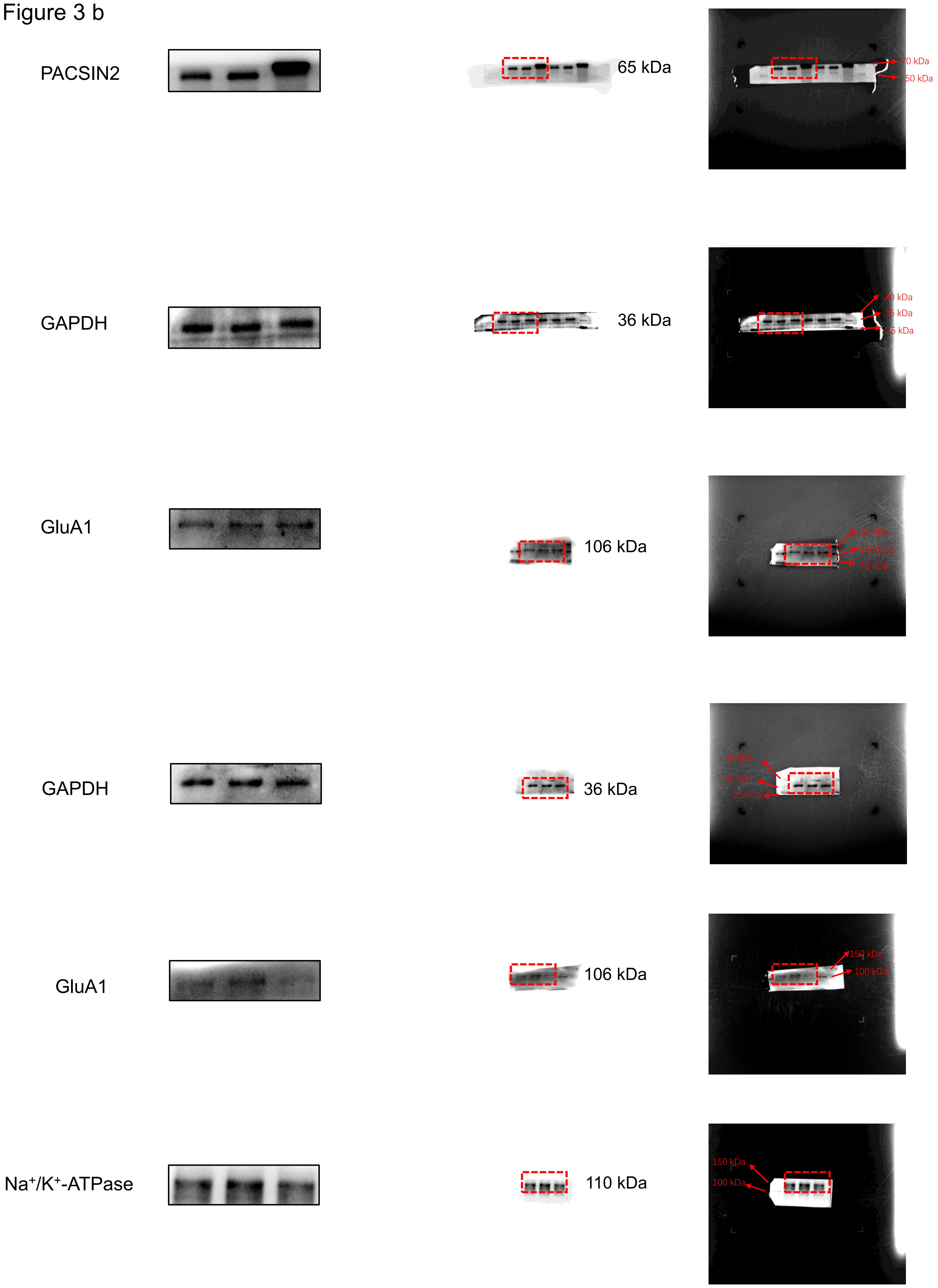
PACSIN2
65 kDa
50 kDa
GAPDH
36 kDa
GluA1
106 kDa
GAPDH
36 kDa
GluA1
106 kDa
150 kDa
100 kDa
Na+/K+-ATPase
110 kDa
150 kDa
100 kDa

Fig. 4a

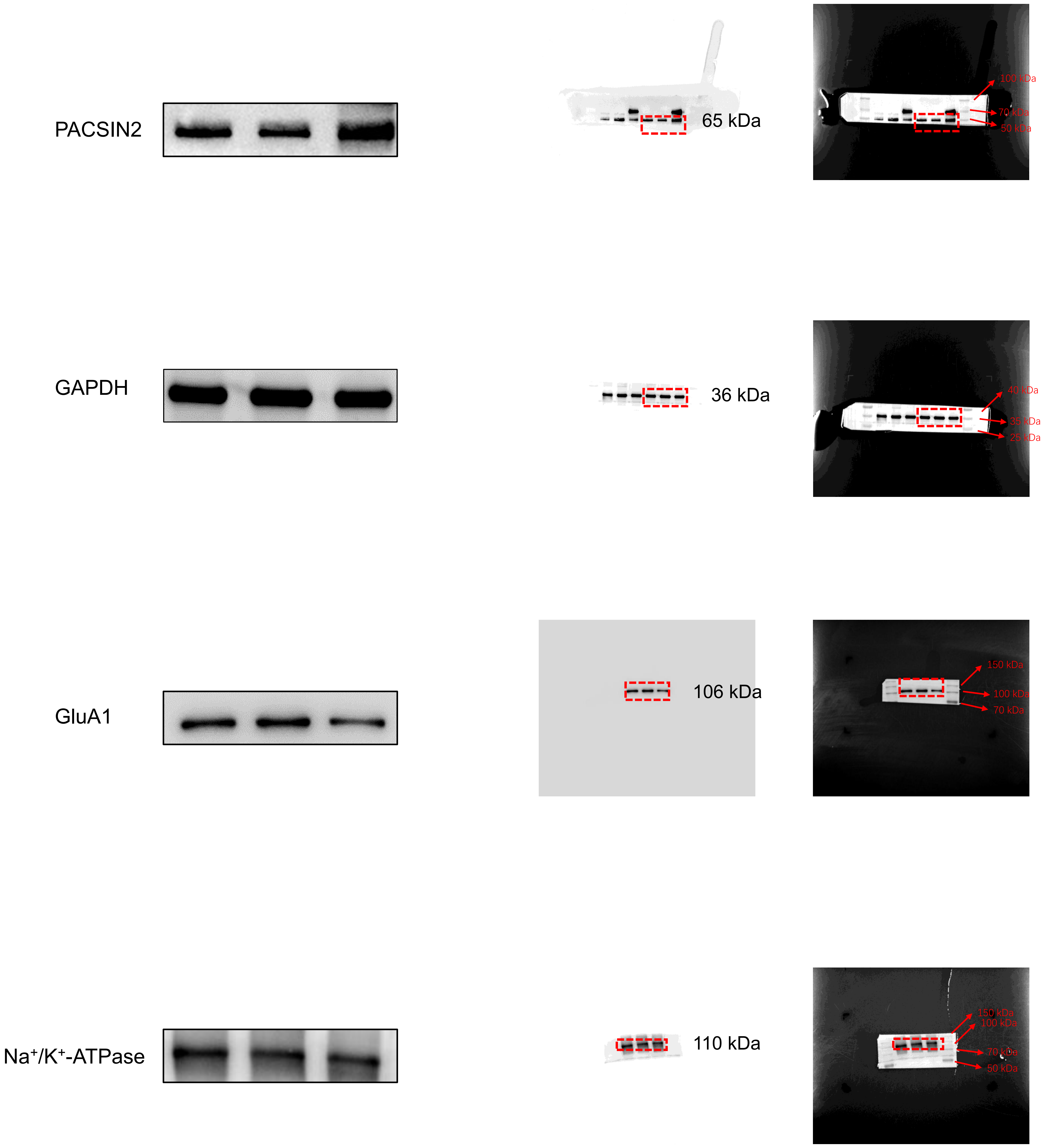

Figure 6 d

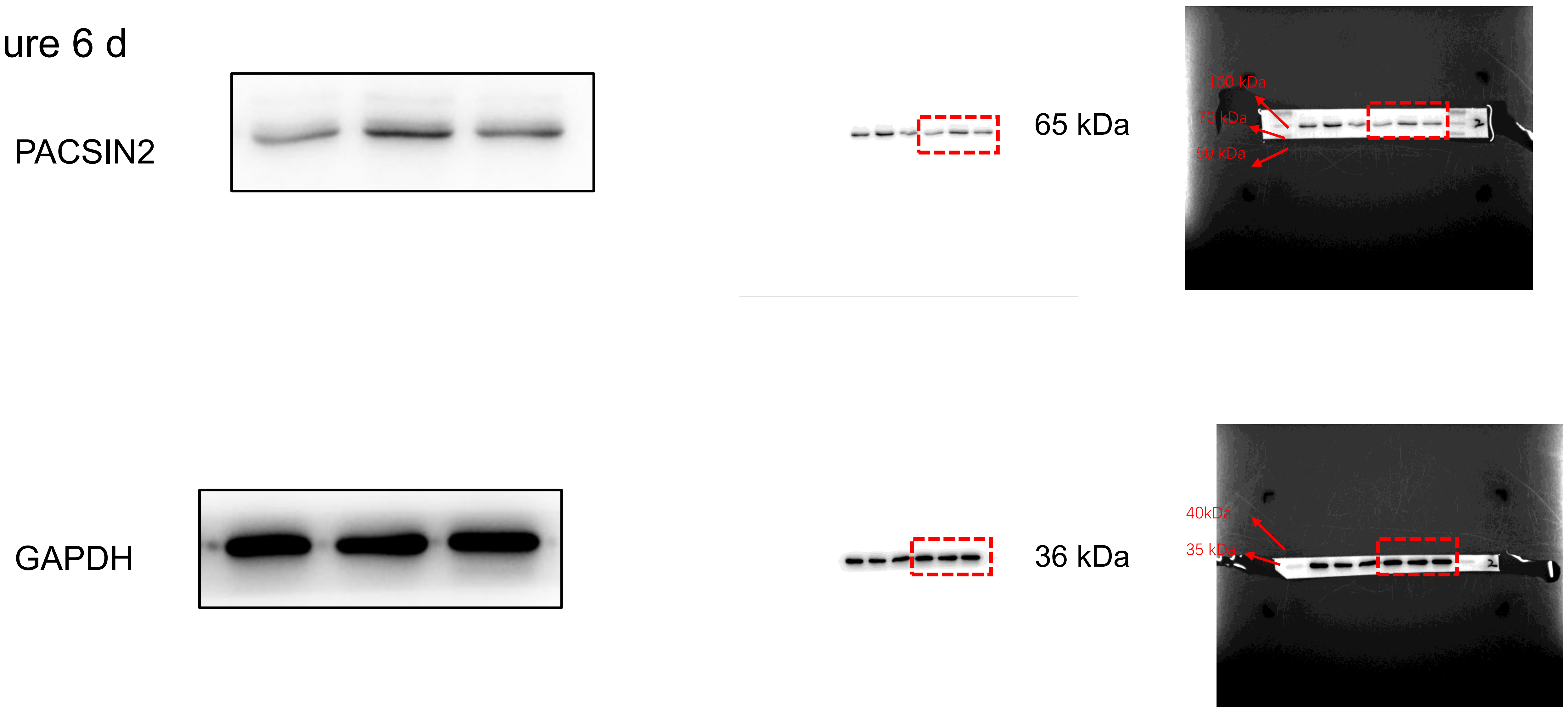


Figure 6 f

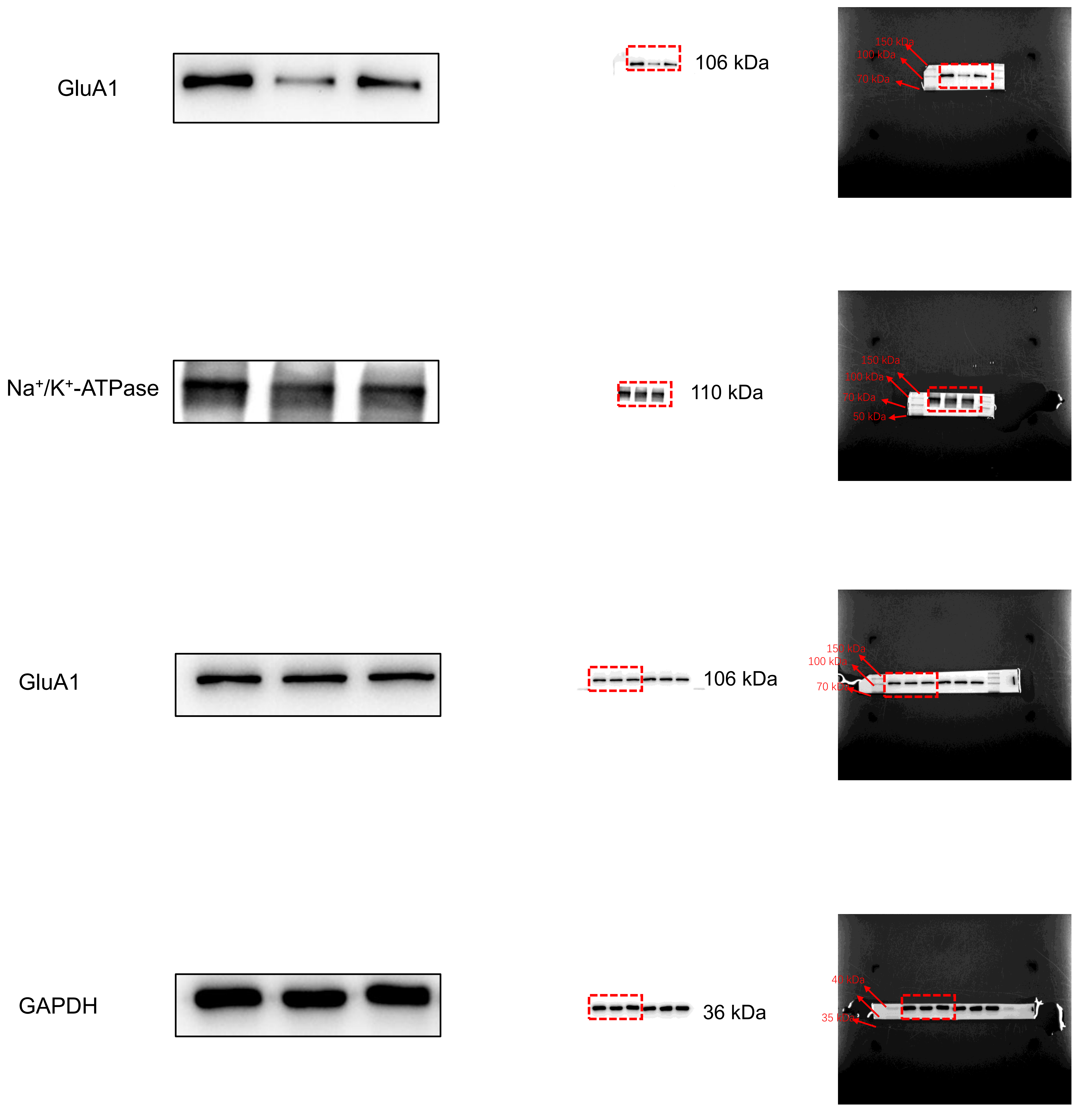

Source Data Supplementary Fig. 5e

PACSIN2 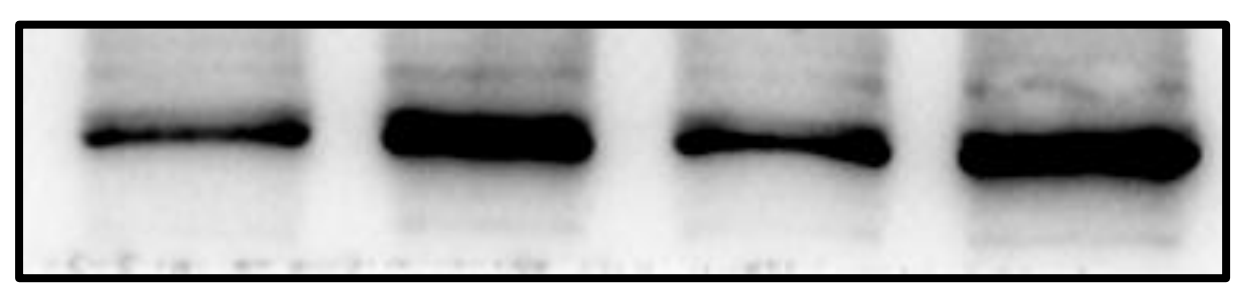

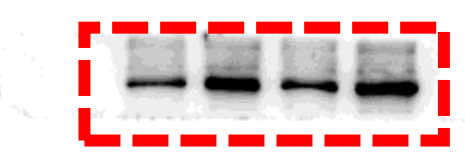

65 kDa

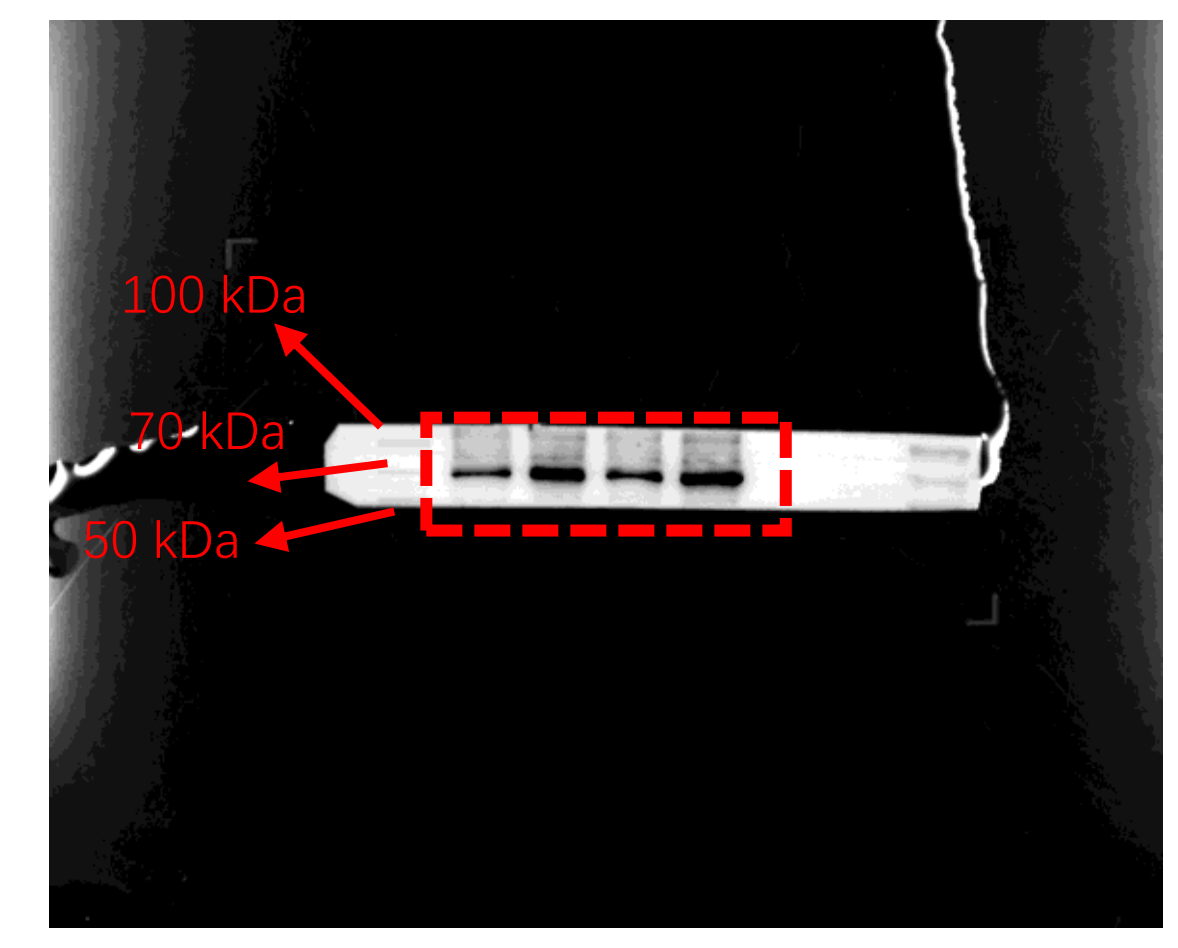


GAPDH 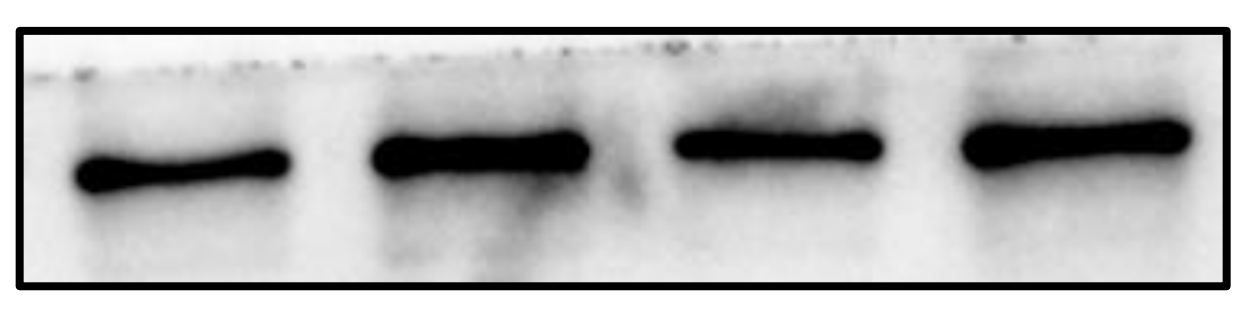

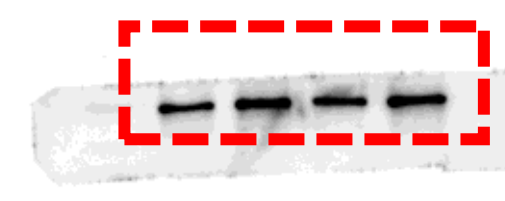

36 kDa

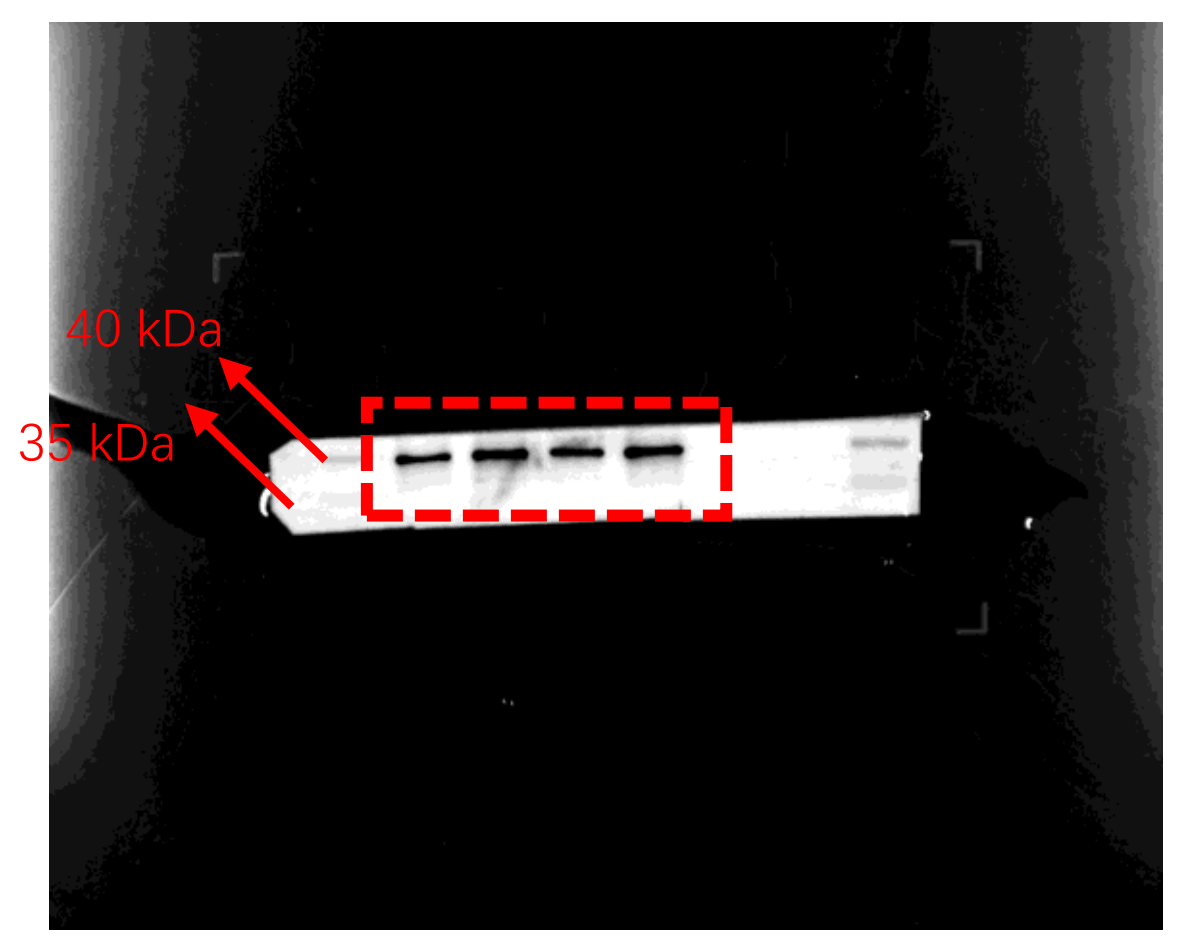

Source Data Supplementary Fig. 6a

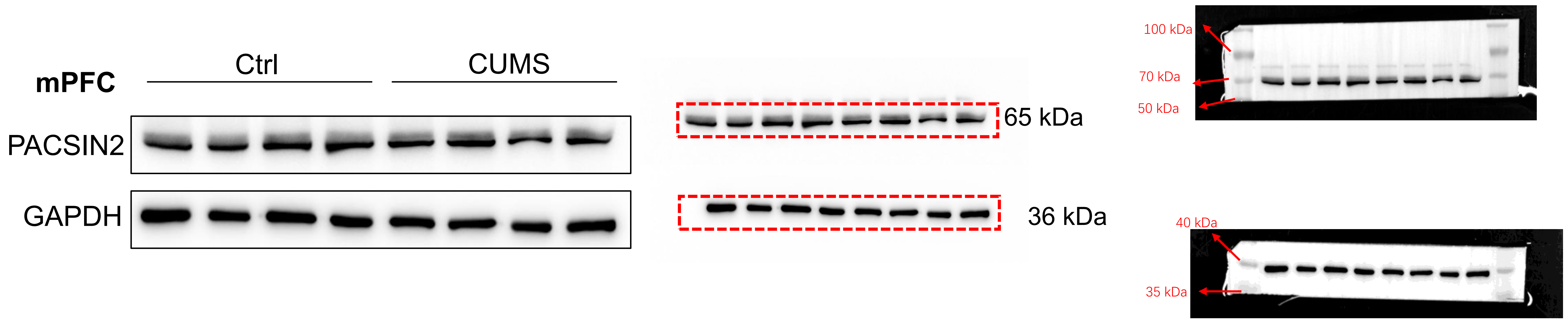


Source Data Supplementary Fig. 6c

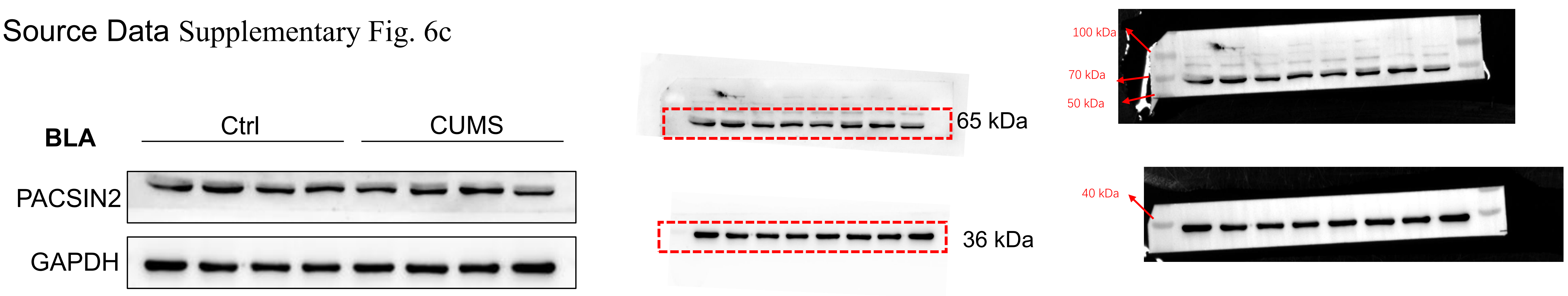

Source Data Supplementary Fig. 11a

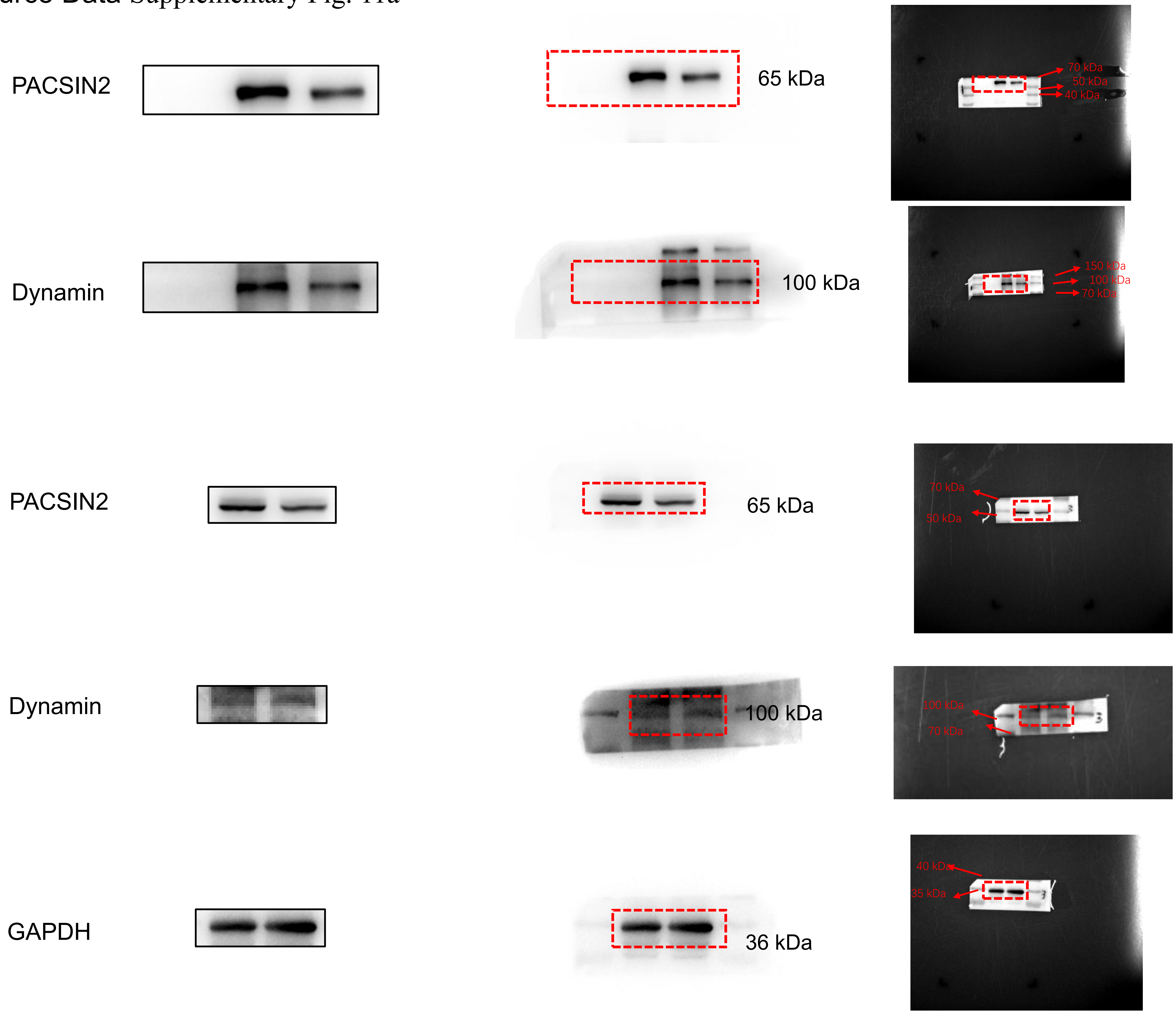


Source Data Supplementary Fig. 11c

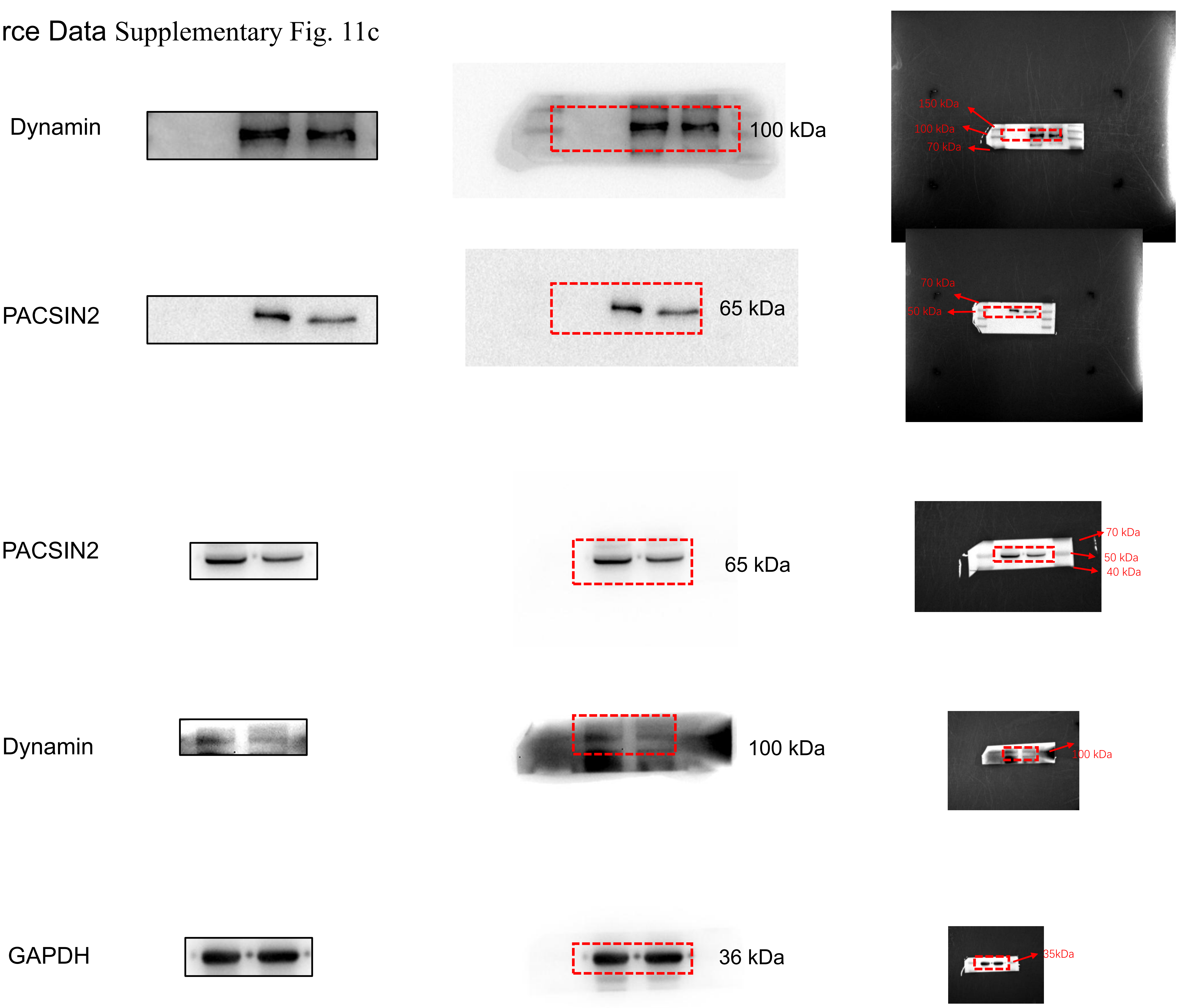

Supplement: Supplementary file 1 [file 41401_2026_1790_MOESM3_ESM.pdf]
