# Supplementary material for: PACSIN2-mediated synaptic injury contributes to behavioral disorders caused by chronic stress
Source: Acta Pharmacol Sin. 2026 May 7;47(9):2358–76. doi: 10.1038/s41401-026-01790-0 (PMC13487163; doi:10.1038/s41401-026-01790-0)
Supplement: Supplementary file 2 [file 41401_2026_1790_MOESM1_ESM.docx]

**Supplementary Materials**

**PACSIN2-mediated synaptic** **injury contributes to behavioral disorders caused by chronic stress**

Changmin Wang^1,5#^, Ye Li ^1,5#^, Yuhang Yi^1,5^, Xiao Chen^1,5^, Tian Lan^1,5^, Haiyan Lou^2^, Wencheng Jian^3^﹡, Meijian Wang^4^﹡, Shuyan Yu ^1, 5^﹡

1. Department of Physiology, School of Basic Medical Sciences, Cheeloo College of Medicine, Shandong University, Jinan, Shandong, 250012, China.

2. Department of Pharmacology, School of Basic Medical Sciences, Cheeloo College of Medicine, Shandong University, Jinan, Shandong, 250012, China.

3. Department of Radiology, Qilu Hospital, Shandong University, Jinan, Shandong, 250001, China.

4. Department of Endocrinology and Metabolism, The Third QiLu Hospital of Shandong University, Jinan, Shandong, 250001, China.

5. Shandong Key Laboratory of Mental Disorders and Intelligent Control, School of Basic Medical sciences, Cheeloo College of Medicine, Shandong University, Jinan, Shandong, 250012, China.

^#^ The authors contribute equally in this work.

﹡Corresponding author: Shuyan Yu, E-mail address: shuyanyu@sdu.edu.cn

Meijian Wang, E-mail address: wangmeijian@sdu.edu.cn

Wencheng Jian, E-mail address: jianwencheng@hotmail.com

**Supplemental Figure and Figure Legends**

**Figure S1: CUMS induces** **anxiety-like behavior in rats and produces dendritic spine loss within the hippocampus of rats.**

**a** Raw traces of rats in the OFT. **b** and **c** Measurements of the total distance and the center time of rats in OFT. *n =* 10 rats per group. NS: not significant *P* > 0.05, ****P* < 0.001 (Two-tailed unpaired t-test). **d** Raw traces of rats in the EPM. **e** and **f** The open arm time and entries of rats in EPM. *n =* 10 rats per group. ****P* < 0.001 (Two-tailed unpaired t-test (nonparametric tests). **g** Schematic of the AAV virus injection method used to sparsely label neurons. **h** Representative images showing sparsely labelled neurons in the hippocampus. Scale bar, 100 μm. Representative confocal stacks and three-dimensional reconstruction images of the dendrites of hippocampal pyramidal neurons. Scale bar, 10 μm. The white arrow indicates the mushroom-shaped dendritic spines. **i** Total spine density of Ctrl and CUMS rats. n = 24 dendrites from 12 cells from eight slices from four rats per group. ****P* < 0.001 (Two-tailed unpaired t-test). **j** Mushroom spine density of Ctrl and CUMS rats. n = 24 dendrites from 12 cells from eight slices from four rats per group. ****P* < 0.001 (Two-tailed unpaired t-test). **k** Representative images of Bassoon and GluA1 intensity on CA1 hippocampus from ctrl and CUMS rats (GluA1, green; Bassoon, red; DAPI, blue). Scale bars, 10 μm. **l** A box plot (left) and kernel density estimate (right) of signal intensity of Bassoon in Ctrl (blue) and CUMS (orange) rats. a.u., arbitrary units. n = 16 slices from four rats. ****P* < 0.001 (Two-tailed unpaired t-test). **m** A box plot (left) and kernel density estimate (right) of signal intensity of GluA1 in Ctrl (blue) and CUMS (orange) rats. a.u., arbitrary units. n = 16 slices from four rats. ****P* < 0.001 (Two-tailed unpaired t-test). Data are represented as the mean ± SEM. Ctrl, control; CUMS, chronic unpredictable mild stress. OFT: open field test; EPM: the elevated plus maze.

**Figure S2: CUMS produces induces maladaptation of hippocampal synaptic function. a** Schematic diagram showing the chronic unpredictable mild stress (CUMS) procedure. **b** Representative images showing expression of c-Fos in the dCA1 region of hippocampus of rats. Scale bar, 100 μm. **c** Quantification of the number of c-FOS^+^ cells in the dCA1 region of hippocampus of rats. *n =* 6 slices from three rats per group. **P* < 0.05 (two-tailed unpaired t-test). **d** Representative sEPSCs traces in CA1 hippocampal pyramidal neurons. Summary data for sEPSCs amplitude **e** and frequency **f** in CA1 hippocampal pyramidal neurons of Ctrl and CUMS rats. *n =* 8 neurons from three rats per group. ***P* < 0.01 (Two-tailed unpaired t-test). **g** Quantification of the AP threshold (mV) in CA1 hippocampal pyramidal neuron of Ctrl and CUMS rats. *n =* 6 neurons from three rats per group. ***P* < 0.01 (Two-tailed unpaired t-test). **h** Quantification of input resistance in CA1 hippocampal pyramidal neuron of Ctrl and CUMS rats. *n =* 6 neurons from three rats per group. ***P* < 0.01 (Two-tailed unpaired t-test). **i** Quantification of membrane capacitance in CA1 hippocampal pyramidal neuron of Ctrl and CUMS rats. *n =* 6 neurons from three rats per group. ***P* < 0.01 (Two-tailed unpaired t-test). **j** Quantification of half-width in CA1 hippocampal pyramidal neuron of Ctrl and CUMS rats. *n =* 6 neurons from three rats per group. ***P* < 0.01 (Two-tailed unpaired t-test). Data are represented as the mean ± SEM. sEPSCs: spontaneous excitatory postsynaptic currents.

**Figure S3: DREADD inhibition of CA1 hippocampal pyramidal neurons induces depression-like behaviors in rats.**

**a-b** Experimental design of studies and Schematics of AAV vectors and bilateral injection sites in the hippocampus. Scale bar, 500 μm (*top*) and 50 μm (*bottom*). Diagram of CNO (5 mg/kg) injection procedure in rats. i.p., intraperitoneal. **c** Immobility time of rats in FST with (purple) and without (blue) CNO treatment. *n =* 8 rats for each group. NS: not significant *P* > 0.05, ****P* < 0.001 (Two-way repeated measures ANOVA with Sidak's multiple comparisons). **d** The sucrose preference of rats in SPT. *n =* 8 rats for each group. NS: not significant *P* > 0.05, **P* < 0.05 (Two-way repeated measures ANOVA with Sidak's multiple comparisons). **e-f** The total distance and the center time of rats in OFT. *n =* 8 rats for each group. NS: not significant *P* > 0.05, ***P* < 0.01 (Two-way repeated measures ANOVA with Sidak's multiple comparisons). **g-h** The time and entries of rats in open arm of EPM. *n =* 8 rats for each group. NS: not significant *P* > 0.05, ***P* < 0.01, ****P* < 0.001 (Two-way repeated measures ANOVA with Sidak's multiple comparisons). **i** Whole-cell patch-clamp recording from CA1 hippocampal pyramidal neurons in brain slices. **j** Representative voltage responses in CA1 pyramidal neurons in mCherry rats to injection currents from 0 to 100 pA with 20-pA steps before and after bath application of CNO (10 μM). **k** Representative voltage responses in CA1 pyramidal neurons in hM4di rats to injection currents from 0 to 100 pA with 20-pA steps before and after bath application of CNO (10 μM). Red traces indicate the minimal current needed to induce Aps. Scale, 10 mV; 500 ms. **l** Quantification of the rheobase (pA) in CA1 hippocampal pyramidal neurons before and after bath application of CNO. *n =* 6 neurons from three rats). ****P* < 0.001 (Two-tailed paired t-test). **m** Quantification of the input resistance in CA1 hippocampal pyramidal neurons before and after bath application of CNO. *n =* 6 neurons from three rats). ****P* < 0.001 (Two-tailed paired t-test). **n** and **o** Quantification of AP numbers of CA1 hippocampal pyramidal neurons. *n =* 6 neurons from three rats. ****P* < 0.001 (Two-way repeated measures ANOVA with Sidak's multiple comparisons). Data are represented as the mean ± SEM. CNO: Clozapine-N-oxide.

**Figure S4:** **Proteomic analysis of hippocampal regions in rats.**

**a** The heatmap diagram of differentially expressed protein levels in the dCA1region of hippocampus of control and CUMS rats as obtained from proteomic analysis. **b** Histogram of Kyoto Encyclopedia of Genes and Genomes (KEGG) pathways of the differentially expressed proteins at level 1. **c** Histogram of Kyoto Encyclopedia of Genes and Genomes (KEGG) pathways of the differentially expressed proteins at level 2.

**Figure S5: CUMS** **increases PACSIN2 protein expression in** **CA1 region of hippocampus.**

**a** Representative images of PACSIN2 intensity on dCA1 hippocampus (PACSIN2, green; MAP2, red; DAPI, blue). Scale bars: 50 μm. **b** Quantification of PACSIN2 expression by Pearson's correlation in hippocampus of rats. *n =* 8 from eight slices from four rats per group; ***P* < 0.01 (Two-tailed unpaired t-test (nonparametric tests), Mann-Whitney U test). **c** Immobility time of rats in FST. *n =* 6 rats per group. NS: not significant, ***P* < 0.01, ****P* < 0.001 (One-way ANOVA with Tukey’s multiple comparison tests). **d** The measurement of sucrose preference of rats. *n =* 6 rats per group. NS: not significant, ****P* < 0.001 (One-way ANOVA with Tukey’s multiple comparison tests). **e** and **f** The PACSIN2 expression levels in hippocampus of rats. *n =* 5 rats per group. NS: not significant *P* > 0.05, **P* < 0.05, ***P* < 0.01 (One-way ANOVA with Tukey’s multiple comparison tests). **g** Representative images of the PACSIN2 expression in CA1 region of hippocampus (PACSIN2, green; DAPI, blue). Scale bars = 50 μm. **h** Quantification of mean PACSIN2 intensity in hippocampus. *n =* 5 rats per group. NS: not significant *P* >0.05, ***P* < 0.01, ****P* < 0.001 (One-way ANOVA with Tukey’s multiple comparison tests). Data are represented as the mean ± SEM. Ctrl, control; CUMS, chronic unpredictable mild stress rats; FLX, Fluoxetine.

**Figure S6: CUMS does not change the expression levels of PACSIN2 in mPFC or BLA.**

**a**-**b** The expression levels of PACSIN2 protein from mPFC. n = 4 rats per group. NS: not significant *P* > 0.05, (Two-tailed unpaired t-test). **c**-**d** The expression levels of PACSIN2 protein from BLA. n = 4 rats per group. NS: not significant *P* > 0.05, (Two-tailed unpaired t-test). Data are represented as the mean ± SEM.

**Figure S7: Overexpression of PACSIN2 increases colocalization of PACSIN2 and Dynamin and reduces the density of excitatory synapses in primary neurons.**

**a** Representative fluorescence images of primary neurons transfected with lentiviruses. Scale bars, 100 μm. **b** Example three-channel images of DIV14 primary neurons infected at DIV6 with LV-eGFP (pcSLenti-EF1-EGFP-CMV-MCS-3×FLAG-WPRE) and LV-PACSIN2 (pcSLenti-EF1-EGFP-CMV-PACSIN2-3×FLAG-WPRE). From left to right: image of the neuron-filling GFP, PACSIN2 stained with Alexa Fluor 568 (magenta), dynamin stained with Cy5TM (red). Scale bars, 10 μm. **c** Quantification of colocalization Ratio between PACSIN2 and dynamin in the primary neurons transfected with lentiviruses. *n =*18 per group. ****P* < 0.001(Two-tailed unpaired t-test). **d** and **e** Representative double-IF stain using VGLUT1^+^ (green) as presynaptic and PSD-95^+^ (red) as postsynaptic markers of excitatory neurons. VGLUTI^+^/PSD95^+^ colocalizing synapses are magnified (right). The scatterplot shows the density (numbers per 50 μm) of VGLUTI^+^/PSD95^+^ synapses in DIV16 primary neurons in WT and LV-PACSIN2 groups. Scale bars, 10 μm (left); 5 μm (right). *n =* 28 from fifteen neurons per group. ****P* < 0.001 (Two-tailed unpaired t-test). Data are represented as the mean ± SEM.

**Figure S8: The EGFP viral vector does not affect** **behavioral phenotypes in WT or CUMS rats.**

**a** Experimental design studies. **b** The measurement of sucrose preference of WT and AAV-eGFP group rats. n = 7 rats each group. NS: not significant *P* > 0.05 (Two-tailed unpaired t-test). **c** Immobility time of rats in FST. n = 7 rats each group. NS: not significant *P* > 0.05 (Two-tailed unpaired t-test). **d** Raw traces of rats in the OFT. **e** and **f** Measurements of the total distance and the center time of rats in OFT. *n =* 7 rats per group. NS: not significant *P* > 0.05 (Two-tailed unpaired t-test). **g** Raw traces of rats in the EPM. **h** and **i** The open arm time and entries of rats in EPM. *n =* 7 rats per group. NS: not significant *P* > 0.05 (Two-tailed unpaired t-test). **j** Experimental design studies. **k** The measurement of sucrose preference of CUMS and CUMS/eGFP group rats. n = 7 rats each group. NS: not significant *P* > 0.05 (Two-tailed unpaired t-test). **l** Immobility time of rats in FST. n = 7 rats each group. NS: not significant *P* > 0.05 (Two-tailed unpaired t-test). **m** Raw traces of rats in the OFT. **n** and **o** Measurements of the total distance and the center time of rats in OFT. *n =* 7 rats per group. NS: not significant *P* > 0.05 (Two-tailed unpaired t-test). **p** Raw traces of rats in the EPM. **q** and **r** The open arm time and entries of rats in EPM. *n =* 7 rats per group. NS: not significant *P* > 0.05 (Two-tailed unpaired t-test). Data are represented as the mean ± SEM.

**Figure S9: Overexpression of PACSIN2 in CA1 hippocampal does not induce anxiety-like behavior in rats.**

**a** Raw traces of rats in the OFT. **b** and **c** Measurements of the total distance and the center time of rats in OFT. *n =* 7 rats each group. NS: not significant *P* > 0.05 (One-way ANOVA with Tukey's multiple comparisons test). **d** Raw traces of rats in the EPM. **e** and **f** Measurements of the open arm time and entries of rats in EPM *n =* 7 rats each group. NS: not significant *P* > 0.05 (One-way ANOVA with Tukey's multiple comparisons test). Data are represented as the mean ± SEM. OFT: open field test; EPM: the elevated plus maze.

**Figure S10: Overexpression of PACSIN2 in CA1 hippocampal enhances the susceptibility to stress in rats.**

**a** Raw traces of rats in the OFT. **b** and **c** Measurements of the total distance and the center time of rats in OFT. *n =* 7 rats each group. NS: not significant *P* > 0.05, ***P* < 0.01 (One-way ANOVA with Tukey's multiple comparisons test). **d** Raw traces of rats in the EPM. **e** and **f** Measurements of the open arm time and entries of rats in EPM *n =* 7 rats each group. NS: not significant *P* > 0.05, **P* < 0.05, ****P* < 0.001 (One-way ANOVA with Tukey's multiple comparisons test). **g** Raw traces of rats in the OFT. **h** and **i** Measurements of the total distance and the center time of rats in OFT. *n =6* rats each group. NS: not significant *P* > 0.05, **P* < 0.05 (Two-tailed unpaired t-test). **j** Raw traces of rats in the EPM. **k** and **l** Measurements of the open arm time and entries of rats in EPM. *n =6* rats each group. Open arm time: NS: not significant *P* > 0.05 (Two-tailed unpaired t-test); Open arm entries: NS: not significant *P* > 0.05 (Two-tailed unpaired t-test (nonparametric tests), Mann-Whitney U test). Data are represented as the mean ± SEM. OFT: open field test; EPM: the elevated plus maze.

**Figure S11: Knockdown of PACSIN2 in CA1 hippocampal reduces the degree of interaction between PACSIN2 and dynamin in CUMS rats.**

**a** and **c** Representative co-immunoprecipitation (co-IP) of PACSIN2 and Dynamin of the dCA1 region of hippocampus in rats. **b** and **d** Co-IP analysis of PACSIN2 and Dynamin in rats. *n =* 4 rats per group. **P* < 0.05 (Two-tailed unpaired t-test). Data are represented as the mean ± SEM.

**Figure S12: Knockdown of PACSIN2 in CA1 hippocampal has no effects on anxiety-like behaviors in CUMS rats.**

**a** Representative images staining of PACSIN (green), dynamin (red) and DAPI (blue) in the dCA1 region of hippocampus. PACSIN2^+^/dynamin^+^ colocalizing are magnified (right). Scale bars = 10 μm. **b** Quantification of colocalization Ratio between PACSIN2 and dynamin in the hippocampus of WT, CUMS/eGFP, and CUMS/shPACSIN2 group rats. *n =* 6 slices from three rats per group. NS: not significant, *P* > 0.05, ***P* < 0.01 (One-way ANOVA with Tukey’s multiple comparison tests). **c** Raw traces of rats in the OFT. **d-e** Measurements of the total distance and the center time of rats in OFT. WT: *n =* 8 rats; CUMS/eGFP: *n =* 8 rats; CUMS/shPACSIN2 groups: *n =* 8 rats. NS: not significant, ****P* < 0.001 (One-way ANOVA with Tukey’s multiple comparison tests). **f** Raw traces of rats in the EPM. **g-h** Measurements of the open arm time and entries of rats in EPM. WT: *n =* 8 rats; CUMS/eGFP: *n =* 8 rats; CUMS/shPACSIN2 groups: *n =* 8 rats. NS: not significant, **P* < 0.01, ****P* < 0.001 (One-way ANOVA with Tukey’s multiple comparison tests). Data are represented as the mean ± SEM. OFT: open field test; EPM: the elevated plus maze.
