# Supplementary material for: PACSIN2-mediated synaptic injury contributes to behavioral disorders caused by chronic stress
Source: Acta Pharmacol Sin. 2026 May 7;47(9):2358–76. doi: 10.1038/s41401-026-01790-0 (PMC13487163; doi:10.1038/s41401-026-01790-0)
Supplement: Supplementary file 3 [file 41401_2026_1790_MOESM2_ESM.docx]

**Supplementary Table 1**

| REAGENT or RESOURCE | SOURCE | IDENTIFIER |
| --- | --- | --- |
| Antibodies | | |
| Mouse anti-MAP2 | Cell Signaling Technology | Cat# 4542S |
| Mouse anti-PSD-95 | Santa Cruz Biotechnology Technology | Cat# sc-32290 |
| Mouse anti-PACSIN2 | Santa Cruz Biotechnology | Cat# sc-390136 |
| Mouse anti-Dynamin | Santa Cruz Biotechnology | Cat# sc-17807 |
| Rabbit anti-PACSIN2 | Proteintech Group | Cat# 10518-2-AP |
| Rabbit anti-GluA1 | Affinity | Cat# AF6303 |
| Mouse anti-GluA1 | Santa Cruz Biotechnology | Cat# SC555509 |
| Rabbit anti-VGLUT1 | Bioworld Technology | Cat# BS75067 |
| Rabbit anti-cFos | Cell Signaling Technology | Cat# 2250T |
| Rabbit anti-GluN2A | Abways Technology | Cat# CY6968 |
| Rabbit anti-GluN1 | Abways Technology | Cat# CY6798 |
| Rabbit anti-GluA2 | Abways Technology | Cat# CY7063 |
| Rabbit anti-Bassoon | Cell Signaling Technology | Cat# D63B6 |
| Rabbit anti-Na+/K+-ATPase | Proteintech | Cat# 14418-1-AP |
| Rabbit anti-GAPDH | Proteintech | Cat# 10494-1-AP |
| Peroxidase-conjugated antibodies goat anti-rabbit IgG | Zhongshan Golden Bridge Biotechnology | Cat# ZB-2301 |
| Peroxidase-conjugated goat anti-mouse | Zhongshan Golden Bridge Biotechnology | Cat# ZB-2305 |
| Donkey anti-Rabbit IgG (H+L) Highly Cross-Adsorbed Secondary Antibody, Alexa Fluor™ 568 | Thermo Fisher Scientific | Cat# A10042 |
| Alexa Fluor 488 goat anti-mouse immunoglobulin G (IgG) | Proteintech | Cat# SA00013-1 |
| DAPI | Beyotime | Cat# C1002 |
| Cy5TM goat anti-mouse IgG | Invitrogen | Cat# 2387487 |
| Alexa Fluor 488 goat anti-rabbit immunoglobulin G (IgG) | Proteintech | Cat# SA00013-2 |
| IgG | Bioworld Technology | Cat# BD0050 |
| Protein A/G Plus-Agarose | Santa Cruz Biotechnology | Cat# ac-2003 |
| Chemicals, peptides, and recombinant proteins | | |
| CX546 | MCE | Cat# HY-12505 |
| Clozapine N-oxide dihydrochloride | MCE | Cat# HY-17366A |
| Dynasore | MCE | Cat# HY-15304 |
| Fluoxetine | MCE | Cat# [F189157](https://www.aladdin-e.com/zh_cn/f189157.html) |
| Critical commercial assays | | |
| Membrane and Cytosol Protein Extraction Kit | Thermo Fisher Scientific | Cat# 89842 |
| [BeyoECL Plus (Ultra sensitive ECL chemiluminescence kit)](https://www.beyotime.com/product/P0018S.htm) | Beyotime | Cat# P0018S |
| BCA Protein Assay Kit | cwbio | Cat# CW0014S |
| Pierce™ Classic IP Kit | Thermo Scientific™ | Cat# 26146 |
| High sensitive ECL chemiluminescence detection kit | Vazyme | Cat# E412-01 |
| Experimental models: Organisms/strains | | |
| Wistar rats | Ji’nan Pengyue Laboratory Animal Breeding CO.,Ltd. | |
| Software and algorithms | | |
| IMARIS | Bitplane | https://imaris.oxinst.com/ |
| ImageJ | NIH IMAGEJ | https://imagej.nih.gov/ij/ |
| Graphpad prism 8 | Graphpad prism | https://www.graphpad.com/scientific-software/prism/ |
| Photoshop CC | Adobe | https://www.adobe.com/products/photoshop.html |
| Virus | SOURCE |  |
| H8142: pAAV-pTRE-tight-NLS-Cre | Obio Technology (Shanghai) Corp.,Ltd. | |
| H6884: pAAV-hSyn-DIO(tTA-P2A-mScarlet)-NLS-WPRE | Obio Technology (Shanghai) Corp.,Ltd. | |
| pAAV-CaMKIIα-hM4D(Gi)-mCherry-3xFLAG-WPRE | Obio Technology (Shanghai) Corp.,Ltd. | |
| pAAV-CMV-GCaMP6s-P2A-nls-dTomato | Obio Technology (Shanghai) Corp.,Ltd. | |
| pAAV-CaMKIIα-hM4D(Gi)-mCherry-3xFLAG-WPRE | Obio Technology (Shanghai) Corp.,Ltd. | |
| pAAV-CMV-GCaMP6s-P2A-nls-dTomato | Obio Technology (Shanghai) Corp.,Ltd. | |
| pAAV-hSyn-EGFP-MCS-3xFLAG-WPRE | Obio Technology (Shanghai) Corp.,Ltd. | |
| pAAV-hSyn-EGFP-PACSIN2-3xFLAG-WPRE | Obio Technology (Shanghai) Corp.,Ltd. | |
| pAAV-hSyn-EGFP-3xFLAG-miR30shRNA(PACSIN2)-WPRE  (targrt sequence: GCAGCTGAAGAAATTGCAAGA) | Obio Technology (Shanghai) Corp.,Ltd. | |
| pAAV-hSyn-EGFP-3xFLAG-miR30shRNA (NC)-WPRE | Obio Technology (Shanghai) Corp.,Ltd. | |
| pcSLenti-EF1-EGFP-CMV-MCS-3xFLAG-WPRE | Obio Technology (Shanghai) Corp.,Ltd. | |
| pcSLenti-EF1-EGFP-CMV-PACSIN2-3xFLAG-WPRE | Obio Technology (Shanghai) Corp.,Ltd. | |
